# Do Disadvantageous Social Contexts Influence Food Choice? Evidence From Three Laboratory Experiments

**DOI:** 10.3389/fpsyg.2020.575170

**Published:** 2020-11-06

**Authors:** Qëndresa Rramani, Holger Gerhardt, Xenia Grote, Weihua Zhao, Johannes Schultz, Bernd Weber

**Affiliations:** ^1^Center for Economics and Neuroscience, University of Bonn, Bonn, Germany; ^2^Institute of Experimental Epileptology and Cognition Research, University of Bonn Medical Center, Bonn, Germany; ^3^Department of Economics, Institute for Applied Microeconomics, University of Bonn, Bonn, Germany; ^4^Ministry of Education Key Laboratory for Neuroinformation, Clinical Hospital of Chengdu Brain Science Institute, University of Electronic Science and Technology of China, Chengdu, China

**Keywords:** social contexts, food choice, Dictator Game, Cyberball Game, performance ranking task

## Abstract

Increasing rates of obesity have fueled interest in the factors underlying food choice. While epidemiological studies report that disadvantaged social groups exhibit a higher incidence of obesity, causal evidence for an effect of social contexts on food choice remains scarce. To further our knowledge, we experimentally investigated the effect of disadvantageous social context on food choice in healthy, non-dieting participants. We used three established experimental methods to generate social contexts of different valence in controlled laboratory settings: (i) receiving varying amounts of money in a Dictator Game (DG; *n* = 40), (ii) being included or excluded in a Cyberball Game (CBG; *n* = 35), and (iii) performing well, average, or poorly in a response time ranking task (RTR; *n* = 81). Following exposure to a particular social context, participants made pairwise choices between food items that involved a conflict between perceived taste and health attributes. In line with previous research, stronger dispositional self-control (assessed via a questionnaire) was associated with healthier food choices. As expected, being treated unfairly in the DG, being excluded in the CBG, and performing poorly in the RTR led to negative emotions. However, we did not find an effect of the induced social context on food choice in any of the experiments, even when taking into account individual differences in participants’ responses to the social context. Our results suggest that—at least in controlled laboratory environments—the influence of disadvantageous social contexts on food choice is limited.

## Introduction

Increasing rates of obesity in many countries around the world and across age groups (World Health Organization, 2020) have sparked an exceptional interest in the factors underlying food choice. Psychological and neuroscientific research has shown that differences in food-related decision making such as heightened consideration of short-term rewards (e.g., taste) and a disregard or diminished consideration of longer-term abstract rewards (e.g., health) are associated with making food choices that contribute to being overweight or obese (Mela, 2001; Volkow et al., 2008; Hare et al., 2009; Rangel, 2013; Sullivan et al., 2015). In line with this, promoting healthy eating and healthy food choices has become a common measure of public policies aiming to prevent obesity (Gearhardt et al., 2012). The measures taken to promote healthy eating are, however, not equally effective across different populations and contexts (Lyn et al., 2019). This is possibly due to the complexity of food decision making and its sensitivity to several environmental and psychosocial factors (Mela, 2001). A better understanding of the factors influencing food-related decision making is thus necessary for improving the efficacy of interventions promoting healthy eating.

In addition to being on the rise all over the world, studies have shown that obesity rates follow a socioeconomic gradient. More specifically, in industrialized Western societies, obesity rates have been found to be higher among people in disadvantageous social contexts (Malik et al., 2013; Loring and Robertson, 2014). Hoebel et al. (2019) report that in the years 1990–2011, obesity rates in Germany were highest among the lowest socioeconomic groups and lowest among the highest socioeconomic groups. Moreover, they found that during the examined time span, obesity incidence increased in the low socioeconomic groups (0.53 percentage points among men and 0.47 percentage points among women per year) but not in the high socioeconomic groups. Similarly, survey data from England and the United States also supports a negative correlation between socioeconomic variables (income and education levels) and obesity rates (Booth et al., 2017)^1^. This correlation has been argued to result from multiple factors including disparities in income in combination with low prices of unhealthy food, unequal healthcare access, and different levels of nutrition knowledge (McLaren, 2007; Robertson et al., 2007; Drewnowski, 2009; Harrison et al., 2010).

Importantly, being in a socially disadvantageous position often goes along with experiencing stress and negative emotions (Gallo and Matthews, 2003), which in turn can affect food intake and choice (Macht, 2008; Bublitz et al., 2010; Cardi et al., 2015; Maier et al., 2015) beyond the mentioned socioeconomic variables. More specifically, it has been shown that while there is heterogeneity in the effects of emotions on eating behavior, experiencing negative emotions generally goes along with increased intake of energy-dense and often unhealthy food (Macht, 2008; Bublitz et al., 2010; Konttinen et al., 2010). Support for these correlational findings comes from studies of social hierarchies and food consumption in animals. For instance, rodents in disadvantageous—that is, subordinate—social positions exhibit increased stress levels, altered dietary patterns, and a different fat distribution in the body. These findings have been argued to suggest a link between psychosocial stress and eating behavior that contributes to the etiology of obesity (Moles et al., 2006; Tamashiro et al., 2007; Coccurello et al., 2009). Similar effects have been found in house-hosted monkeys, with subordinate monkeys exhibiting increased levels of stress and anxiety, accompanied by elevated consumption of high-caloric foods (Wilson et al., 2008).

While these studies are informative, translating their results to human behavior has its limitations. The most obvious way in which food-related decision making differs between humans and animals is that humans can deliberate about their decisions and take higher-order objectives, like health considerations, into account. Humans can, moreover, plan—at least in developed countries—their food intake in advance. This means that for humans, one has to distinguish between at least two components of food-related decision making: food intake and food choice. By food choice we mean choice of a food item or of several items from a menu of options—which resembles, say, shopping for groceries at a supermarket. Food intake, by contrast, refers to eating behavior in a situation in which the type of food has already been decided upon—say, snacking in front of the TV.

To date, only a few experimental studies have explicitly investigated the effect of negative social contexts on eating behavior in humans. Laran and Salerno (2013) demonstrated that an “environmental harshness” priming increased the intake of high-caloric foods, probably by evoking perceptions of scarcity. This effect was attenuated when a $1 payment was given to the participants in the “harshness” condition. The findings of Laran and Salerno (2013) provide a potential explanation of the correlation between socioeconomic status and obesity reported above. Other studies have focused on the effects of lab-induced social comparisons on food intake: Cheon and Hong (2017) found that evoking comparisons with fellow citizens of higher socioeconomic status increased participants’ intake of high-caloric snacks. Sim et al. (2018) corroborated this finding and furthermore suggest that the observed effect stems from perceived deprivation relative to the better-off comparison group. Along similar lines, lab-induced disadvantageous social contexts such as social exclusion have been found to increase the intake of unhealthy (high-caloric) snacks by adults (Baumeister et al., 2005), overweight adolescents (Salvy et al., 2011), and children (Senese et al., 2020). Crucially, these studies addressed *intake* of readily available food rather than food *choice*^2^. It is conceivable, however, that negative social contexts may influence food intake and food choice to different degrees.

While social exclusion and subjective feelings of deprivation are important phenomena commonly experienced by socially disadvantaged groups, there are several other relevant dimensions of being socially disadvantaged that may also have an impact on health, such as experiencing inequality, unfairness, and inferiority (Drewnowski, 2009; Lemaitre, 2016). It remains unexplored, thus far, whether these commonly experienced disadvantageous social contexts influence food choice of healthy individuals, and whether the emotional reaction to these contexts mediates their effect on food choice.

On this background, the objective of the present study was to investigate if disadvantageous social contexts affect food choice, and if these effects are mediated by the emotions evoked by the same. In pursuit of these objectives, we conducted three experiments each including an emotion-inducing social context and a food choice task. In the first experiment we induced unfairness using the Dictator Game (DG) (Hewig et al., 2011; Strang et al., 2016), in the second experiment we induced social exclusion using the Cyberball Game (CBG) (Williams et al., 2000; Bernstein and Claypool, 2012), and in the third experiment we induced inferiority using a performance (reaction time) ranking paradigm (RTR) (Zink et al., 2008; Gong and Sanfey, 2017). We hypothesized that in line with prior correlational findings, negative social contexts would influence participants’ food choice in the direction of letting them choose tastier but unhealthier items more often, and that these effects would be mediated by emotions evoked by the respective social contexts.

## Materials and Methods

### Participants

The experiments were approved by the ethics committee of the University of Bonn, and all participants gave written informed consent according to the Declaration of Helsinki. 156 healthy participants participated in the study: 40 in Experiment 1 (DG; 22 female, 18 male; age: *M* = 25.85, *SD* = 7.67 years), 35 in Experiment 2 (CBG; 19 female, 16 male; age: *M* = 25.94, *SD* = 3.10 years)^3^, and 81 in Experiment 3 (RTR; 43 female, 38 male; age: *M* = 22.75, *SD* = 2.94 years). Given that there were no prior studies investigating the effects of disadvantageous social context on food choice, it is difficult to calculate a reasonable sample size—for instance, via power analysis—ex ante. Hence, we aimed at a number of observations that is comparable to the sample sizes reported in related studies (Baumeister et al., 2005; Salvy et al., 2011). For the first two experiments, participants were recruited via e-mail from the subject pool of the Life and Brain research center, while invitations for the third experiment were sent out via the hroot database (Bock et al., 2014) of the BonnEconLab. Registration in these databases is voluntary and open to anyone; the pools consist mostly of local university students but also include university staff and members of the general public. Participation was voluntary, and participants were paid a €10 per-hour flat fee and an additional amount of money depending on their performance and/or the experiment they completed. As a first step, participants had to fill in an online survey to ensure their eligibility for the study. Exclusion criteria were age below 18 years, Body Mass Index (BMI) below 18 or above 30 kg/m^2^, psychological and/or psychiatric disorders, eating disorders, food allergies, non-consumption of snacks, dieting, or any other medical condition known to affect eating behavior.

### Experimental Procedure

All data were collected before any analyses for the respective experiment were conducted. Below we disclose all data exclusions, all measures, and all variables acquired in the experiments.

One day before the experiment, participants were reminded to eat a snack not less than 3 h before the experiment (*M* = 4.8 h, *Median* = 3 h, *SD* = 3.7 h), so that they would be neither very hungry nor very satiated during the experiment. To check this and other baseline levels, before the experiment we asked participants to rate their subjective hunger, hours of sleep, arousal, happiness, and time of the last meal consumption. The descriptive statistics of these baseline scores, as well as the scores acquired from the psychometric measurements, are summarized in Supplementary Table 1.

All three experiments followed a similar protocol, consisting of a food rating task, a social context followed by an emotion rating stage, a food choice task, and several questionnaires. The experiments were computer-based; they were implemented using an in-house software (Scenario Designer) in Experiments 1 and 2 and z-Tree (Fischbacher, 2007) in Experiment 3. In Experiments 1 and 2, in addition to the behavioral data, we acquired functional magnetic resonance imaging (fMRI) data. That is why these two experiments were conducted while participants were alone in a room inside an MRI scanner. In Experiment 3, participants completed the tasks in silence together with 9–14 other participants. Participants used a computer of their own and were sitting in cubicles separated by room-high walls and curtains.

#### Stage 1 of All Experiments

**FIGURE 1:**
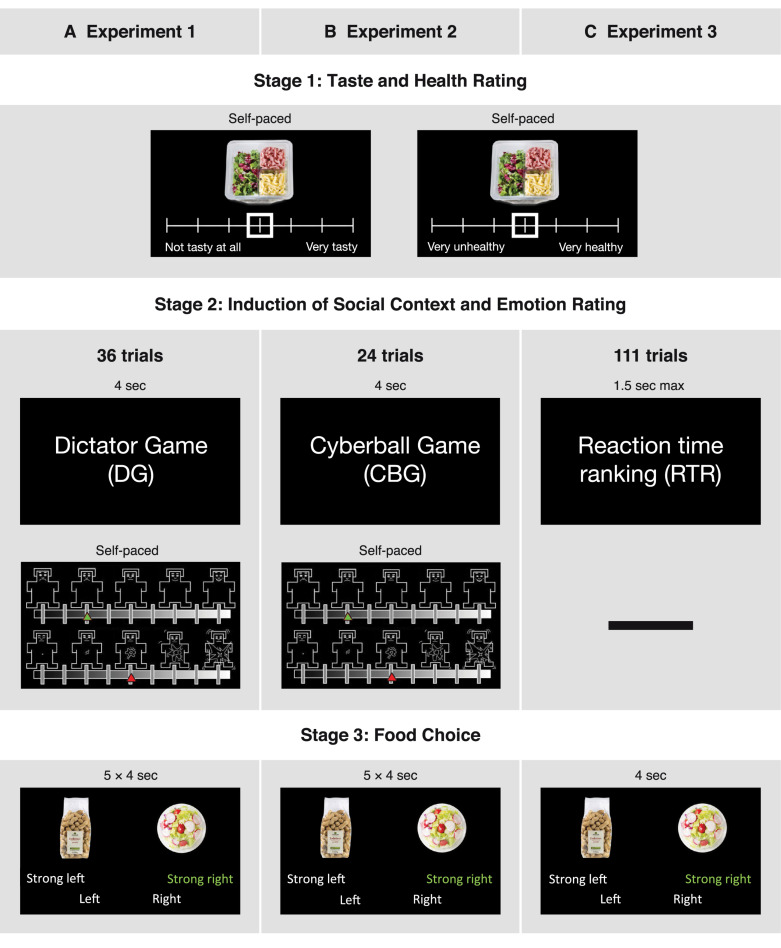
Design and time courses of the three experiments. In all three experiments, participants initially had to rate food items in terms of taste and healthiness. After this they completed one of three tasks, depending on the experiment: **(A)** being the recipient in a Dictator Game (DG), **(B)** participating in a Cyberball Game (CBG), or **(C)** participating in a reaction time ranking task (RTR). An emotion rating stage followed the DG and the CBG, but not the RTR task. Lastly, in the food choice stage, participants chose between two food items (5 choices after each game in DG and CBG; 1 choice in RTR). The sequence was repeated 36 times in the DG, 24 times in the CBG, and 111 times in the RTR task

The food rating task was adapted from a previous study (Enax et al., 2016). In this task, participants had to individually rate 158 food items in terms of healthiness and taste (see Figure 1). To acquire more sensitive ratings, in the first experiment we used an 11-point Likert scale (1—very unhealthy/not tasty at all; 11—very healthy/very tasty). Based on previous findings (Dawes, 2002; Lewis and Erdinç, 2017), to save time and simplify the use of the scales, in the second and third experiment we used a 7-point Likert scale. Taste and healthiness ratings were completed in two blocks. The order of the blocks and the order of the items to be rated within each block were randomized. The subjective ratings of healthiness and taste were used to construct food pairs for the subsequent part of the experiment.

Following Stage 1, participants had to perform multiple repetitions of Stages 2 and 3: a game giving rise to a social context (Stage 2), followed by a food choice task (Stage 3). The games used to induce social contexts differed between experiments (see Figure 1).

#### Stage 2 of Experiment 1 (DG Experiment)

In the first experiment, participants were assigned the role of recipients in a DG. We used varying monetary splits in the DG in order to subject participants to situations in which they felt treated fairly or unfairly (Strang et al., 2016). In a DG, a dictator decides on how to split an endowment between her-/ himself and a receiver, who then has to accept the dictator’s decision. Therefore, prior to the experiments, a separate session was run to acquire different money allocations decided upon by participants in the role of the dictator. These splits were then shown in our DG experiment to the participants in the role of the receiver. The stage consisted of three different money splits: unfair, neutral, and fair. In the “unfair split” condition, the participant was allocated an amount of money equal to 10, 13.3, 16.6, or 20% of the endowment, leaving the dictator with 90, 86.7, 83.4, or 80%, respectively. In the “neutral” condition, the participant was offered 30% of the endowment, while in the “fair” condition the participant was allocated 50, 46.66, 43.33, or 40% of the endowment. While the “fair” and “unfair” money splits were acquired from real people and were presented as such, the “neutral” condition consisted of one fixed value, presented to the participants as a split offered by the computer. Each condition was presented 12 times in a randomized order (thus, 3 × 12 = 36 rounds in total), and each trial lasted for 4 s. To ensure relevance of the money splits, participants were told that in the end they would get an additional amount of money based on a randomly selected round from those featuring “fair” and “unfair” splits. This means that the “neutral” condition was not relevant for the final payment, and participants knew about this. Given this knowledge, the “neutral” condition should indeed not have any influence and can thus be regarded as a baseline condition. After each money split, participants rated their emotional state in terms of valence and arousal, using the corresponding Self-Assessment Manikin subscales (Bradley and Lang, 1994). The affective space is considered to consist of these two dimensions: valence, referring to the quality (positive-negative), and arousal, referring to the intensity of the emotion (high-low) (Russell, 1979; Lang et al., 1993; Bradley and Lang, 1994; Bliss-Moreau et al., 2019). In line with previous findings, we hypothesized that unfairness in the DG as well as exclusion in the CBG would decrease valence ratings (Williams et al., 2000; Strang et al., 2016) and increase arousal ratings (Van’t Wout et al., 2006; Kelly et al., 2012). The ratings were done on a scale of 9 states, with 1 indicating very negative valence/low arousal and 9 indicating very positive valence/high arousal. The emotion ratings were self-paced (see Figure 1A). Postexperimental questions were used to check how participants felt after the different conditions.

#### Stage 2 of Experiment 2 (CBG Experiment)

In the second experiment, we used a modified version of the CBG (Weik et al., 2010; Salvy et al., 2011; Kawamoto et al., 2012) to let participants experience social inclusion and exclusion. In the CBG experiment, participants played a ball-tossing game with two other virtual players. Before the start of the game, participants were told that the game would be played online and that the other two players were real. Unbeknownst to the participants, the game was played against a computer every time, and the other players were not real. The task was modified from the original as follows: The background color was black instead of white, IDs in form of numbers were used to indicate the players rather than showing names and/or pictures, the number of conditions was fixed to two game types (inclusion, exclusion), and the order of the conditions was randomized with the restriction that one game type could not be repeated more than two times in a row. In each game, the IDs of the other players changed to avoid feelings of intentional exclusion by a particular player. Participants played the game by pressing a button with either the left or right index finger to throw the ball to the player visualized on the respective side of the computer screen. Active participation in the game was incentivized such that if participants threw the ball back 75–100% of the times the ball was thrown to them, they would earn an additional amount of €5. This procedure ensured that participants actively played the game and felt part of it. Every game consisted of 12 ball tosses and lasted around 30 s. The stage consisted of two different conditions: Participants could be either “included” (receiving 50% of the ball tosses from the other players, i.e., being in possession of the ball exactly 1/3 of the time) in or “excluded” (getting only 25% of the ball tosses, i.e., being in possession of the ball only 1/6 of the time) from the game. In total, there were 12 inclusion and 12 exclusion games. Participants rated their state valence and arousal after every game using the same procedure as in the DG experiment (see Figure 1B). Postexperimental questions were used to check whether participants perceived the degree to which they were included in the two conditions differently and whether they felt different in each condition.

#### Stage 2 of Experiment 3 (RTR Experiment)

In the last experiment, we used a performance RTR task to let participants experience being at different positions in a social hierarchy based on performance. In the RTR task, participants were instructed to engage in a real-time reaction time task, which involved pressing a button whenever a circle in the middle of the screen changed its color. The circle was presented with a random duration between 0.5 and 1.5 s, and participants had to press the button during the presentation of the circle. Responses after 1.5 s were considered a missed trial, and responses before 0.5 s were considered false starts. To check whether the variation in reaction times between and within participants could provide a natural ranking, and thus alleviate the need for deceptive feedback, we conducted a pilot study before the experiment. As expected, our pilot data indicated that participants could naturally end first, second, third, fourth, or fifth in different rounds when matched with four competitors randomly drawn from the participants of the same session. Taking this into account, the experiment was conducted with at least 10 participants per session, such that every participant’s performance could be compared to 4 other performances in real time. After each performance, the participants were shown a real ranking of themselves and 4 competitors; this way they were informed how well they performed relative to the others. False starts and missed trials were both assigned the last (5th) rank, and ties were resolved by a random draw. In total there were 111 reaction time task rounds, each followed by a ranking feedback presented for around 6 s. To make sure that recurring emotion ratings did not lead the participants to be aware or even regulate their emotions, and to keep the social context as close as possible to the food choices, in this experiment we did not acquire emotion ratings after each round (see Figure 1C). Additionally, the DG experiment showed that postexperimental valence ratings correlated strongly with the ratings acquired during the experiment (Spearman’s rank correlation DG: ρ = 0.84, *p* < 0.001). Importantly, the postexperimental valence ratings were significantly different between conditions in the same manner as the immediate valence ratings (see Supplementary Table 2). Considering all these aspects, we decided to ask participants only postexperimentally how they felt during each ranking of the RTR task. More specifically, we asked them to indicate how proud, satisfied, annoyed, frustrated, and disappointed they felt after being ranked 1st, 3rd, and 5th. In line with previous findings, we hypothesized that inferiority experienced in the RTR would increase negative emotion ratings and decrease positive emotion ratings (Zink et al., 2008; Luo et al., 2018). Similar to the CBG experiment, we used postexperimental questions to assess whether participants perceived the ranks differently.

#### Stage 3 of All Experiments

The third stage of all three experiments was a food choice task. Each food choice trial was a four-alternative forced choice, and participants were asked to choose the food item that they preferred to eat at that very moment. However, the degree to which participants were prompted to consider healthiness during their choices differed across studies: In the DG and the CBG experiment, participants were prompted to consider healthiness while making their choices, whereas this cue was absent in the RTR experiment. (For the exact instructions provided to the participants see the Supplementary Material.) Participants had the opportunity to express the strength of each choice such that that one food item could be “preferred” or “strongly preferred” over the other food item (see Figure 1). One of all choices, from a randomly selected round was implemented at the end of each experiment; which choice would be implemented varied between participants was unknown to them so that they would treat each choice as equally important. The food pairs used in this phase were constructed based on the subjective ratings completed before the social context and emotion rating stage. Based on these previous ratings, the food choice trials were divided into congruent and incongruent trials. In congruent trials, health and taste aspects of the foods were aligned, with the healthier item being also tastier. In incongruent trials, health and taste attributes were not aligned, with the less healthy item being tastier than the other. Thus, by choosing the healthier item in the incongruent trials participants automatically forwent the tastier product and vice versa. The congruent trials were added as a sanity check to evaluate whether participants made reasonable decisions, that is, decisions that were aligned with their earlier health and taste ratings. The ratio of these trials (incongruent : congruent) was 3 : 2 in the DG, 4 : 1 in the CBG, and 10 : 1 in the RTR experiment. Each food pair was shown for 4 s, and the pairs were presented in random order. In the DG and in the CBG task, five food choice trials were presented after each emotion rating (see Figures 1A,B). In the RTR experiment, one food choice was presented after each ranking (see Figure 1C). Trials were counterbalanced across conditions in all three experiments.

#### Postexperimental Questionnaires

Finally, to control for the effect of possible differences in eating styles (van Strien et al., 2013; Blechert et al., 2014), and dispositional self-control (Hare et al., 2009; Stutzer and Meier, 2016), after each of the three experiments participants completed the following psychometric questionnaires: the Brief Self-Control Scale (BSCS; German: SCS-K-D) (Tangney et al., 2004; Bertrams and Dickhäuser, 2009), Dutch Eating Behavior Questionnaire (DEBQ) (van Strien et al., 1986; Grunert, 1989), Three Factor Eating Questionnaire (TFEQ; German: Fragebogen zum Essverhalten—FEV) (Stunkard and Messick, 1985; Pudel and Westenhöfer, 1989), and several questions designed to assess manipulation efficacy in every experiment. In the CBG experiment, we asked additional questions to assess the ostracism effect, as suggested in the literature (Williams et al., 2000). After the questionnaires were completed, participants were debriefed and reimbursed. In the CBG experiment, as part of the debriefing procedure, participants were told that the other players in the game were not real.

In addition to these measures, in the DG and CBG experiments, fMRI data were collected but are not reported in the current paper. Similarly, several additional questionnaires were included in the different experiments but are not reported in the current paper^4^. The questionnaires differed between the experiments because we had different analyses of subgroups in mind. In the current paper, we focus on possible subgroup effects present in the combined data from all the three experiments and hence only report results for the data that were collected in all of the experiments.

### Statistical Analysis

#### General Information

Statistical analysis was performed using the R language (R Core Team, 2019). The following packages were used: *readxl, psych, dplyr, ggplot2, reshape2, lme4, lmerTest, MuMIn, sjstats, multcomp, mediation*, and *TOSTER*. A sensitivity power analysis was conducted using the G^∗^Power software package (version 3.1.9.3) (Faul et al., 2009).

#### Assessing the Manipulation Efficacy

To check whether different social contexts lead to changes in the emotion ratings, we estimated linear mixed-effects models with emotion ratings as the dependent and condition as the explanatory variables. For the DG and CBG experiments, we estimated one model with valence and one with arousal ratings as the dependent variable (Eqs 1.1 and 1.2). For the RTR, we estimated one model with mean positive (sum of proud and satisfied ratings divided by two) and one with mean negative emotion ratings (sum of annoyed, frustrated, and disappointed ratings divided by three) as the dependent variable (Eqs 1.3 and 1.4):

(1.1) $$V\,a\,l\,e\,n\,c\,e\,r\,a\,t\,i\,n\,g\,s_{i\,j}=\beta_{0}+\beta_{1}\,C\,o\,n\,d\,i\,t\,i\,o\,n_{i\,j}+u_{j}+\varepsilon_{i\,j};$$

(1.2) $$A\,r\,o\,u\,s\,a\,l\,r\,a\,t\,i\,n\,g\,s_{i\,j}=\beta_{0}+\beta_{1}\,C\,o\,n\,d\,i\,t\,i\,o\,n_{i\,j}+u_{j}+\varepsilon_{i\,j};M\,e\,a\,n\,p\,o\,s\,i\,t\,i\,v\,e\,e\,m\,o\,t\,i\,o\,n\,r\,a\,t\,i\,n\,g\,s_{i\,j}=\beta_{0}+\beta_{1}\,C\,o\,n\,d\,i\,t\,i\,o\,n_{i\,j}+u_{j}+\varepsilon_{i\,j};M\,e\,a\,n\,n\,e\,g\,a\,t\,i\,v\,e\,e\,m\,o\,t\,i\,o\,n\,r\,a\,t\,i\,n\,g\,s_{i\,j}=$$

(1.3) $$M\,e\,a\,n\,p\,o\,s\,i\,t\,i\,v\,e\,e\,m\,o\,t\,i\,o\,n\,r\,a\,t\,i\,n\,g\,s_{i\,j}=\beta_{0}+\beta_{1}\,C\,o\,n\,d\,i\,t\,i\,o\,n_{i\,j}+u_{j}+\varepsilon_{i\,j};$$

(1.4) $$M\,e\,a\,n\,n\,e\,g\,a\,t\,i\,v\,e\,e\,m\,o\,t\,i\,o\,n\,r\,a\,t\,i\,n\,g\,s_{i\,j}=\beta_{0}+\beta_{1}\,C\,o\,n\,d\,i\,t\,i\,o\,n_{i\,j}+u_{j}+\varepsilon_{i\,j}.$$

The subscript *j* indexes participants, while *i* indexes observations per subject. That is, *u*_*j*_ is a subject-specific random intercept, and *ε_*ij*_* is the residual. An observation corresponds to one emotion rating. Condition is a factor (categorical) variable. For the DG, *Condition*_*ij*_ had three levels, indicating whether the monetary split announced to subject *j* in trial *i* was fair, neutral, or unfair. For the CBG, *Condition*_*ij*_ had two levels, indicating whether subject *j* was included or excluded in trial *i*. For the RTR, *Condition*_*ij*_ had five levels, reflecting the rank that subject *j* attained in trial *i*.

Additionally, to check whether the participants perceived accurately that there were different conditions in the experiments, we performed paired-sample *t*-tests on the questions asked postexperimentally (CBG: how many times they got the ball in each condition; RTR: how many times they were ranked 1st and 5th). In the CBG experiment, to additionally assess the ostracism effect we performed mixed-effects linear regression analyses on the postexperimentally asked questions.

#### Assessing the Suitability of the Food Choice Task

The congruent trials served two purposes: First, we used them to check whether participants’ food choices were reasonable. To do so, we conducted one-sample *t*-tests (for all three datasets separately) and compared the percentage of tastier–healthier choices in the congruent trials to chance level (50%). Second, we used the congruent trials to check for fatigue effects. To do so, we regressed reaction times (RT) on the trial number using a mixed-effects linear regression analysis with residual *ε_*ij*_* ∼ *N*(0, *σ_ε_*^2^) and subject-specific random effects *u*_*j*_ ∼ *N*(0, *σ_*u*_*^2^) (see Eq. 2):

(2) $$\log(RT_{ij})={\text{β}}_{0}+{\text{β}}_{1}\;Trial\;number_{ij}+u_{j}+{\text{ε}}_{ij}.$$

Here, similar to the previous model, the subscript *j* indexes participants, while *i* indexes observations per subject. RTs were log-transformed due to their skewed distribution.

Similarly, to investigate whether our food choice task worked as intended and that the food choices were reasonable in the incongruent trials as well, we checked the impact of taste and health ratings on food decisions. To achieve this, we performed a mixed-effects logistic regression; that is, the error term is assumed to follow the standard logistic distribution, *ε_*ij*_* ∼ *L*(0, 1), and the subject-specific random effects are *u*_*j*_ ∼ *N*(0, *σ_*u*_*^2^) (see Eq. 3). In this model, the choice of the item on the left side (*Chose left*: 1 = Yes, 0 = No) was entered as the dependent variable, the *z*-scored difference in taste (*TD*) and health ratings (*HD*) between the simultaneously presented items (Left – Right) were entered as explanatory variables, and the random intercept term was added to account for between-subject heterogeneity:

$$\begin{array}{c} Chose\;left_{ij}=1\;if\;{\text{β}}_{0}+{\text{β}}_{1}TD_{ij}+{\text{β}}_{2}HD_{ij}+u_{j}\\ +\varepsilon_{ij}>0,\text{ and }Chose\;left_{ij}=0\;otherwise \end{array}$$

This gives rise to the regression equation:

(3) $$Chose\;left_{ij}=F({\text{β}}_{0}+{\text{β}}_{1}TD_{ij}+{\text{β}}_{2}HD_{ij}+u_{j})$$

where *F*(*x*) = 1 / [1 + exp(−*x*)] is the cumulative distribution function of the standard logistic distribution. This model was estimated for all three datasets.

#### Effect of Social Contexts on Food Choice

To assess the effect of social context on food choice, we analyzed the proportion of tastier food choices in the incongruent trials and tested whether the different conditions influenced this proportion systematically. To this end, we estimated mixed-effects models for the three datasets separately as a first step. In a second step, we aimed to further investigate whether the probability of choosing the tastier item in the incongruent trials varied with the condition and pooled the data of the three experiments so that we could analyze them jointly.

For the separate data analyses, we used mixed-effects logistic regression models with *ε_*ij*_* ∼ *L*(0, 1) and *u*_*j*_ ∼ *N*(0, *σ_*u*_*^2^). In these models, the dependent variable was a binary variable indicating whether participants chose the tastier item in the trial (*Chose tastier*: 1 = Yes, 0 = No), and the explanatory variable was condition. Subject-specific random intercepts were added to account for between-subject heterogeneity:

(4) $$Chose\;tastier_{ij}=F({\text{β}}_{0}+{\text{β}}_{1}Condition_{ij}+u_{j}).$$

For the combined data analysis, we pooled the three datasets. Recall that *Condition*_*ij*_ is a factor variable which originally has three levels in the DG, two levels in the CBG, and five levels in the RTR. To have the same number of levels for all three datasets, we discarded the neutral level from the DG and the 3rd-rank level from the RTR. Moreover, since in the RTR we had more levels, we pooled 2nd with 1st rank and 4th with 5th rank. This way we had two levels across all three experiments, one indicating a positive social context (fair, inclusion, 1st/2nd rank) and one indicating a negative social context (unfair, exclusion, 4th/5th rank). The food choice data remained the same.

We analyzed the combined data in two ways. First, we calculated the difference in the mean relative frequencies of tastier food choices between the two conditions (negative and positive social context) and analyzed these differences with a one-sample *t*-test. With this analysis we aimed to assess whether the difference between our conditions is significant in the most straightforward way.

Second, similar to the separate data analyses, we ran a mixed-effects logistic regression with *Chose tastier* as the dependent variable, condition and experiment as the explanatory variables, and a subject-specific intercept:

(5) $$Chose\;tastier_{ij}=F({\text{β}}_{0}+{\text{β}}_{1}Condition_{ij}+{\text{β}}_{2}Experiment_{j}+u_{j}).$$

In this regression, we added a factor variable indicating the experiment (DG, CBG, RTR). We did so to capture potential differences between the three experiments resulting from the use of different subject pools, different locations, and different wording of the instructions. With this analysis we aimed to assess whether the difference between our conditions is significant if we control for the type of the manipulation (i.e., the *Experiment* variable) and are thereby able to explain additional variance.

Finally, we performed a sensitivity power analysis to assess the minimum effect size that could be detected in our most powerful analysis, that is, in the combined data analysis. For this, we used the one-sample *t*-test approach implemented in G^∗^Power. We also performed an equivalence test by using the Two One-Sided Tests (TOST) procedure implemented in the *TOSTER* package in R (Lakens et al., 2018). With this analysis we assess whether the difference between food choices in the negative and positive social contexts is statistically equivalent to 0.

#### Mediation Analyses

To assess whether emotions mediate the association between social context and food choice, we ran mediation analyses on all three datasets. In all three experiments we had two measures of emotions: valence and arousal in DG and CBG, and positive and negative emotion ratings in the RTR. Thus, for each dataset, we ran two separate mediation analyses, each with one of the self-reported emotions as mediators.

**FIGURE 2:**
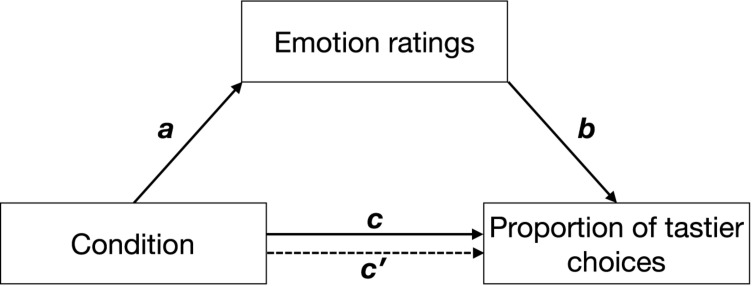
Hypothetical mediation. Path *c* indicates the effect of condition (positive social context, negative social context) on the proportion of tastier food choices, path *a* indicates the effect of condition on emotions, path *b* indicates the effect of emotions on the proportion of tastier food choices when controlling for condition, and path *c*′ indicates the effect of condition on the proportion of tastier food choices when considering emotions as mediators. Arrows indicate the direction of the hypothesized causal effects.

We used a model-based causal mediation analysis (see Figure 2) as implemented in the *mediation* package for R (Tingley et al., 2014). Path *c* was estimated by regressing the proportion of tastier choices on condition (Eq. 6.1). Path *a* was estimated by regressing emotion ratings on condition (Eq. 6.2), and paths *b* and *c*′ were estimated by regressing the proportion of tastier food choices on condition and *z*-scored emotion ratings (see Eq. 6.3). Given that in all our experiments, social context was experimentally manipulated and was followed by emotion ratings and food choice, we assume the paths in our mediation analyses to be causal and one-directional (with the direction indicated by the arrows in Figure 2). Direct, mediation, and total effects were estimated using a quasi-Bayesian Monte Carlo simulation method (number of simulations = 1,000) based on normal approximation:

(6.1) $$Proportion\;of\;tastier\;choices_{ij}={\text{β}}_{0}+cCondition_{ij}+u_{j}+{\text{ε}}_{ij};$$

(6.2) $$Emotion\;ratings_{ij}={\text{β}}_{0}+a\;Condition_{ij}+u_{j}+{\text{ε}}_{ij};$$

(6.3) $$Proportion\;of\;tastier\;choices_{ij}={\text{β}}_{0}+b\;Emotion\;ratings_{ij}+c^{\prime }Condition_{ij}+u_{j}+{\text{ε}}_{ij}.$$

For the DG and the CBG experiments, every social context condition was followed by emotion ratings, which were followed by 3 and 4 incongruent food choice trials, respectively. That is, for the DG we had 36 emotion ratings and around 108 food choices, and for the CBG we had 24 emotion ratings and 96 food choices per participant. Because of this, for these datasets, we calculated the proportion of tastier choices for every emotion rating trial. By contrast, in the RTR experiment, emotion ratings were collected after the experiment, and only for the 1st, 3rd, and 5th attained rank. Hence, for the mediation analyses, we excluded the trials were participants were ranked 2nd or 4th. Therefore, *Condition* now is a factor variable with three levels in the DG, two levels in the CBG, and three levels in the RTR. Given that we had only one emotion rating per level of the factor variable *Condition*, for this dataset we calculated the proportion of tastier choices per level. This means that in the mediation analysis, an observation *i* does not correspond to a single food choice trial anymore but includes all trials covered by an emotion rating question.

#### Subgroup Effects

To evaluate whether condition has a different effect on the proportion of tastier choices in different subgroups, identified via questionnaire scores, we estimated interaction models with proportion of tastier choices as the dependent variable, condition, *z*-scored questionnaire scores (BSCS score, TFEQ subscale scores, DEBQ subscale scores), interaction between condition and questionnaire scores, and experiment as explanatory variables. Similar to the previous models, we added a random intercept *u*_*j*_ to account for between-subject effects (see Eq. 7). This model was calculated for each questionnaire score separately:

(7) $$\begin{array}{c} Proportion\;of\;tastier\;choices_{ij}={\text{β}}_{0}+{\text{β}}_{1}Condition_{ij}\\ +{\text{β}}_{2}Questionnaire\;score_{j}+{\text{β}}_{3}Questionnaire\;score_{j}\\ \times Condition_{ij}+{\text{β}}_{4}Experiment_{j}+u_{j}+{\text{ε}}_{ij} \end{array}$$

To have the same number of levels for all three datasets, we proceeded as described in the “Effect of Social Contexts on Food Choice” subsection above and discarded the neutral level from the DG and the median-rank level from the RTR. Moreover, since in the RTR we had a total of five levels, we pooled 2nd with 1st rank and 4th with 5th rank. This results in two levels across all three experiments, one indicating a positive social context (fair, inclusion, 1st/2nd rank) and one indicating a negative social context (unfair, exclusion, 4th/5th rank). With the dependent variable being the proportion of tastier food choices by subject *j* in condition *i*, we have two observations per subject, *i* ∈ {1, 2}, for this joint analysis of all three experiments. (It would be equally valid to treat each food choice trial as an independent observation and use *Chose tastier* as the dependent variable, as described above in the “Effect of Social Contexts on Food Choice” subsection. It turns out that our qualitative results do not depend on the specification of the regression.)

## Results

### Overview

In this section, we first show the effect of social contexts on emotion ratings. Second, we show that participants made reasonable and systematic food choices in the congruent and incongruent trials. Third, we report the effect of social contexts on food choice. In addition to the main effect, we also report the effect of emotions as mediators between social context and the proportion of tastier food choices in the incongruent trials. Finally, we report subgroup effects of negative social contexts relative to positive social contexts on the proportion of tastier food choices.

### Assessing the Manipulation Efficacy

Linear mixed-effects models indicated that in the DG experiment condition had a significant effect on the valence [*χ^2^*_(__2)_ = 712.77, *p* < 0.001, marginal *R*^2^ = 0.26] and arousal ratings [*χ^2^*_(__2)_ = 93.95, *p* < 0.001, marginal *R*^2^ = 0.03]. Tukey-adjusted comparisons revealed that valence ratings in the unfair condition were significantly lower than in the neutral and fair condition (Neutral − Unfair: β = 1.11, *SE* = 0.07, 95% CI [0.94, 1.28], *z* = 15.18, *p* < 0.001; Fair − Unfair: β = 2.23, *SE* = 0.07, 95% CI [2.06, 2.41], *z* = 30.46, *p* < 0.001; Fair − Neutral: β = 1.12, *SE* = 0.07, 95% CI [0.95, 1.29], *z* = 15.29, *p* < 0.001). In terms of arousal, the ratings were significantly different between the neutral and fair, and unfair and fair conditions (Neutral − Unfair: β = −0.64, *SE* = 0.07, 95% CI [−0.81, −0.48], *z* = −9.07, *p* < 0.001; Fair − Unfair: β = −0.56, *SE* = 0.07, 95% CI [−0.72, −0.39], *z* = −7.86, *p* < 0.001; Fair − Neutral: β = 0.09, *SE* = 0.07, 95% CI [−0.08, 0.25], *z* = 1.21, *p* = 0.45).

In the CBG experiment, valence ratings were significantly higher in the inclusion than in the exclusion condition [*χ^2^*_(__1)_ = 16.81, *p* < 0.001, marginal *R*^2^ = 0.009; Inclusion − Exclusion: β = 0.33, *SE* = 0.08, 95% CI [0.17, 0.49], *z* = 4.12, *p* < 0.001], while arousal ratings were not significantly different between the conditions [*χ^2^*_(__1)_ = 1.25, *p* = 0.26, marginal *R*^2^ = 0.0005; Inclusion − Exclusion: β = −0.09, *SE* = 0.08, 95% CI [−0.24, 0.06], *z* = −1.12, *p* = 0.26].

In the RTR experiment, condition had a significant effect on the positive [*χ^2^*_(__2)_ = 265.61, *p* < 0.001, marginal *R*^2^ = 0.65] and negative emotion ratings [*χ^2^*_(__2)_ = 133.79, *p* < 0.001, marginal *R*^2^ = 0.37]. Tukey-adjusted comparisons revealed that all pairwise comparisons were significant (Positive emotions: 3rd − 5th: β = 1.76, *SE* = 0.21, 95% CI [1.27, 2.25], *z* = 8.45, *p* < 0.001; 1st − 5th: β = 5.01, *SE* = 0.21, 95% CI [4.52, 5.50], *z* = 23.90, *p* < 0.001; 1st − 3rd: β = 3.25, *SE* = 0.21, 95% CI [2.76, 3.74], *z* = 15.51, *p* < 0.001; Negative emotions: 3rd − 5th: β = −1.19, *SE* = 0.23, 95% CI [−1.73, −0.64], *z* = −5.12, *p* < 0.001; 1st − 5th: β = −3.32, *SE* = 0.23, 95% CI [−3.87, −2.77], *z* = −14.18, *p* < 0.001; 1st − 3rd: β = −2.13, *SE* = 0.23, 95% CI [−2.68, −1.58], *z* = −9.10, *p* < 0.001). For an illustration of these effects (see Supplementary Figure 1).

Additional postexperimental questions indicated that in the CBG experiment, on average, participants thought that they got the ball around 42.69% in the inclusion condition and 32.4% in the exclusion condition. This difference was statistically significant [*t*_(__34)_ = 2.11, *p* = 0.04, 95% CI [0.40, 20.18]], and the average stated frequencies are close to the actual frequencies (50% and 25%, respectively). Mixed-effects linear regressions on the postexperimentally asked questions indicate that in the exclusion condition, participants felt more ignored [*χ^2^*_(__1)_ = 27.00, *p* < 0.001, marginal *R*^2^ = 0.32], less wanted [*χ^2^*_(__1)_ = 24.03, *p* < 0.001, marginal *R*^2^ = 0.24], less invincible [*χ*^2^_(__1)_ = 6.30, *p* = 0.01, marginal *R*^2^ = 0.05] and less powerful [*χ*^2^_(__1)_ = 12.33, *p* < 0.001, marginal *R*^2^ = 0.11] than in the inclusion condition.

Similarly, postexperimental questions in the RTR experiment indicate that, on average, participants felt that they attained the first rank around 16.8% and the last rank around 15.8% of all rounds. Given that the average frequency of each attained rank is 20% by construction, participants seem to have been similarly reluctant to report having performed very well or very badly. Indeed, when testing whether the perceived frequency deviates from the actual frequency for the first and last rank, we find that it significantly does [1st rank: *t*_(__80)_ = 3.23, *p* = 0.002, 95% CI [1.38, 5.78]; 5th rank: *t*_(__80)_ = 5.06, *p* < 0.001, 95% CI [4.46, 10.23]]. However, the frequency for the last rank was not significantly different from the frequency for the first rank [*t*_(__80)_ = 0.31, *p* = 0.76, 95% CI [−5.45, 7.45]], suggesting that participants were not underconfident or overconfident regarding their performance on average.

### Assessing the Suitability of the Food Choice Task

#### Consistency of Food Choices in the Congruent Trials

In the congruent trials in all three experiments, participants chose the healthier food item—which in these trials also was at least as tasty as the other food item—significantly more often than chance level (50%). In all three experiments, the mean share is above 80% [DG: *M* = 87.7% of the congruent trials, *SD* = 7.6%, *t*_(__39)_ = 31.4, *p* < 0.001, 95% CI [85.31, 90.17]; CBG: *M* = 84.1% of the congruent trials, *SD* = 11.14%, *t*_(__34)_ = 18.1, *p* < 0.001, 95% CI [80.22, 87.87]; RTR: *M* = 80.0% of the congruent trials, *SD* = 13.69%, *t*_(__80)_ = 19.72, *p* < 0.001, 95% CI [76.97, 83.03]].

Mixed-effects linear regression analysis indicates that there were no fatigue effects (see Eq. 2), as time (trial number) had a significant effect on the RT such that the further an experimental session progressed, the shorter the reaction times became [DG: β = −0.001, *SE* = 0.0001, *t*_(2__816__)_ = −13.5, *p* < 0.001, 95% CI [−0.002, −0.001]; CBG: β = −0.002, *SE* = 0.0003, *t*_(__789)_ = −9.51, *p* < 0.001, 95% CI [−0.003, −0.002]; RTR: β = −0.001, *SE* = 0.0002, *t*_(__721)_ = −5.22, *p* < 0.001, 95% CI [−0.002, −0.001]].

#### Influence of Taste and Healthiness Ratings on Food Choices in the Incongruent Trials

**FIGURE 3:**
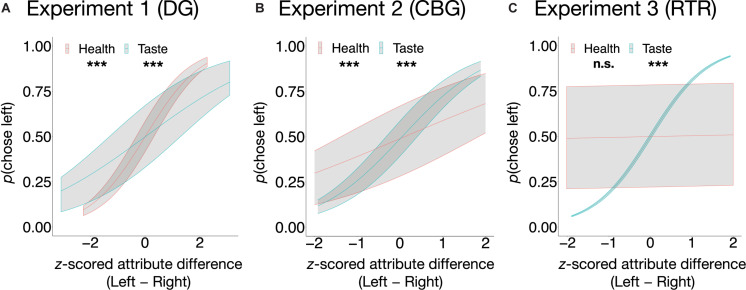
Average predicted probabilities of food choice as a function of taste and health in the incongruent trials in the DG **(A)**, the CBG **(B)**, and the RTR **(C)**. In the DG experiment **(A)** and in the CBG experiment **(B)**, both taste and health were significant predictors of choice, whereas in the RTR experiment **(C)** only taste was. Predicted probabilities are shown with upper (75%) and lower (25%) quartiles. ***p < 0.001, n.s., not significant.

As expected, in the incongruent trials of all experiments, taste significantly explained variation in choices (see Eq. 3) such that the tastier one item was in comparison to the other item, the higher was the probability of it being chosen (DG: β = 0.53, *SE* = 0.05, *z* = 11.5, *p* < 0.001, *OR* = 1.70, 95% CI [1.55, 1.86]; CBG: β = 1.08, *SE* = 0.06, *z* = 16.88, *p* < 0.001, *OR* = 2.96, 95% CI [2.61, 3.35]; RTR: β = 1.45, *SE* = 0.05, *z* = 27.93, *p* < 0.001, *OR* = 4.27, 95% CI [3.85, 4.73]). Similarly, in all three experiments, healthiness was positively related to food choice (see Eq. 3). Its impact, however, was significant only in the DG (β = 0.98, *SE* = 0.05, *z* = 19.37, *p* < 0.001, *OR* = 2.66, 95% CI [2.41, 2.94]) and in the CBG (β = 0.51, *SE* = 0.06, *z* = 8.85, *p* < 0.001, *OR* = 1.67, 95% CI [1.49, 1.87]), but not in the RTR experiment (β = 0.03, *SE* = 0.04, *z* = 0.66, *p* = 0.51, *OR* = 1.03, 95% CI [0.95, 1.12]). The relation between the probability of choosing left in the incongruent trials and attribute difference (Left − Right) between the food pairs is depicted in Figure 3.

### Effect of Social Context on Food Choice

#### Separate Analyses of the Three Experiments

In the DG experiment, in line with the given instructions, in the incongruent trials participants chose the healthier item more often (*M* = 59.63% of the trials, *SD* = 23.92%) than the tastier item (*M* = 38.89% of trials, *SD* = 23.71%) (missed trials: *M* = 1.48% of the trials, *SD* = 2.49%). Without such an instruction, in the CBG and the RTR experiments, participants chose the tastier item more often (CBG: *M* = 60.80% of trials, *SD* = 20.75%; RTR: *M* = 75.51% of the trials, *SD* = 14.28%) than the healthier item (CBG: *M* = 37.44% of the trials, *SD* = 20.45; RTR: *M* = 23.52% of the trials, *SD* = 14.43%) (missed trials: CBG: 1.76% of the trials, *SD* = 3.61%; RTR: *M* = 0.98% of the trials, *SD* = 1.23%).

**FIGURE 4:**
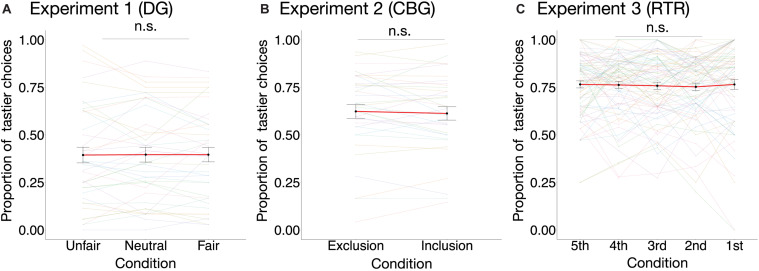
Proportion of tastier choices per condition in the three experiments. The proportion of tastier choices was not significantly different between conditions in **(A)** the DG experiment [$\chi_{\left(2\right)}^{2}=0.02$, *p* = 0.99, marginal *R*^2^ = 0.00002; *n* = 40], **(B)** the CBG experiment [$\chi_{\left(1\right)}^{2}=0.53$, *p* = 0.47, marginal *R*^2^ = 0.001; *n* = 35], or **(C)** the RTR experiment [$\chi_{\left(4\right)}^{2}=0.81$, *p* = 0.94, marginal *R*^2^ = 0.001; *n* = 81]. Colored dots are individual data points, and the black dots are mean values across participants. For better visualization of the differences between conditions, individual observations are connected with color-coded lines, whereas the mean values are connected with a red line. Error bars represent the standard error of the mean. Effects are estimated using mixed-effects linear models. n.s., not significant.

In none of the three experiments did condition have an effect on the proportion of tastier choices [DG: *χ^2^*_(__2)_ = 0.02, marginal *R*^2^ = 0.00002, *p* = 0.99; CBG: *χ^2^*_(__1)_ = 0.53, *p* = 0.47, marginal *R*^2^ = 0.001; RTR: *χ^2^*_(__4)_ = 0.81, marginal *R*^2^ = 0.001, *p* = 0.94] (see Figure 4). Similarly, mixed-effects logistic regression models estimated for the three datasets separately (see Eq. 4) indicated that condition could not significantly explain variance in choosing the tastier item (see Table 1).

**TABLE 1:** Mixed-effects logistic regression results with choosing the tastier item as the dependent variable. In all three experiments, condition did not significantly explain variance in choosing the tastier food item.

Fixed effects	Estimate (*SE*)	*p*-value	*OR*	CI (95%)
**DG: Chose tastier (1 = Yes, 0 = No)**				
Intercept	−0.58(0.2)	0.005	0.56	[0.37, 0.84]
Unfair vs. Neutral	0.01 (0.1)	0.91	1.01	[0.85, 1.20]
Unfair vs. Fair	0.01 (0.1)	0.91	1.01	[0.85, 1.20]

**Random effects**	**$\sigma_{u}^{2}$**	***SD***		

Intercept (Subject ID)	3.29	1.24		

**Model**				

Marginal *R*^2^/Conditional *R*^2^	0.000/0.320			

**Fixed effects**	**Estimate (*SE*)**	***p*-value**	***OR***	**CI (95%)**

**CBG: Chose tastier (1 = Yes, 0 = No)**				
Intercept	0.58 (0.18)	0.001	1.78	[1.26, 2.52]
Exclusion vs. Inclusion	−0.05(0.08)	0.50	0.95	[0.81, 1.11]

**Random effects**	**$\sigma_{u}^{2}$**	***SD***		

Intercept (Subject ID)	3.29	0.99		

**Model**				

Marginal *R*^2^/Conditional *R*^2^	0.000/0.230			

**Fixed effects**	**Estimate (*SE*)**	***p*-value**	***OR***	**CI (95%)**

**RTR: Chose tastier (1 = Yes, 0 = No)**				
Intercept	1.32 (0.11)	<0.001	3.74	[3.02, 4.63]
Rank 5 vs. Rank 4	0.01 (0.1)	0.93	1.01	[0.85, 1.19]
Rank 5 vs. Rank 3	−0.02(0.1)	0.79	0.98	[0.82, 1.16]
Rank 5 vs. Rank 2	−0.07(0.1)	0.42	0.93	[0.78, 1.11]
Rank 5 vs. Rank 1	0.10 (0.1)	0.29	1.11	[0.92, 1.34]

**Random effects**	**$\sigma_{u}^{2}$**	***SD***		

Intercept (Subject ID)	3.29	0.80		
**Model**				
Marginal *R*^2^/Conditional *R*^2^	0.001/0.163			

**n = 40 for the DG, n = 35 for the CBG, and n = 81 for the RTR. SE, Standard error; OR, Odds ratio; CI, Confidence interval. CIs are shown for ORs.**

#### Analysis of the Combined Data Set

When analyzing the data sets of all three experiments jointly, a one-sample *t*-test indicated that the difference in the mean frequencies of choosing the tastier item between the positive (*M* = 0.635, *SD* = 0.25) and the negative (*M* = 0.636, *SD* = 0.25) condition was not significantly different from 0 [β = −0.0004, *t*_(__155)_ = −0.046, *p* = 0.96, 95% CI: [−0.017, 0.016]]. Similarly, mixed-effects logistic regression on the three data sets combined (see Eq. 5) also indicated that condition had no significant effect (β = −0.02, *p* = 0.65) on the probability of choosing the tastier item (Positive condition: *M* = 0.6416, *SD* = 0.479; Negative condition: *M* = 0.642, *SD* = 0.479; see Table 2 and Supplementary Figure 2).

**TABLE 2:** Mixed-effects logistic regression results with condition and experiment as explanatory and choosing the tastier item as the dependent variable.

Fixed effects	Estimate (*SE*)	*p-*value	*OR*	CI (95%)
**Combined data: Choosing the tastier item (1 = Yes, 0 = No)**				
(Intercept)	0.56 (0.17)	0.001	1.75	[1.25, 2.45]
Condition	−0.02(0.04)	0.65	0.98	[0.90, 1.07]
CBG vs. DG	−1.10(0.23)	<0.001	0.33	[0.21, 0.52]
CBG vs. RTR	0.79 (0.20)	<0.001	2.20	[1.47, 3.28]

**Random effects**	**$\sigma_{u}^{2}$**	***SD***		

Subject ID (Intercept)	3.29	0.97		
**Model**				
Marginal *R*^2^/Conditional *R*^2^	0.118/0.316			

**Three data sets were combined (n = 156). Even when controlling for the type of experiment, condition had no effect on the probability of choosing the tastier item. n = 156. SE, Standard error; OR, Odds ratio; CI, Confidence interval. CIs are shown for ORs. Condition: 0 = Negative, 1 = Positive.**

The sensitivity power analysis revealed that we have 80% power to detect an effect not smaller than Cohen’s *d* = 0.2257 at a *p*-value of 0.05. This suggests that our design (with the combined data) is sensitive enough to capture a small effect if present. In other words, with our level of noise in the data (the *SD* of the differences in the mean frequencies of tastier choices between the two conditions is 0.1030), we would have been able to detect a 2.325% change (*d* × *SD* = 0.2257 × 0.1030 = 0.02325) between the conditions with 80% probability at α = 0.05.

The equivalence test using TOST was significant on the 5% level, given equivalence bounds of Cohen’s *d* = ± 0.14 [*t*_(__155)_ = 1.703, *p* = 0.0453, 90% CI [−0.014, 0.013]].

#### Mediation Analyses

**FIGURE 5:**
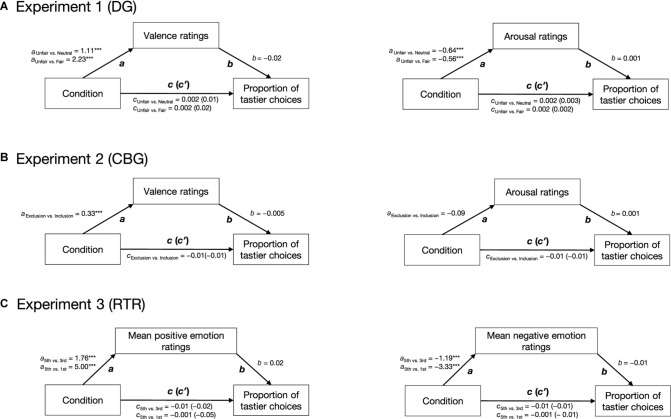
Results of the mediation analyses. For all three experiments, two mediation analyses were run separately: for the DG **(A)** and CBG **(B)**, one with valence and one with arousal as mediators; for the RTR **(C)**, one with mean positive and one with mean negative emotions as mediators. In all experiments, condition had a significant effect on self-reported emotions (path *a*). Emotions did not have an effect on the proportion of tastier food choices (path *b*). Condition did not have an effect on the proportion of tastier food choices (path *c*) neither when including self-reported emotions as mediators (path *c*′). Arrows indicate the assumed direction of the effects. ***p < 0.001.

The results of the mediation analyses are reported in Figure 5. Overall our analyses indicated that while condition had a significant effect on self-reported emotions, the latter did not have a significant effect on the proportion of tastier choices. The direct, mediation, and total effects were not significant (see Supplementary Table 3).

#### Subgroup Effects

Interaction models (see Eq. 7) indicated that condition did not have a significant effect on the proportion of tastier choices in different subgroups (see Supplementary Table 3). We found that dispositional self-control as measured via the BSCS score [β = −0.06, *SE* = 0.02, *t*_(__174.6)_ = −4.11, *p* < 0.001 [Bonferroni-corrected *p* < 0.001], 95% CI [−0.09, −0.03]] was related significantly to the proportion of tastier food choices. Cognitive Control score of the TFEQ [β = −0.04, *SE* = 0.02, *t*_(__174.03)_ = −2.4, *p* = 0.02, 95% CI [−0.07, −0.01]], and the External Eating score of the DEBQ [β = 0.04, *SE* = 0.02, *t*_(__173.5)_ = 2.75, *p* = 0.007, 95% CI [0.01, 0.08]] were also related to the proportion of tastier choices, however, these scores did not survive correction for multiple comparisons (Bonferroni-corrected *p* = 0.18 for Cognitive Control scale and Bonferroni-corrected *p* = 0.06 for External Eating score) (see Supplementary Figure 3). Other questionnaire scores did not have a significant relation to the proportion of tastier choices (see Supplementary Table 4). According to the models described in Eqs 5 and 7, the frequency of choosing the tastier option was significantly different across experiments, probably due to differences in the instructions.

## Discussion

### Summary and Interpretation

Food choices are among the most frequent decisions that humans make. These decisions have a substantial influence on people’s health and contribute to being overweight and the development of obesity. Given that correlational studies found social factors to be associated with both eating behavior and emotions, the objective of this study was to investigate the causal effect of social context on food choice, and whether this effect is mediated by emotions. Establishing the presence of such a causal link and its possible mediation by emotions would help identify social risk factors and design better intervention and prevention strategies against obesity and related conditions. This is important because social factors that contribute to obesity can be addressed more easily and at a large scale than other contributors like genetic, homeostatic, and biological factors.

Our results indicate that while lab-induced social contexts induced different emotions, they did not influence food choice. Crucially, there was a significantly positive relation (Bonferroni-corrected) between healthy food choices and dispositional self-control as measured via the BSCS. Apart from this, cognitive restraint of eating as measured via the Cognitive Control subscale of the TFEQ and external eating as measured via the External Eating subscale of the DEBQ correlated with healthy food choices in our experiment, but significantly so only without Bonferroni correction. These findings are in line with previous studies that have associated healthy eating with higher dispositional self-control (Hare et al., 2009; Will Crescioni et al., 2011; Keller and Hartmann, 2016), higher cognitive control of eating behavior, and lower external eating (Elfhag et al., 2007; Keller and Siegrist, 2015). On the basis of these findings, we believe that the food choice task employed in our study captures relevant aspects of participants’ food choices outside the lab.

Importantly, not only external but also internal validity of the food choice task seems to be satisfied: Across all three experiments, in the congruent food choice trials, participants chose the tastier and healthier option significantly more often than the less tasty, less healthy option, indicating that participants made deliberate choices. Further evidence comes from the fact that in the incongruent trials of all three experiments, food choice was predicted by both taste and health attributes (see Figure 3). While the effect of taste was significant in all three data sets, the effect of healthiness was significant in the DG and CBG experiments. All these findings suggest that the food choice task worked and that both taste and health are integrated in the choice, in line with previous findings (Enax et al., 2016).

Our results indicate that the lab-induced negative social contexts did not influence food choice. This is in contrast to previous research which found that the mere perception of a lower socioeconomic status (Cheon and Hong, 2017; Sim et al., 2018) and social exclusion affect food *intake* (Baumeister et al., 2005; Salvy et al., 2011; Senese et al., 2020). This apparent incompatibility of our results with the previous findings may be due to several factors.

First, while our results on the effect of social exclusion on food choice are to some degree comparable to previous research, our results on the effect of unfairness and inferiority are less so due to methodological differences. Previous research on the effects of experiencing unfairness and inferiority has used different methods to induce social disadvantages. For example, Sim et al. (2018) induced the experience of (hypothetical) unfairness through a vignette about being deprived of a deserved outcome: receiving a smaller bonus relative to one’s colleagues. While this represents unfairness, it is different from the unfairness induced by the DG in our study. In the DG, participants are allocated money independent of their past actions, whereas in the study by Sim et al. (2018), the money allocated to the participant according to the vignette is a bonus awarded by the company for which the participant is working. It is conceivable that participants perceive money awarded for some prior performance (even though hypothetical) as more “deserved” than receiving money from an anonymous other participant. Moreover, the management awarding the bonus does not directly gain anything from awarding unequal bonuses. By contrast, in the DG, the dictator’s payoff depends on the amount of money allocated to the recipient; hence, the dictator has an incentive to be selfish. Consequently, it may be easier to regulate the emotional response toward a selfish person than toward an unfair party that does not have a clear benefit from the unfair behavior. Similarly, the explicit framing of the vignette that one’s hypothetical colleagues get more money for the same job may trigger relatively strong social comparison. Regarding lab-induced inferiority, previous studies have relied on asking participants to compare themselves (in writing) to people that they consider better off (Cardel et al., 2016; Cheon and Hong, 2017; Sim et al., 2018). Thus, while the objective is the same (i.e., to induce inferiority through comparison) as in our study, the means through which the comparison was achieved may have triggered different processes, related to more general self-evaluation, than our manipulation. These differences in the triggered processes and the intensity of the emotional responses may account for the different findings.

Second, in these previous studies, food preference was quantified by the amount of food participants ate after social exclusion or after an emotion induction. By contrast, we asked participants to make decisions between food items to be consumed later. Making choices regarding food items to be consumed later (Hare et al., 2009; Maier et al., 2015) results in different neural activity than actually consuming food (de Araujo and Simon, 2009), which suggests that decisions about future consumption and actual consumption of food draw on different processes (Higgs, 2016). While negative social contexts like social exclusion and lab-induced emotions have been found to have an effect on *immediate* food intake, it may be the case that the same do not influence food decisions about *future* food intake.

Future studies should consider directly comparing the effects of negative social contexts and emotions on food decisions about *future* food intake in contrast to *immediate* food intake.

Given that we did not find an effect of the experimental manipulation on observed food choices, it is important to note that in all three experiments, social contexts had a significant effect on the emotion ratings. In the DG experiment, we found that the unfair condition resulted in significantly lower valence ratings in line with the literature (Hewig et al., 2011; Rilling and Sanfey, 2011; Strang et al., 2016) and in higher arousal ratings compared to the neutral and fair conditions. In the CBG experiment, exclusion significantly decreased valence ratings (however, it did not significantly affect arousal ratings). Additionally, analysis of the self-report questions administered after the CBG experiment indicated that, as expected (Williams et al., 2000), participants felt significantly more ignored, less wanted, less invincible, and less powerful after the exclusion condition. Similarly, in the RTR experiment, analysis of the postexperimentally acquired emotion ratings indicated that in line with previous research (Zink et al., 2008), the attained rank (1st, 3rd, 5th) had a significant effect on the emotion ratings, such that being ranked first was associated with higher positive and lower negative emotions, while the reverse was true for when being ranked last. Even so, these induced emotions did not have an effect on food choice.

These findings may, at first glance, differ from the results of previous studies that have shown that both positive and negative lab-induced emotions affect food intake (Baucom and Aiken, 1981; Bongers et al., 2013, 2016; Cardi et al., 2015). More specifically, it has been shown that when under stress and/or in a negative emotional state, individuals prefer energy-dense foods (comfort foods) and often consume more of the same (Leigh Gibson, 2006; Macht, 2008; Bublitz et al., 2010; Konttinen et al., 2010; Cardi et al., 2015). It is worth mentioning, however, that the effects of emotions on food intake are heterogenous, and for specific populations, also *positive* emotions can increase preference for energy-dense foods (Bongers et al., 2016; Ashurst et al., 2018; Evers et al., 2018). Our null finding may thus reflect the inconclusiveness of the previous findings.

More importantly, however, to our knowledge no study has investigated the effects of emotions on food *choice* by using a task similar to ours. The closest to our study is recent work by Privitera et al. (2019) which showed that lab-induced negative emotions increased the number of choices of high-caloric food items “in a buffet-style setting.” Our null finding regarding a potential relationship between emotions and food choice may stem from the fact that in our study, intake of the chosen items was less immediate than in the study by Privitera et al. (2019): Our choice environment more resembled choice in a supermarket than a “buffet-style” choice. That is, while in their study, participants consumed the chosen items almost immediately, while still in the lab, our participants consumed the chosen items only later; and while their participants picked up the items physically from the buffet, our participants merely saw the items displayed on a computer screen.

Another characteristic that makes a direct comparison of our results with those of previous studies difficult is the emotion induction procedure. In these studies, the focus was on emotional eating, such that the emotion induction procedures were more traditional ones, including means such as movies (Bongers et al., 2013; van Strien et al., 2013), vodcasts, perceptual tasks (Kenardy et al., 2003; Cardi et al., 2015), or vignettes (Privitera et al., 2019). By contrast, in our study, the focus is on the effects of negative social contexts on food choice, with emotions as mediators of this possible relation. Even though our methods are comparable to the methods used in other studies in terms of emotion induction strength, assessed via effect sizes (Bongers et al., 2013, 2016; Evers et al., 2013; Cardi et al., 2015), this does not exclude the possibility that different methods induced different kinds of emotions. In line with this, while studies on emotional eating often are based on the induction of emotions such as sadness, happiness, joy, and satisfaction (van Strien et al., 2013; Cardi et al., 2015), the social contexts used in our study have previously been found to evoke feelings of being ignored, feeling powerless, less wanted (Williams et al., 2000; Williams, 2007), feeling of being treated unfairly (Xiao and Houser, 2005). It might be the case that these different emotions evoked by commonly experienced social contexts have no effect or a weaker effect on food choice.

### Limitations and Suggestions for Future Research

Since our objective was to investigate the effect of social context in food choice, and its possible mediation by emotions, in the DG and the CBG we included emotion ratings between each induction and food-choice task. These emotion rating stages may have led participants to be aware of their emotional states, regulate them, and thereby reduce the effect of the negative context. It is important to note that, however, even in the RTR experiments, in which we did not acquire emotion ratings after each trial, we did not find a significant change in participants’ behavior in response to the experimental manipulation. One alternative to address this and assess the emotional state on a trial-by-trial basis for future studies, would consist in collecting emotion-related biomarkers such as measuring skin conductance. Such markers avoid that participants verbalize their state, thereby making it conscious.

The sample size considered in each individual experiment is relatively small. We would like to point out, however, that in all three experiments we employed a within-subject design, which avoids confusing the treatment effect with between-subject variability and is, hence, comparatively powerful. Moreover, sample size was sufficiently large to clearly establish effects of the manipulation on emotions (*p* < 0.001 for all three experiments). We reasoned that if this change in emotions translated to a change in behavior in a similar way in all participants, then the sample size would be sufficient. Furthermore, to increase power, we combined the data from the three experiments and analyzed them jointly. The results of this combined analysis confirmed the results from the separate analyses and suggest that if an effect is present at all, it is relatively small.

For all three studies, we invited healthy participants who occasionally consume snacks. Crucially, across all experiments, the instructions included the statement that participants should choose what they would like to eat in the immediate future, because one of their choices would be implemented at the end of the experiment. However, the degree to which participants were prompted to consider healthiness during their choices differed across studies: In both the DG and the CBG experiment, participants were prompted to consider healthiness while making their choices, whereas this cue was absent in the RTR experiment. While we do observe that the different instructions influenced the *level* of participants’ inclination to make healthy choices, there is no indication of an *interaction* of the instructions with the social context. Crucially, the lack of this interaction is not due to ceiling or floor effects, because there is sufficient room in both directions for the conditions to have an effect (see Figure 4). This is why it is possible to analyze the three experiments jointly. The different instructions even add information and corroborate our null finding: The fact that the different strengths of the health cues influenced participants’ inclination to make healthy choices demonstrates that their decision making was indeed malleable—but the lab-induced social contexts nevertheless failed to have an effect.

Our sample consisted by design of non-dieting, healthy individuals. On this background, a possible explanation of our null result is that food decisions and food-related goals in healthy participants may not be as easily influenced by negative social contexts and emotions as they are in individuals with obesity, binge and restrained eaters (Ganley, 1989; Kenardy et al., 2003; Cardi et al., 2015; Privitera et al., 2019). Furthermore, it is important to mention that our sample consisted mostly of university students, who are not representative of the general population so that also their food-related decisions may diverge from the population’s average. It is possible that subgroups of participants of different socio-demographic background, and of different health status, may be more sensitive to negative social contexts and may be more susceptible to the manipulation of their emotional state than the average subject in our study. Future studies should consider comparing the effects of social context on food choices in different populations. Our study is nevertheless informative by showing that food-related decisions of healthy participants do not seem to be particularly susceptible to negative emotions that result from (acute, non-chronic) disadvantageous social contexts.

Our null finding raises the question whether other types of emotions or more potent negative social contexts might be able to influence food choice. Unfortunately, this points to a fundamental limitation of this line of research: One cannot induce arbitrarily strong, and lasting, negative emotions in an ethically acceptable way. Consequently, there are limits to using negative social contexts—say, sustained, severe exclusion over several weeks—as a tool in research. This, of course, limits our ability to establish a causal effect of social contexts on food choice.

## Conclusion

In this study, we found that experimentally induced social context did not significantly influence food choices of healthy participants. Our data reveal, however, that, in contrast to the emotion-inducing social contexts, dispositional self-control, a more stable characteristic, was significantly related to food choice. More precisely, weaker self-control was associated with a higher number of tastier choices (and, thus, a lower number of healthy choices).

Our work contributes to the literature in several ways. First, we investigated the effects of commonly experienced social contexts on food choice. This is an approach that has not been used in this line of research before, even though social contexts and emotions resulting from social interactions are probably highly relevant for health-related behavior and the disorders associated with it. Second, our approach raises new research questions regarding the nature of emotions that do or do not influence food choices, and whether these influences differ across populations. Third, this study contributes to the literature on the effect of negative social contexts and emotions on food choice and raises the question whether food *intake* and *choice* are influenced to a different degree by social contexts and emotions. Directly comparing the effects of social contexts on food choice and food intake could provide a better understanding of how and when social-context–dependent influences on eating behavior arise. Last but not least, the results of our equivalence test indicate that the effect of different social contexts on food choice is equivalent to 0, and that considering our design, effect sizes of Cohen’s *d* ≥ 0.14 can be excluded. This comes with the caveat, of course, that conducting an equivalence test relies on choosing suitable “equivalence bounds.” The equivalence bounds are supposed to be based on a “smallest effect size of interest” (SESOI). When objective justifications of a SESOI are impossible, a suggestion (Lakens et al., 2018) for picking a SESOI is to derive it from earlier, related studies. This, however, is impossible for a lab experiment with a novel design. We therefore simply report which effect sizes we can rule out based on our data (i.e., Cohen’s *d* ≥ 0.14), and we would like to leave it to our readers to judge whether the minimum effect size is “of interest.”

Overall, this study offers a first attempt to better understand the effects of negative social contexts on food choice in healthy individuals. Knowledge about the presence of an effect—or its *absence*, as in our study—in the healthy population may contribute to a better understanding of the causes and consequences of pathological behavior. We believe that our research will inform the experimental investigation of the link between social disadvantage and food-related decision making. Understanding how social disadvantage does or does not contribute to unhealthy food decisions will help in designing and implementing policies against obesity and eating-related disorders.

## Data Availability Statement

All datasets generated for this study are available online as Supplementary Material.

## Ethics Statement

The studies involving human participants were reviewed and approved by the Ethics committee of the University of Bonn, Germany. The participants provided their written informed consent to participate in this study.

## Author Contributions

QR*:* conceptualization, methodology, software, investigation, formal analysis, visualization, writing—original draft, writing—review and editing. HG*:* methodology, software, investigation, formal analysis, writing—review and editing. XG*:* conceptualization, methodology, software, investigation, formal analysis, writing—review and editing. WZ*:* conceptualization, methodology, investigation. JS: writing—review and editing, supervision. BW: conceptualization, writing—review and editing, supervision. All authors contributed to the article and approved the submitted version.

## Conflict of Interest

The authors declare that the research was conducted in the absence of any commercial or financial relationships that could be construed as a potential conflict of interest.

## Footnotes

1 One should keep in mind that the relation between socioeconomic status and obesity prevalence within a society depends on the country’s economic development (for a review see Malik et al., 2013): In contrast to the developed countries, in developing and underdeveloped countries, obesity rates are likely to be higher among the higher socioeconomic groups. However, recent work by Templin et al. (2019) supports that these countries are quickly catching up, with obesity rates drastically increasing among the poor but remaining unchanged in the wealthy.

2 Laran and Salerno (2013) also report the findings from two experiments (“Study 2” and “Study 3”) that investigate the influence of priming on food *choice*. The food choice in these experiments was purely hypothetical, however, which may be more easily influenced by relatively subtle experimental manipulations than food choice that is incentivized.

3 In Experiment 2, two additional participants (one female, one male) were recruited and showed up, but could not be included in the study. One participant had a higher BMI than the specified criteria allowed, and the other one started but did not complete the experiment.

4 In addition to the previously mentioned questionnaires, in the DG experiment, participants were asked to fill in Beck’s Depression Inventory (BDI-II) (Beck et al., 1996; Hautzinger et al., 2006), the Toronto Alexithymia Scale-20 (TAS-20) (Bagby et al., 1994; Popp et al., 2008), the Neuroticism, Extraversion, and Openness to Experience Five Factor Inventory (NEO-FFI) (Costa and McCrae, 1992; Borkenau and Ostendorf, 1993), a measure of Social Value Orientation (SVO) (Murphy et al., 2011), Behavioral Activation System and Behavioral Inhibition System (BIS/BAS) (Carver and White, 1994; Strobel et al., 2001), the Positive and Negative Affect Schedule (PANAS; *today* version) (Watson et al., 1988; Krohne et al., 1996), the Barratt Impulsiveness Scale Version 11 (BIS-11) (Patton et al., 1995; Hartmann et al., 2011), and a questionnaire assessing the attitudes toward healthy eating (German: Einstellungen zu gesunder Ernährung—EGE) (Diehl, 2006). In the CBG experiment, participants had to fill in only the BDI-II, TAS-20, and PANAS (*today* version). Lastly, in the RTR experiment participants had to fill in only the PANAS (*last-two-weeks* version).
