# Supplementary material for: Do Disadvantageous Social Contexts Influence Food Choice? Evidence From Three Laboratory Experiments
Source: Front Psychol. 2020 Nov 6;11:575170. doi: 10.3389/fpsyg.2020.575170 (PMC7677191; doi:10.3389/fpsyg.2020.575170)
Supplement: Supplementary file 1 [file Data_Sheet_1.pdf]

Supplementary Material

SUPPLEMENTARY TABLE 1 | Descriptive statistics of the administered questionnaires across the three experiments. *M*: mean; *SD*: standard deviation.

Questionnaire	Study	Min.	Max.	Median	*M*	*SD*
**BSCS**						
	Dictator game	2.08	4.54	3.31	3.31	0.57
	Cyberball game	2.08	4.62	3.00	3.12	0.65
	Reaction time ranking task	1.31	4.46	3.08	3.05	0.62
**TFEQ**						
Cognitive control	Dictator game	0	19	7	6.95	4.31
	Cyberball game	0	17	7	7.23	4.86
	Reaction time ranking task	0	15	5	5.74	4.16
Disruption	Dictator game	1	12	6	5.78	2.78
	Cyberball game	1	11	6	5.60	2.90
	Reaction time ranking task	0	15	7	6.80	3.39
Hunger	Dictator game	0	12	8	6.92	3.15
	Cyberball game	0	12	5	5.49	2.76
	Reaction time ranking task	0	12	8	6.94	3.18
**DEBQ**						
Restrained eating	Dictator game	1.1	4.10	2.40	2.42	0.76
	Cyberball game	1.1	4.30	2.40	2.50	0.91
	Reaction time ranking task	1.0	4.30	2.30	2.26	0.79
External eating	Dictator game	1.7	4.70	3.30	3.25	0.58
	Cyberball game	1.7	4.70	3.20	3.13	0.66
	Reaction time ranking task	2.3	4.50	3.40	3.38	0.53
Emotional eating	Dictator game	1.0	3.83	2.29	2.21	0.79
	Cyberball game	1.0	4.25	2.00	2.05	0.80
	Reaction time ranking task	1.0	4.83	2.17	2.29	0.82
**Baseline status**						
Hunger	Dictator game	1	10	7	6.23	2.07
	Cyberball game	3	9	7	6.57	1.46
	Reaction time ranking task	1	9	6	5.77	1.88
Arousal/Excitement	Dictator game	1	6	3	2.98	1.56
	Cyberball game	1	8	3	3.20	1.86
	Reaction time ranking task	1	8	3	3.23	1.89
Valence/Satisfaction	Dictator game	4	9	7	6.88	1.24
	Cyberball game	4	9	7	6.66	1.16
	Reaction time ranking task	3	9	6	5.91	1.51
Hours of Sleep	Dictator game	6	8.0	7	7.15	0.71
	Cyberball game	5	11.0	7	7.10	1.16
	Reaction time ranking task	4	10.0	8	7.67	1.25
**Snack consumption**						
Unhealthy snacks	Dictator game	0.4	3.8	1.4	1.53	0.84
	Cyberball game	0	7.0	1.8	1.92	1.27
	Reaction time ranking task	0.2	6.6	1.8	1.95	1.18
Healthy snacks	Dictator game	1.5	8	5.0	5.14	1.96
	Cyberball game	1.0	8	4.5	4.73	2.16
	Reaction time ranking task	0	20	5.0	6.01	3.98

Notes: n = 40 for the DG, n = 35 for the CBG, and n = 81 for the RTR. *M*: mean, *SD*: standard deviation.

SUPPLEMENTARY TABLE 2 | Mean (M), standard deviation (SD), and mixed-effects linear regression results for the emotion ratings acquired postexperimentally in the Dictator Game experiment.

	Emotion rating	Condition	*M*	*SD*	Statistical comparison (Mixed-effects linear regression results)	Marginal R^2 / Conditional R^2
**DG** (*n* = 40)	Valence	Unfair	3.98	1.87	Neutral – Unfair: β = 1.85, *SE* = 0.30, *z* = 6.19, *p* < 0.001, 95% CI [1.15, 2.55]	0.501 / 0.610
		Neutral	5.83	1.38	Fair – Unfair: β = 3.7, *SE* = 0.30, *z* = 12.37, *p* < 0.001, 95% CI [3.00, 4.40]	
		Fair	7.67	1.21	Fair – Neutral: β =1.85, *SE* = 0.30, *z* = 6.19, *p* < 0.001, 95% CI [1.15, 2.55]	

Notes: SE: Standard error of the estimate, CI: Confidence interval.

SUPPLEMENTARY TABLE 3 | Mediation analyses results. While condition had a significant effect on the emotion ratings (DG and CBG: valence ratings; RTR: positive and negative emotion ratings) on all three experiments, it did not have an effect on the probability of making a tastier food choice. Direct, mediation, and total effects are not significant in any of the experiments.

DG				
**Regressions**				
	**Valence**		**Arousal**	
	**Estimate *(SE)***	**95% CI**	**Estimate *(SE)***	**95% CI**
**DV: Proportion of tastier choices**				
Unfair *vs.* Neutral (c)	0.002 *(0.02)*	[–0.03, 0.03]	0.002 *(0.02)*	[–0.03, 0.03]
Unfair *vs.* Fair (c)	0.002 *(0.02)*	[–0.03, 0.03]	0.002 *(0.02)*	[–0.03, 0.03]
**DV: Emotion ratings**				
Unfair *vs*. Neutral (a)	1.11 *(0.07)* ***	[0.97, 1.26]	–0.64 *(0.07)****	[–0.78, –0.50]
Unfair *vs.* Fair (a)	2.23 *(0.07)* ***	[2.09, 2.38]	–0.56 *(0.07)****	[–0.69, –0.42]
**DV: Proportion of tastier choices**				
Rating (b)	–0.02 *(0.01)*	[–0.04, 0.00]	0.001 *(0.01)*	[–0.02. 0.02]
Unfair *vs.* Neutral (c')	0.01 *(0.02)*	[–0.02, 0.05]	0.003 *(0.02)*	[–0.03, 0.04]
Unfair *vs.* Fair (c')	0.02 *(0.02)*	[–0.02, 0.06]	0.002 *(0.02)*	[–0.03, 0.03]
**Mediation effects**				
**Unfair *vs.* Neutral**				
Average causal mediation	0.02	[–0.005, 0.04]	0.001	[–0.01, 0.01]
Average direct effect	–0.01	[–0.05, 0.02]	–0.002	[–0.04, 0.03]
Total effect	0.01	[–0.03, 0.04]	–0.001	[–0.04, 0.03]
Proportion explained by mediator	0.75	[–14.09, 14.84]	0.06	[–5.59, 3.96]
**Unfair *vs.* Fair**				
Average causal mediation	0.04	[–0.01, 0.08]	0.001	[–0.01, 0.01]
Average direct effect	–0.02	[–0.06, 0.02]	–0.002	[–0.04, 0.03]
Total effect	0.01	[–0.02, 0.05]	–0.002	[–0.03, 0.03]
Proportion explained by mediator	1.49	[–18.76, 14.68]	0.02	[–4.95, 3.93]

**CBG**				
**Regressions**				
	**Valence**		**Arousal**	
	**Estimate *(SE)***	**95% CI**	**Estimate *(SE)***	**95% CI**
**DV: Proportion of tastier choices**				
Exclusion *vs.* Inclusion (c)	–0.01 *(0.02)*	[–0.04, 0.02]	–0.01 *(0.02)*	[–0.04, 0.02]
**DV: Emotion ratings**				
Exclusion *vs.* Inclusion (a)	0.33 *(0.08)******	[0.17, 0.49]	–0.09 *(0.08)*	[–0.24, 0.06]
**DV: Proportion of tastier choices**				
Rating (b)	–0.005 *(0.01)*	[–0.03, 0.02]	0.001 *(0.01)*	[–0.02, 0.03]
Exclusion *vs.* Inclusion (c')	–0.01 *(0.02)*	[–0.04, 0.02]	–0.01 *(0.02)*	[–0.04, 0.02]
**Mediation effects**				
**Exclusion *vs.* Inclusion**				
Average causal mediation	–0.001	[–0.01, 0.01]	–0.0001	[–0.004, 0.00]
Average direct effect	–0.01	[–0.04, 0.02]	–0.01	[–0.04, 0.02]
Total effect	–0.01	[–0.04, 0.02]	–0.01	[–0.04, 0.02]
Proportion explained by mediator	0.07	[–2.47, 2.82]	0.001	[–0.71, 0.73]
**RTR**				
**Regressions**				
	**Positive emotions**		**Negative emotions**	
	**Estimate *(SE)***	**95% CI**	**Estimate *(SE)***	**95% CI**
**DV: Proportion of tastier choices**				
5th *vs.* 3rd (c)	–0.01 *(0.02)*	[–0.04, 0.03]	–0.01 *(0.02)*	[–0.04, 0.03]
5th *vs.* 1st (c)	–0.001 *(0.02)*	[–0.04, 0.03]	–0.001 *(0.02)*	[–0.04, 0.03]
**DV: Emotion ratings**				
5th *vs.* 3rd (a)	1.76 *(0.21)******	[1.35, 2.17]	–1.19 *(0.23)******	[–1.65, –0.73]
5th *vs.* 1st (a)	5.00 *(0.21)******	[4.59, 5.42]	–3.33 *(0.24)******	[–3.79, –2.87]
**DV: Proportion of tastier choices**				
Rating (b)	0.02 *(0.01)*	[–0.01, 0.06]	–0.01 *(0.01)*	[–0.03, 0.02]
5th *vs.* 3rd (c')	–0.02 *(0.02)*	[–0.07, 0.02]	–0.01 *(0.02)*	[–0.05, 0.03]
5th *vs.* 1st (c')	–0.05 *(0.04)*	[–0.12, 0.02]	–0.01 *(0.03)*	[–0.06,0.04]
**Mediation effects**				
**5th *vs.* 3rd**				
Average causal mediation	–0.04	[–0.10, 0.02]	–0.01	[–0.04, 0.02]
Average direct effect	0.02	[–0.02, 0.06]	0.01	[–0.03, 0.05]
Total effect	–0.02	[–0.07, 0.03]	0.005	[–0.04, 0.04]
Proportion explained by mediator	1.52	[–9.28, 13.53]	0.23	[–7.64, 8.19]
**5th *vs.* 1st**				
Average causal mediation	–0.12	[–0.28, 0.04]	–0.02	[–0.10, 0.06]
Average direct effect	0.05	[–0.03, 0.12]	0.01	[–0.04, 0.06]
Total effect	–0.07	[–0.17, 0.03]	–0.01	[–0.07, 0.05]
Proportion explained by mediator	1.61	[–1.82, 5.96]	1.23	[–7.43, 14.69]

Notes: n = 40 for the DG, n = 35 for the CBG, and n = 81 for the RTR. *SE*: Standard error of the estimate, CI: Confidence interval; *** $p < 0.001$.

SUPPLEMENTARY TABLE 4 | Mixed-effects logistic regression results with proportion of tasty choices as dependent variable. Condition had no significant effect on the proportion of tasty choices when controlling for the different questionnaire scores and type of manipulation. BSCS score, TFEQ Cognitive control score, and DEBQ External score were significantly related to the proportion of tasty choices. All questionnaire scores were *z*-scored.

	Explanatory variable	Mixed-effects regression results	Marginal R^2 / Conditional R^2
**BSCS**			
	Intercept	β = 0.62, *SE* = 0.03, $t_{(157.3)}$ = 19.63, *p* < 0.001, 95% CI [0.56, 0.68]	0.421 / 0.917
	BSCS score	β = –0.06, *SE* = 0.02, $t_{(174.6)}$ = –4.11, *p* < 0.001, 95% CI [–0.09, –0.03]	
	Condition	β = –0.0004, *SE* = 0.01, $t_{(154)}$ = –0.05, *p* = 0.96, 95% CI [–0.02, 0.02]	
	CBG *vs.* DG	β = –0.21, *SE* = 0.04, $t_{(151.99)}$ = –4.79, *p* < 0.001, 95% CI [–0.29, –0.12]	
	CBG *vs.* RTR	β = 0.14, *SE* = 0.04, $t_{(151.99)}$ = 3.70, *p* = 0.0004, 95% CI [0.06, 0.21]	
	BSCS score × Condition	β = 0.01, *SE* = 0.01, $t_{(154)}$ = 1.59, *p* = 0.11, 95% CI [–0.0, 0.03]	
**TFEQ**			
Cognitive Control	Intercept	β = 0.63, *SE* = 0.03, $t_{(157)}$ = 19.33, *p* < 0.001, 95% CI [0.56, 0.69]	0.392 / 0.916
	Cognitive Control score	β = –0.04, *SE* = 0.02, $t_{(174.03)}$ = –2.37, *p* = 0.02, 95% CI [–0.07, –0.01]	
	Condition	β = –0.0004, *SE* = 0.01, $t_{(154)}$ = –0.05, *p* = 0.96, 95% CI [–0.02, 0.02]	
	CBG *vs.* DG	β = –0.23, *SE* = 0.04, $t_{(151.99)}$ = –5.15, *p* < 0.001, 95% CI [–0.31, –0.14]	
	CBG *vs.* RTR	β = 0.13, *SE* = 0.04, $t_{(151.99)}$ = 3.4, *p* = 0.001, 95% CI [0.06, 0.21]	
	Cognitive Control score × Condition	β = –0.002, *SE* = 0.01, $t_{(154)}$ = –0.25, *p* = 0.80, 95% CI [–0.02, 0.01]	
Disruption	Intercept	β = 0.62, *SE* = 0.03, $t_{(156.8)}$ = 18.66, *p* < 0.001, 95% CI [0.55, 0.68]	0.373 / 0.916
	Disruption score	β = –0.01, *SE* = 0.02, $t_{(172.96)}$ = –0.64, *p* = 0.52, 95% CI [–0.04, 0.02]	
	Condition	β = –0.0004, *SE* = 0.01, $t_{(154)}$ = –0.05, *p* = 0.96, 95% CI [–0.02, 0.02]	
	CBG *vs.* DG	β = –0.22, *SE* = 0.04, $t_{(151.99)}$ = –4.99, *p* < 0.001, 95% CI [–0.31, –0.14]	
	CBG *vs.* RTR	β = 0.15, *SE* = 0.04, $t_{(151.99)}$ = 3.81, *p* = 0.0002, 95% CI [0.07, 0.23]	
	Disruption score × Condition	β = –0.009, *SE* = 0.01, $t_{(154)}$ = –1.12, *p* = 0.26, 95% CI [–0.03, 0.01]	

Hunger	Intercept	β = 0.63, *SE* = 0.03, $t_{(156.80)}$ = 18.90, p < 0.001, 95% CI [0.56, 0.69]	0.377 / 0.916
	Hunger score	β = 0.02, *SE* = 0.02, $t_{(173.1)}$ = 1.36, p = 0.18, 95% CI [−0.01, 0.05]	
	Condition	β = −0.0004, *SE* = 0.01, $t_{(154)}$ = −0.05, p = 0.96, 95% CI [−0.02, 0.02]	
	CBG *vs.* DG	β = −0.23, *SE* = 0.04, $t_{(151.99)}$ = −5.20, p < 0.001, 95% CI [−0.32, −0.15]	
	CBG *vs.* RTR	β = 0.13, *SE* = 0.04, $t_{(151.99)}$ = 3.39, p = 0.001, 95% CI [0.06, 0.21]	
	Hunger score × Condition	β = 0.002, *SE* = 0.01, $t_{(154)}$ = 0.24, p = 0.81, 95% CI [−0.01, 0.02]	
**DEBQ**			
External Eating	Intercept	β = 0.63, *SE* = 0.03, $t_{(156.92)}$ = 19.32, p < 0.001, 95% CI [0.57, 0.69]	0.392 / 0.917
	External Eating score	β = 0.04, *SE* = 0.02, $t_{(173.5)}$ = 2.75, p = 0.007, 95% CI [0.01, 0.08]	
	Condition	β = −0.0004, *SE* = 0.01, $t_{(154)}$ = −0.05, p = 0.96, 95% CI [−0.02, 0.02]	
	CBG *vs.* DG	β = −0.23, *SE* = 0.04, $t_{(151.99)}$ = −5.26, p < 0.001, 95% CI [−0.32, −0.14]	
	CBG *vs.* RTR	β = 0.13, *SE* = 0.04, $t_{(151.99)}$ = 3.29, p = 0.001, 95% CI [0.05, 0.20]	
	External Eating score × Condition	β = −0.01, *SE* = 0.01, $t_{(154)}$ = −1.49, p = 0.14, 95% CI [−0.03, 0.00]	
Restrained Eating	Intercept	β = 0.62, *SE* = 0.03, $t_{(156.93)}$ = 19.02, p < 0.001, 95% CI [0.56, 0.69]	0.379 / 0.916
	Restrained Eating score	β = −0.02, *SE* = 0.02, $t_{(173.68)}$ = −1.51, p = 0.13, 95% CI [−0.06, 0.01]	
	Condition	β = −0.0004, *SE* = 0.01, $t_{(154)}$ = −0.05, p = 0.96, 95% CI [−0.02, 0.02]	
	CBG *vs.* DG	β = −0.23, *SE* = 0.04, $t_{(152)}$ = −5.10, p < 0.001, 95% CI [−0.31, −0.14]	
	CBG *vs.* RTR	β = 0.14, *SE* = 0.04, $t_{(152)}$ = 3.51, p = 0.0006, 95% CI [0.06, 0.21]	
	Restrained Eating score × Condition	β = −0.001, *SE* = 0.01, $t_{(154)}$ = −0.16, p = 0.87, 95% CI [−0.02, 0.01]	
Emotional Eating	Intercept	β = 0.62, *SE* = 0.03, $t_{(156.85)}$ = 18.82, p < 0.001, 95% CI [0.56, 0.69]	0.371 / 0.916
	Emotional Eating score	β = 0.01, *SE* = 0.02, $t_{(173.45)}$ = 0.77, p = 0.45, 95% CI [−0.02, 0.04]	
	Condition	β = −0.0004, *SE* = 0.01, $t_{(154)}$ = −0.05, p = 0.96, 95% CI [−0.02, 0.02]	
	CBG *vs.* DG	β = −0.23, *SE* = 0.04, $t_{(151.99)}$ = −5.05, p < 0.001, 95% CI [−0.31, −0.14]	
	CBG *vs.* RTR	β = 0.14, *SE* = 0.04, $t_{(151.99)}$ = 3.59, p = 0.0004, 95% CI [0.06, 0.22]	
	Emotional Eating score × Condition	β = −0.001, *SE* = 0.01, $t_{(154)}$ = −0.17, p = 0.86, 95% CI [−0.02, 0.01]	

Notes: n = 40 for the DG, n = 35 for the CBG, and n = 81 for the RTR. 0 = Negative social context condition, 1 = Positive social context condition; *SE*: Standard error of the estimate.

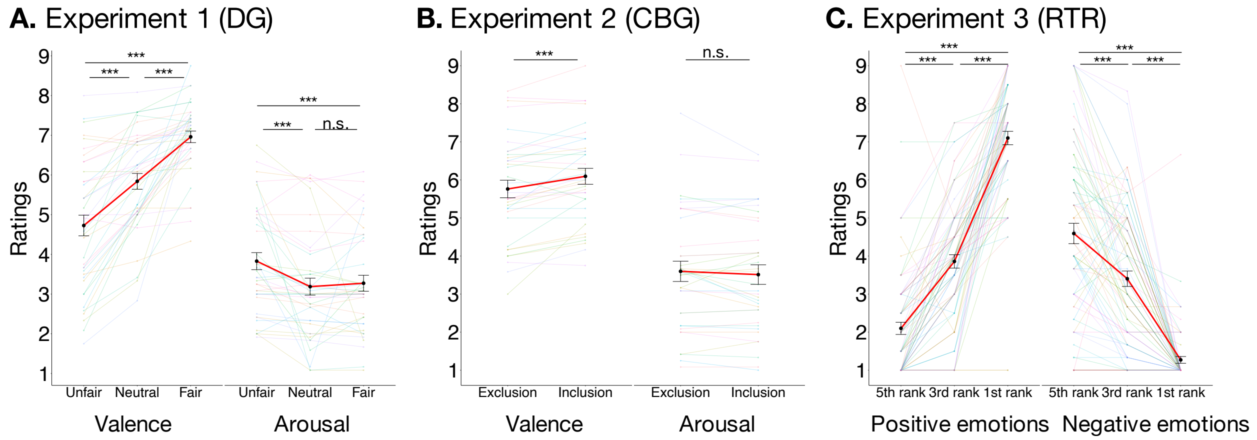

SUPPLEMENTARY FIGURE 1 | Distribution of emotion ratings acquired in the DG **(A)**, the CBG **(B)** and the RTR **(C)** experiments. **(A)** In the DG experiment condition has a significant effect on the valence ($\chi^2_{(2)} = 712.77$, $p < 0.001$, marginal $R^2 = 0.26$) and arousal ratings ($\chi^2_{(2)} = 93.95$, $p < 0.001$, marginal $R^2 = 0.03$). Tukey *post hoc* tests revealed that in terms of valence all pairwise comparisons are significant (Neutral – Unfair: β = 1.11, *SE* = 0.07, 95% CI [0.94, 1.28], *z* = 15.18; Fair – Unfair: β = 2.23, *SE* = 0.07, 95% CI [2.06, 2.41], *z* = 30.46; Fair – Neutral: β = 1.12, *SE* = 0.07, 95% CI [0.95, 1.29], *z* = 15.29), while in terms of arousal only the difference between neutral and fair condition is not significant (Neutral – Unfair: β = –0.64, *SE* = 0.07, 95% CI [–0.81, –0.48], *z* = –9.07; Fair – Unfair: β = –0.56, *SE* = 0.07, 95% CI [–0.72, –0.39], *z* = –7.86; Fair – Neutral: β = 0.09, *SE* = 0.07, 95% CI [–0.08, 0.25], *z* = 1.21). **(B)** In the CBG experiment, valence ratings are significantly higher in the inclusion compared to the exclusion condition ($\chi^2_{(1)} = 16.81$, $p < 0.001$, marginal $R^2 = 0.009$; Inclusion – Exclusion: β = 0.33, *SE* = 0.08, 95% CI [0.17, 0.49], *z* = 4.12), and arousal ratings are not significantly different between the two conditions ($\chi^2_{(1)} = 1.25$, $p = 0.26$, marginal $R^2 = 0.0005$; Inclusion – Exclusion: β = –0.09, *SE* = 0.08, 95% CI [–0.24, 0.06], *z* = –1.12). **(C)** In the RTR experiment, condition has a significant effect on the positive ($\chi^2_{(2)} = 265.61$, $p < 0.001$, marginal $R^2 = 0.65$), and negative emotion ratings ($\chi^2_{(2)} = 133.79$, $p < 0.001$, marginal $R^2 = 0.37$). All Tukey *post hoc* tests are significant (Positive emotions: 3rd – 5th: β = 1.76, *SE* = 0.21, 95% CI [1.27, 2.25], *z* = 8.45; 1st – 5th: β = 5.01, *SE* = 0.21, 95% CI [4.52, 5.50], *z* = 23.90; 1st – 3rd: β = 3.25, *SE* = 0.21, 95% CI [2.76, 3.74], *z* = 15.51; Negative emotions: 3rd – 5th: β = –1.19, *SE* = 0.23, 95% CI [–1.73, –0.64], *z* = –5.12; 1st – 5th: β = –3.32, *SE* = 0.23, 95% CI [–3.87, –2.77], *z* = –14.18; 1st – 3rd: β = –2.13, *SE* = 0.23, 95% CI [–2.68, –1.58], *z* = –9.10). The black dots are mean values across participants. Error bars represent the standard error of the mean. Effects are estimated using linear mixed-effects models. *** $p < 0.001$, ** $p <$ 0.01, “n.s.”: not significant.

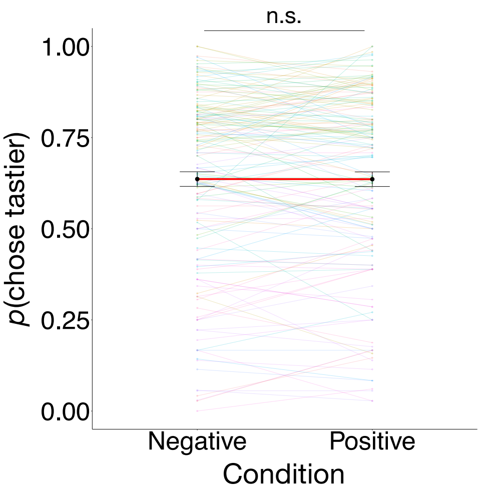

SUPPLEMENTARY FIGURE 2 | Probability of choosing the tastier item in the negative and positive social context conditions. Condition had no significant effect on the probability of choosing the tastier food item. The colored dots are data points from individual participants (n = 156), and the black dots are mean probabilities across participants. For better visualization of the differences between conditions, individual observations are connected with color-coded lines, whereas the mean values are connected with a red line. Error bars indicate the standard error of the mean. “n.s.”: not significant.

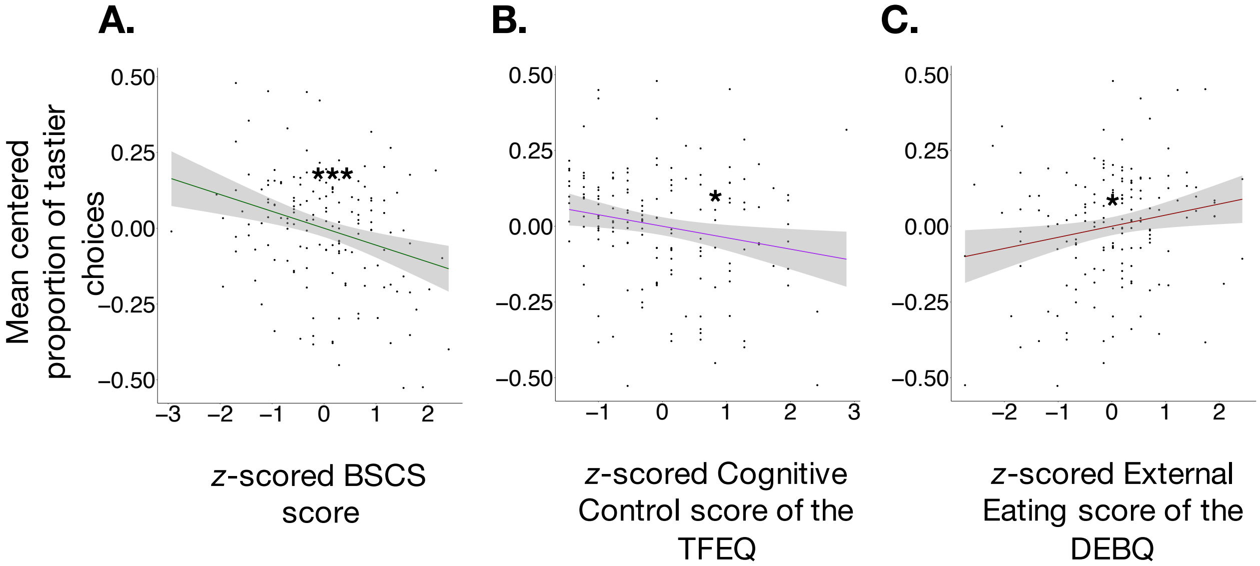

SUPPLEMENTARY FIGURE 3 | Mean-centered proportion of tastier choices as a function of BSCS **(A)**, Cognitive Control **(B)**, and External Eating **(C)**. Dispositional self-control as assessed via the BSCS (r = –0.29, $t_{(154)}$ = –3.78) and Cognitive Control of eating behavior as assessed via the TFEQ (r = –0.20, $t_{(154)}$ = –2.5) are negatively correlated with the proportion of tasty choices. External Eating as assessed via the DEBQ is positively correlated with the proportion of tasty choices (r = 0.19, $t_{(154)}$ = 2.42). * $p < 0.05$; *** $p < 0.001$. The correlation between proportion of tastier choices and Cognitive Control score of the TFEQ **(B)**, and the correlation between the proportion of tastier choices and the External Eating score of the DEBQ **(C)** do not survive Bonferroni correction.
