# Supplementary material for: Do Disadvantageous Social Contexts Influence Food Choice? Evidence From Three Laboratory Experiments
Source: Front Psychol. 2020 Nov 6;11:575170. doi: 10.3389/fpsyg.2020.575170 (PMC7677191; doi:10.3389/fpsyg.2020.575170)
Supplement: Supplementary file 3 [file Data_Sheet_3.pdf]

Information (I.) and instructions (II.) provided to the participants in the Dictator Game experiment

As the original instructions were in German, we provide here an English translation of the same. We show the original figures presented to the participants with English translation of the words appearing in those figures below the figures.

I. Information for participants

Dear participant,
in the following we would like to inform you about the course of the scientific experiment "The investigation of neuronal correlates of food decision making". Please read this information carefully. Please contact us if you have any further questions.

The aim of this study is to investigate the neurological processes underlying decisions between two food items. The experiment consists of different parts. On the day of the study, after discussing the instructions and answering the comprehension questions, you will evaluate different food items on the computer in terms of how tasty and how healthy you think they are. This is followed by an experiment in a functional magnetic resonance imaging (fMRI) scanner. You will then be asked to answer questionnaires, after which you will be reimbursed for your participation.
In the following we would like to inform you about the procedure in more detail:

Instructions and comprehension questions: Please read the following instructions carefully. On the day of the experiment, the investigators will be available to answer any questions you may have. On the day of the experiment, you can expect to receive written comprehension questions regarding the tasks explained in the instructions. This part takes about 10 minutes.

Food rating task: In this part of the experiment, images of individual food items are presented to you on a computer screen. Your task is to evaluate the food items in terms of how tasty and how healthy you think these items are. This part takes about 30 minutes.

fMRI experiment: The fMRI experiment consists of three recurring elements (you will find a detailed description of these elements in the "Instructions for the tasks"):

1. **Money allocations from the "Dictator Game":** This element shows you the amount of money split from a "Dictator Game". In such a "Dictator Game" a "dictator" has the possibility to divide a given amount of money between him/herself and a "recipient". This means that the dictator "dictates" the decision, while the recipient cannot influence this decision. You have the role of the recipient. Dictators have determined the distribution of money at an earlier stage.

Some of the displayed money distributions will, however, not come from a dictator but will be generated by a computer.

2. **Emotion ratings:** An emotional evaluation will take place for the following element. For this purpose, you will be shown two scales in this task, which represent different aspects of emotions: valence and arousal. Since the scales are not displayed for a long time, it is very important that you do not think about it for a long time, but rather that you indicate your emotional state quickly and honestly at the appropriate moment.
3. **Food choices:** In this task you will see two different food items next to each other on the screen. You have to decide which food you would rather eat. Several food choices follow one after the other.

You will find a more detailed information about the tasks you are to perform in the scanner in the "Instructions for the tasks".
The fMRI experiment takes about 40 minutes. Afterwards, a structural image of your brain is taken, which takes about 10 minutes.

Questionnaires: After the fMRI experiment, you will be asked to fill in questionnaires on a computer. This part will take about 20 minutes. These questions are also based on the principle that correct and incorrect answers do not exist and that your honest responses best serve the purpose of the investigation.

Reimbursement: At the end of the whole experiment, which will take up to 2 hours, the payout will take place. You will receive a participation fee of €20. In addition to this fee, you will receive one of the money distributions from the Dictator Game displayed in the fMRI experiment. Only distributions made by dictators are "payoff-relevant" (i.e., one of the distributions made by a dictator is paid out) and the rounds generated by a computer are "not payoff-relevant" (i.e., they are not paid out). The final part of the payoff is the implementation of one of the food decisions you made in the fMRI experiment. This means that you will receive one of the food items you selected and are to consume it on site.

We want you to feel good during and after the experiment. If you feel uncomfortable about anything, if you do not understand something or want to get more precise information on anything, please inform us and ask immediately.

Your participation in the study is voluntary. You can withdraw your consent at any time or stop the experiment at any time without giving any reasons. You will not suffer any disadvantage as a result. The data obtained will be destroyed at your request after completion of the study.

II. Instructions for the tasks

Food rating task

In the food rating task, you will be shown images of food items on a computer screen. You can rate each food item using the mouse and the scale displayed under the food item (see the following figure). You will rate food items in two blocks, once in terms of taste and once in terms of health. Which of the evaluation blocks (taste / health) is queried first is random, but it will be indicated on the screen before the start of the block.

If you do not know the displayed food item, please make an assessment as best as you can. Please look at each food item carefully but make your decisions quickly and do not think too long. This part takes about 30 minutes. Please give your opinion as accurately as possible, because your evaluations determine the further course of the experiment.

Here is an example from each block for clarification:

Health:

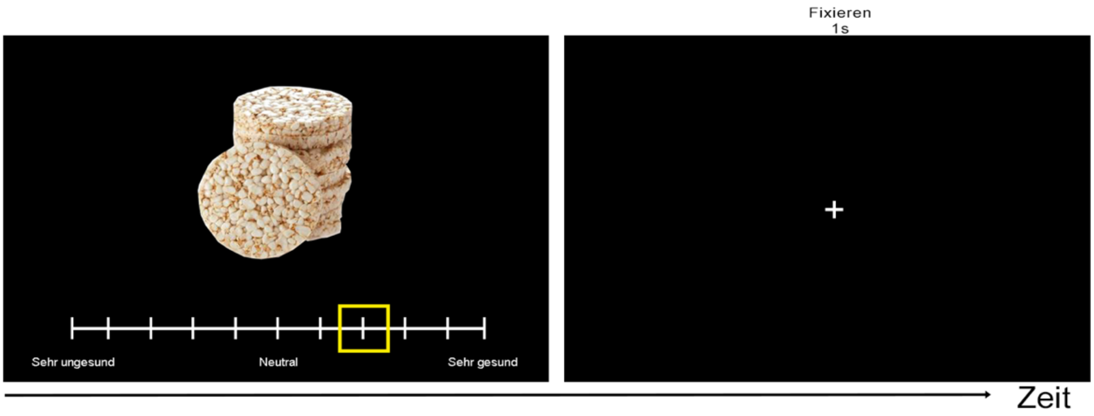

Sehr ungesund = very unhealthy; Sehr gesund = very healthy; Fixieren = fixate; Zeit = time

Taste:

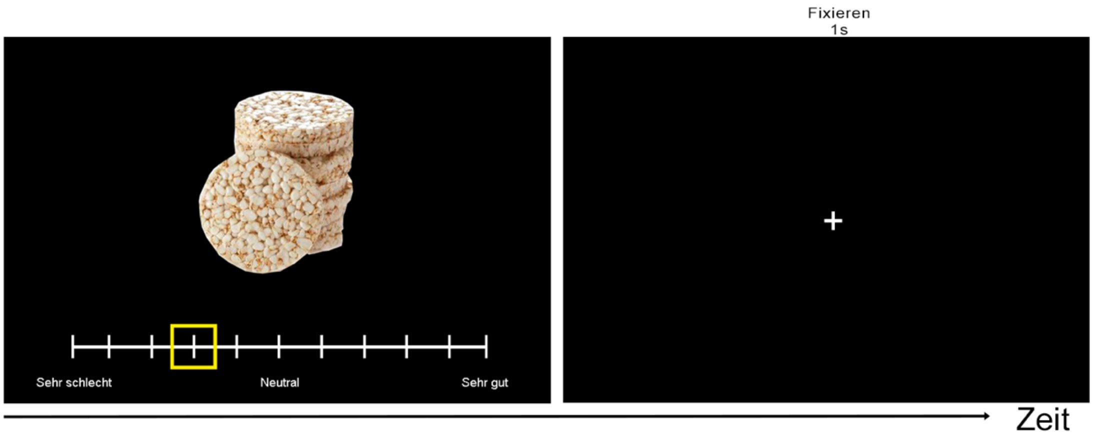

Sehr schlecht = very bad; Sehr gut = very good; Fixieren = fixate; Zeit = time

fMRI experiment

The subsequent fMRI experiment lasts about 40 minutes and is described in more detail on the following pages. As already described, it consists of three recurring elements (see figure below):

1. Money allocation from the Dictator Game

2. Emotion rating

3. Food choices

These two elements have already been mentioned in the "Information for participants" and will now be explained in more detail. The following figure shows the procedure in the scanner. In between the different elements of the experiment you will be shown "fixation crosses" (white cross), please just look at them while waiting.

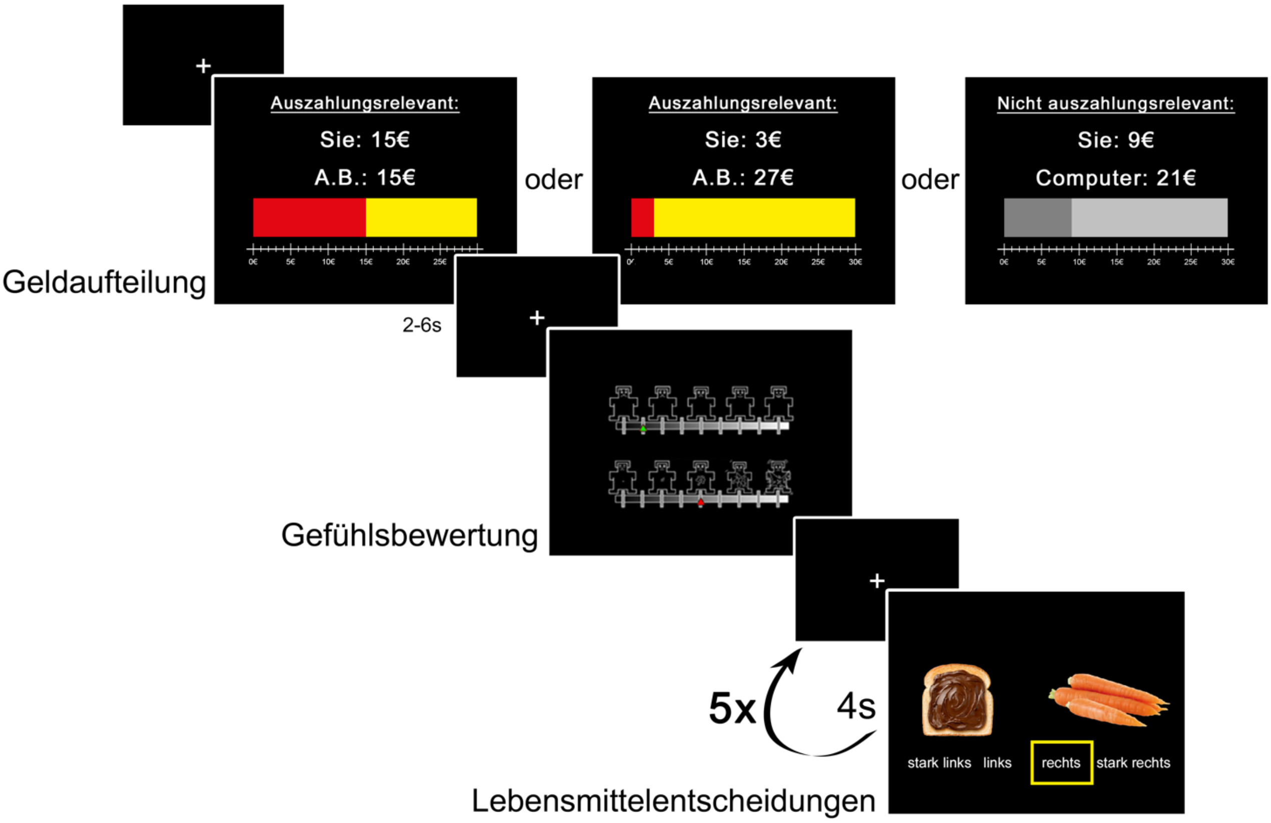

Geldaufteilung = money allocation; oder = or; Auszahlungsrelevant = payoff-relevant; Nicht auszahlungsrelavant = not payoff-relevant; Sie = you; Gefühlsbewertung = Emotion rating; Lebensmittelentscheidungen = food choices; stark links = strong left; links = left; rechts = right; stark rechts = strong right

1. Money allocation from the Dictator Game

Allocation by a dictator

At the beginning of each round you will be shown money splits from a Dictator Game. Another participant, the "dictator", has divided up €30 between him/herself and you. This means that the dictator "dictates" the decision, while the recipient cannot influence this decision. These decisions were made on 25.02.2016 in another laboratory of the University of Bonn, the BonnEconLab. You are the "recipient" of these divisions and have no influence on them.

The following figure shows an example of how the money allocations will be shown to you in this experiment.

Payoff-relevant money allocation by a dictator:
(Figure A)

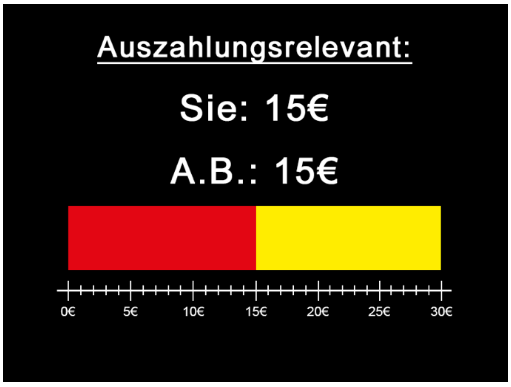

Auszahlungsrelevant = payoff-relevant; Sie = you

The 40 dictators have been informed that you, as the recipient, will also take part in this Dictator Game. The dictators then entered the initials of their first and last names. These initials will be displayed with the corresponding money allocations of the dictator in the experiment (see Fig. A).

There will be several dictators in the experiment, i.e., you can expect similar pictures as in Figure A). All dictators' decisions are potentially relevant for your payoff, therefore "payoff-relevant" (i.e., the money allocations made by a dictator will be paid out) (Figure A). But (!) only <u>one</u> of these allocations will be paid out to you. You will find out which one this will be after the experiment. Therefore, during the experiment, keep in mind that <u>each</u> of the money allocations made by a dictator can potentially be the one that is relevant for your payoff!

Each dictator only split the money once during his/her experiment. The dictators knew, however, that the money allocations that they made would affect a specific recipient (you and your payoff). The dictators were informed that your experiment would take about 2.5 hours, while their experiment only took about 30 minutes.

The dictators 1-40 had the possibility to choose between two alternative divisions of the €30 (alternatives A or B):

Dictators	Alternative A		Alternative B
Dictators 1-10	You €15 / Dictator €15	or	You €6 / Dictator €24
Dictators 11-20	You €14 / Dictator €16	or	You €5 / Dictator €25
Dictators 21-30	You €13 / Dictator €17	or	You €4 / Dictator €26
Dictators 31-40	You €12 / Dictator €18	or	You €3 / Dictator €27

This means that if you see in the experiment that a dictator allocated to you €13 and to him/herself €17, you know that he/she had the choice between this alternative A and alternative B where he/she could have allocated €4 to you and €26 to him/herself.

The experiment conducted on the 25.02.2016 was finished after the dictators made the money allocations.

Allocation by a computer
Some of the displayed money allocations will not come from a dictator. These allocations are generated by a computer and are not payoff-relevant, i.e., "not relevant for payoff" (i.e., will not be paid out) (see Figure B). Under no circumstances will you be paid out the part allocated to you by a computer. During the experiment, if the presented money allocation is generated by a computer, the name "Computer" (Figure B) will appear on the screen and the bar displaying the money allocation will be grey in color. If the presented money allocation comes from a dictator, the initials of the corresponding dictator will be displayed on the screen (Figure A).

Example of a non-payoff-relevant money allocation by a computer:
(Figure B)

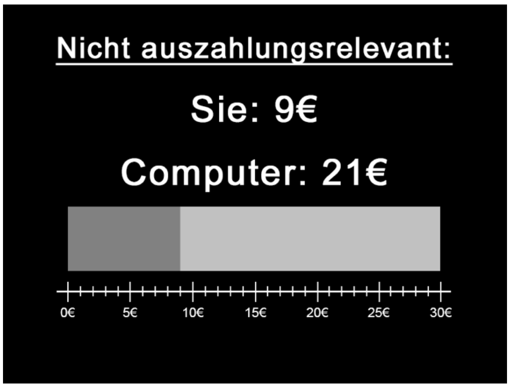

Nicht auszahlungsrelevant = not payoff-relevant; Sie = you

While you are shown the money allocations from the Dictator Game, you do not need to do anything or press anything. Please just look at the decision and pay attention to the amount of the money allocation and whether it was made by a dictator or a computer.

2. Emotion ratings

After the presentation of the money allocations, an emotion rating takes place. In this task we ask you to express your current emotional state by selecting symbols. There are no right or wrong answers. In this task you will be shown two scales representing different aspects of emotions: valence and arousal.

Scale I: Valence

You see here that each figure varies along the scale between happy and sad. At one end you feel completely cheerful, delighted, satisfied, happy, hopeful. At the other end of the scale you are completely sad, annoyed, unsatisfied, melancholic, desperate, bored.

traurig (*sad*)
genervt (*annoyed*)
unzufrieden (*unsatisfied*)
melancholisch (*melancholic*)
verzweifelt (*desperate*)
gelangweilt (*bored*)

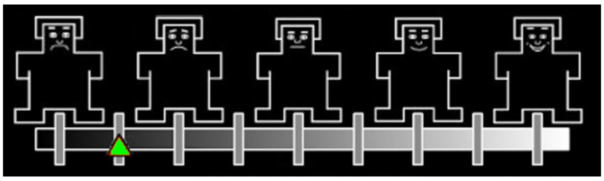

Fröhlich (*cheerful*)
Erfreut (*delighted*)
Zufrieden (*satisfied*)
Glücklich (*happy*)
hoffnungsvoll (*hopeful*)

Scale II: Arousal

You can see here that each figure varies along the scale between arousal and calm. At one end you feel completely stimulated, agitated, hectic, nervous, wide-awake or aroused. At the other end of the scale you are completely relaxed, calm, sluggish, dizzy, tired, unexcited.
You can also choose intermediate levels on the sliding scale.

entspannt (*relaxed*)
ruhig (*calm*)
träge (*sluggish)*
benommen (*dizzy*)
müde (*tired*)
unaufgeregt (*unexcited*)

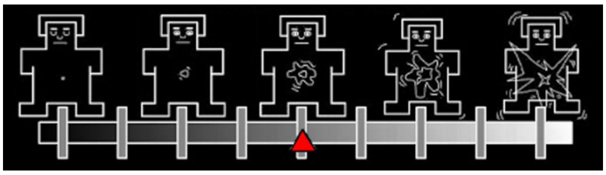

angeregt (*stimulated)*
aufgeregt (*agitated*)
hektisch (*hectic*)
nervös (*nervous)*
hellwach (*wide-awake*)
aufgerüttelt (*aroused*)

In the fMRI experiment, the red triangle (the cursor) will appear randomly at a position on the first scale. You make the selection in the scanner by pressing the buttons on the MRI response grips. The following key assignment applies:

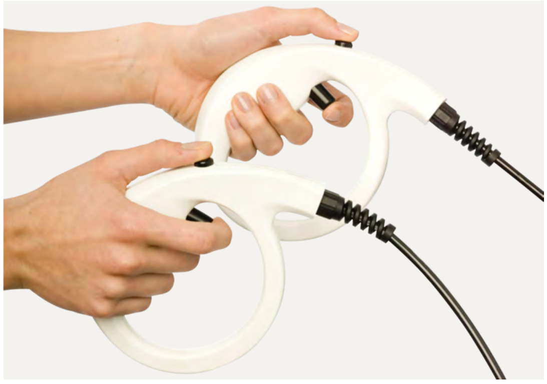

Press the left and right index finger to move the cursor left and right accordingly. Confirm the selection with the button on your right thumb. The cursor will turn green to indicate that your entry has been registered. Repeat the process in the same way for scale II. Since the scales are only displayed for 6 seconds, it is very important that you state your emotional state at the appropriate moment quickly and honestly without thinking about it for too long!

3. Food choices

Following this, you have the choice between two different food items. You have the possibility to indicate whether you prefer the respective food item more strongly or less strongly (strongly left/left/right/strongly right). One of the choices you make will be handed to you at the end of the experiment and should actually be consumed on the spot. Therefore, make sure that in each round you only select products that you really want to eat after the experiment. In the decision tasks you should pursue an overriding goal. The goal in each decision round will be to eat **as healthy as possible**. You will **only have up to 4 seconds** to make your decision. If you need more than 4 seconds, your answer will not be evaluated. After each food choice trial, you will see a fixation cross in the middle of the screen. Please fixate on the cross and lie still until you can make the next choice.

Here is an example of two successive rounds of decisions:

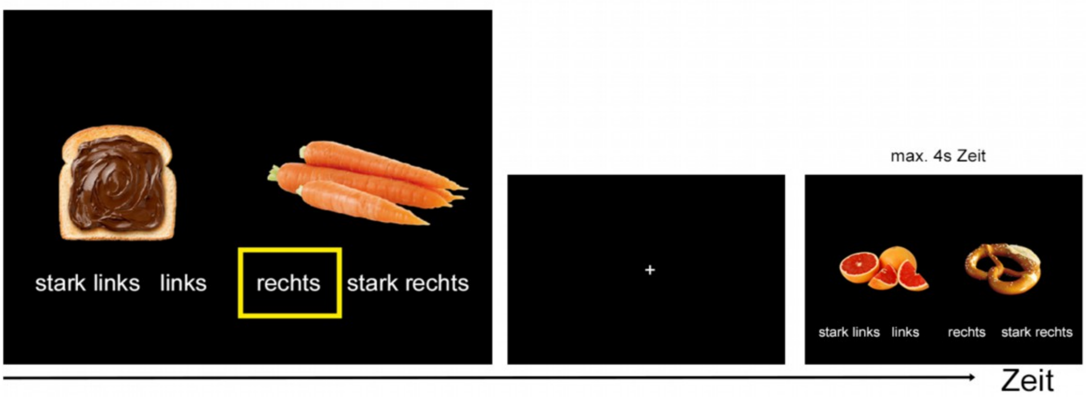

stark links = strong left; links = left; rechts = right; stark rechts = strong right; Zeit = time

In this task, you will indicate your responses by pressing the buttons on the MRI response grips. The following key assignment applies:

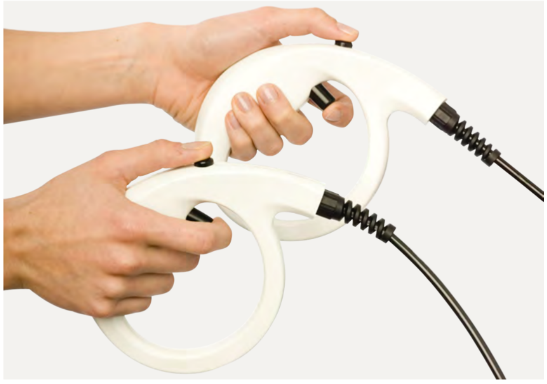

For your decision, please press the left index finger for the left product (left) or the right index finger for the right product (right). If you have a strong preference for the left product, please press the left thumb (strong left) or the right thumb if you have a strong preference for the right product (strong right). As soon as you press a button, the decision is registered, and this is indicated by a yellow frame around the corresponding product (see illustration above). The experiment then continues automatically with the fixation cross.

Several food decisions follow one after the other before the next round with the Dictator Game starts again.

After the 40-minute fMRI experiment, a structural image of your brain will be taken; this will take about 10 minutes. During the structural measurement you will not perform any tasks and you may close your eyes.
Please take care not to move your head during the fMRI experiment and during the acquisition of the structural image to ensure good image quality! This is very important for the further evaluation of the data.

Thank you very much for your participation!
