# Supplementary material for: Do Disadvantageous Social Contexts Influence Food Choice? Evidence From Three Laboratory Experiments
Source: Front Psychol. 2020 Nov 6;11:575170. doi: 10.3389/fpsyg.2020.575170 (PMC7677191; doi:10.3389/fpsyg.2020.575170)
Supplement: Supplementary file 4 [file Data_Sheet_4.pdf]

Information zur Teilnahme an der wissenschaftlichen Untersuchung

„Die Untersuchung neuronaler Korrelate während Lebensmittelentscheidungen“

Verantwortlicher Arzt: Prof. Dr. med. Bernd Weber

Department of NeuroCognition, Life & Brain Center und Klink für Epileptologie, Universitätsklinikum Bonn

Liebe/r an der Studie Interessierte/r,

im Folgenden möchten wir Sie über den Ablauf des wissenschaftlichen Experiments „Die Untersuchung neuronaler Korrelate während Lebensmittelentscheidungen“ informieren. Bitte lesen Sie sich diese Informationen aufmerksam durch. Wenden Sie sich bitte an uns, falls Sie noch Fragen haben.

Im Rahmen der Studie soll untersucht werden, welche neurologischen Prozesse bei Entscheidungen zwischen zwei Lebensmitteln zugrunde liegen. Das Experiment besteht aus verschiedenen Teilen. Am Tag der Untersuchung werden Sie nach der Besprechung der Instruktionen und der Beantwortung der Verständnisfragen Produkte nach ihrem Geschmack und Gesundheitsprofil am Computer bewerten. Dann folgt ein Experiment im funktionellen Magnetresonanztomographen (fMRT). Anschließend steht Ihnen die Beantwortung von Fragebögen bevor, wonach die Auszahlung umgesetzt wird.

Im Folgenden möchten wir Sie gern über den Ablauf genauer informieren:

- **Instruktionen und Verständnisfragen:** Bitte lesen Sie die nachfolgenden Instruktionen genau durch. Am Tag der Untersuchung stehen Ihnen die Versuchsleiter gern für Rückfragen zur Verfügung. Anschließend erwarten Sie am Untersuchungstag schriftliche Verständnisfragen zu den in den Instruktionen erklärten Aufgaben. Dieser Teil dauert etwa 10 Minuten.

- **Produktbewertung:** In diesem Teil der Untersuchung werden Ihnen am Computer Fotos von einzelnen Lebensmitteln präsentiert. Sie haben dabei die Aufgabe, das Produkt nach Geschmack zu bewerten oder einschätzen, wie gesund das Produkt Ihrer Meinung nach ist. Dieser Teil dauert etwa 30 Minuten.

- **fMRT-Experiment:** Das fMRT-Experiment setzt sich aus drei wiederkehrenden Elementen zusammen (eine detaillierte Beschreibung dieser Elemente finden Sie in den „Instruktionen zu den Aufgaben“):

    1. **Geldaufteilung aus dem „Diktatorspiel“:** Bei diesem Element wird Ihnen die Höhe einer Geldaufteilung aus einem „Diktatorspiel“ angezeigt. Bei einem solchen Diktatorspiel hat ein „Diktator“ die Möglichkeit, einen vorgegebenen Betrag zwischen sich und einem „Empfänger“ aufzuteilen. Dies bedeutet, dass der Diktator die Entscheidung „diktiert“, während der Empfänger keinen Einfluss auf diese Entscheidung nehmen kann. Sie haben dabei die Rolle des Empfängers. Die Diktatoren haben die Geldaufteilungen zu einem früheren Zeitpunkt bestimmt.

Ein Teil der angezeigten Geldaufteilungen wird allerdings nicht von einem Diktator stammen sondern lediglich durch einen Computer generiert sein. Wer die angezeigte Geldaufteilung vorgenommen hat, wird Ihnen entsprechend auf dem Bildschirm angezeigt werden.

2. **Emotionsbewertung:** Bei dem anschließenden Element findet eine Emotionsbewertung statt. Zu diesem Zweck werden Ihnen bei dieser Aufgabe zwei Skalen angezeigt, die unterschiedliche Stimmungen darstellen: Zufriedenheit und Aufregung. Da die Skalen nicht lange angezeigt werden, ist es sehr wichtig, dass Sie nicht lange nachdenken sondern zügig und ehrlich Ihre Gefühlslage in dem entsprechenden Moment angeben.

3. **Lebensmittelentscheidungen:** Bei dieser Aufgabe werden Sie zwei verschiedene Lebensmittelprodukte nebeneinander auf dem Bildschirm sehen. Nun sollen Sie sich entscheiden, welches Lebensmittel Sie lieber essen möchten. Es folgen mehrere Lebensmittelentscheidungen nacheinander.

Genauere Informationen zu den Aufgaben, welche Sie im Scanner bearbeiten sollen, finden Sie in den „Instruktionen zu den Aufgaben".
Das fMRT-Experiment dauert etwa 40 Minuten. Danach wird eine strukturelle Aufnahme Ihres Gehirns erstellt, was ca. 10 Minuten in Anspruch nimmt.

- **Fragebögen:** Im Anschluss an das fMRT-Experiment steht Ihnen das Ausfüllen von Fragebögen zu Ihrer Person am Computer bevor, was ungefähr 20 Minuten dauern wird. Auch bei diesen Fragen gilt, dass richtige und falsche Antworten nicht existieren und, dass Ihre ehrlichen Angaben den Zweck der Untersuchung am besten erfüllen.

- **Auszahlung:** Am Ende des gesamten Experiments, das bis zu 2 Stunden in Anspruch nehmen wird, findet die Auszahlung statt. Dabei erhalten Sie eine Teilnahmevergütung in Höhe von 20 €. Über diese Teilnahmevergütung hinaus wird Ihnen eine der im fMRT-Experiment angezeigten Geldaufteilungen aus dem Diktatorspiel ausgezahlt. Dabei sind lediglich durch Diktatoren getroffene Aufteilungen „auszahlungsrelevant" (d.h. es wird eine der Aufteilungen von einem Diktator ausgezahlt) und die durch einen Computer generierten Runden sind „nicht auszahlungsrelevant" (d.h. werden nicht ausgezahlt). Den letzten Teil der Auszahlung stellt die Umsetzung einer der von Ihnen im fMRT-Experiment getroffenen Lebensmittelentscheidungen dar. Das bedeutet, dass Sie eins der von Ihnen ausgewählten Produkten erhalten und vor Ort verzehren sollen.

Wir wollen, dass es Ihnen während und nach der Untersuchung gut geht. Wenn Ihnen irgendetwas unangenehm ist, Sie etwas nicht verstehen oder genauer wissen wollen, informieren Sie uns bitte und fragen umgehend nach.

Ihre Teilnahme an der Studie ist freiwillig. Sie können jederzeit Ihr Einverständnis zurücknehmen oder den Versuch jederzeit ohne Angabe von Gründen abbrechen. Dadurch wird Ihnen kein Nachteil entstehen. Die gewonnenen Daten werden auf Ihren Wunsch nach Abschluss der Studie vernichtet.

Instruktionen zu den Aufgaben

Produktbewertung

Im Rahmen der Aufgabe der Produktbewertung am Computer werden Ihnen Bilder von Lebensmitteln gezeigt. Auf einer Skala, die unter dem Lebensmittel zu sehen ist (siehe folgende Abbildung), können Sie die Lebensmittel mit der Maus bewerten. Die Lebensmittel werden von Ihnen sowohl im Hinblick auf den Geschmack als auch im Hinblick auf die Gesundheit in Blöcken nacheinander bewertet. Welcher der Bewertungsblöcke (Geschmack / Gesundheit) als erstes abgefragt wird, ist zufällig, wird aber auf dem Bildschirm einleitend angezeigt.
Falls Sie das angezeigte Produkt nicht kennen, so nehmen Sie dennoch so gut Sie können eine Einschätzung vor. Bitte schauen Sie sich jedes Produkt genau an, aber treffen Sie Ihre Entscheidungen zügig und denken Sie nicht zu lange nach. Dieser Teil dauert etwa 30 Minuten. Bitte geben Sie Ihre Meinung so zutreffend wie möglich ab, denn Ihre Bewertungen bestimmen den weiteren Verlauf des Experiments.

Hier je ein Beispiel zur Verdeutlichung:

Gesundheit:

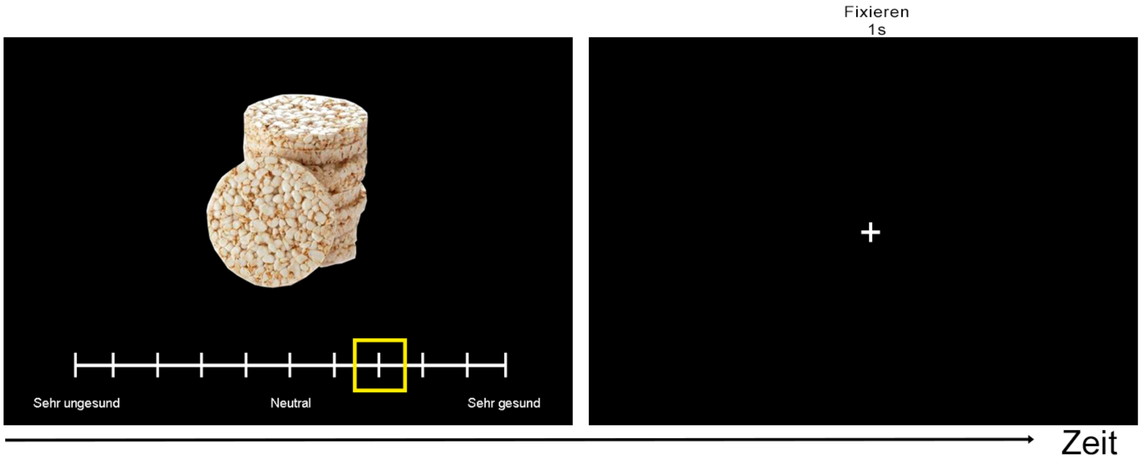

Geschmack:

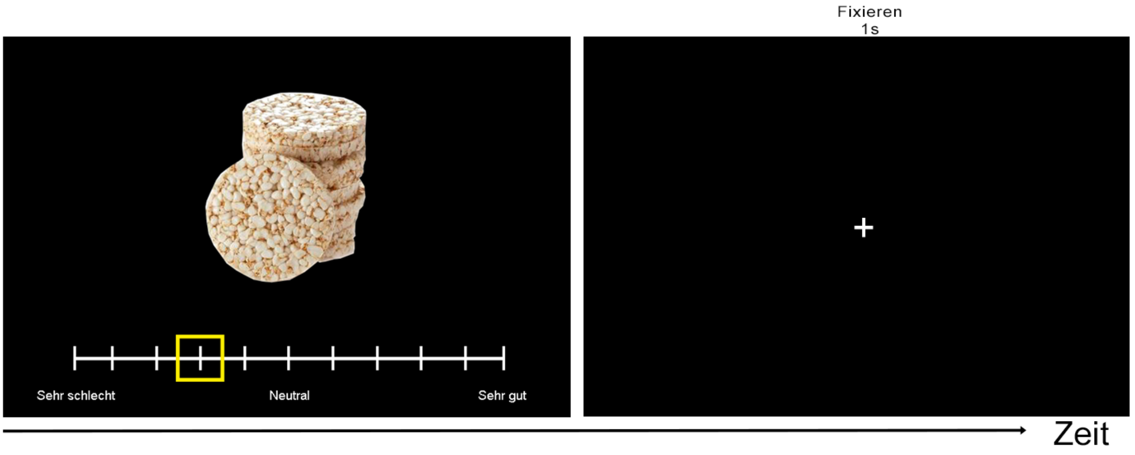

FMRT-Experiment

Das anschließende fMRT-Experiment dauert etwa 40 Minuten und wird auf den folgenden Seiten genauer beschrieben. Wie bereits geschildert, setzt es sich aus drei wiederkehrenden Elementen zusammen (vgl. Abbildung):

1. Geldaufteilung aus dem Diktatorspiel
2. Emotionsbewertung
3. Lebensmittelentscheidungen

Diese zwei Elemente wurden bereits in der „Information zur Teilnahme" thematisiert und werden nachfolgend detailliert beschrieben. Die folgende Abbildung zeigt den Ablauf im Scanner. Zwischen den Elementen werden sogenannte „Fixationskreuze" (weißes Kreuz) angezeigt – bitte schauen Sie diese beim Warten einfach an.

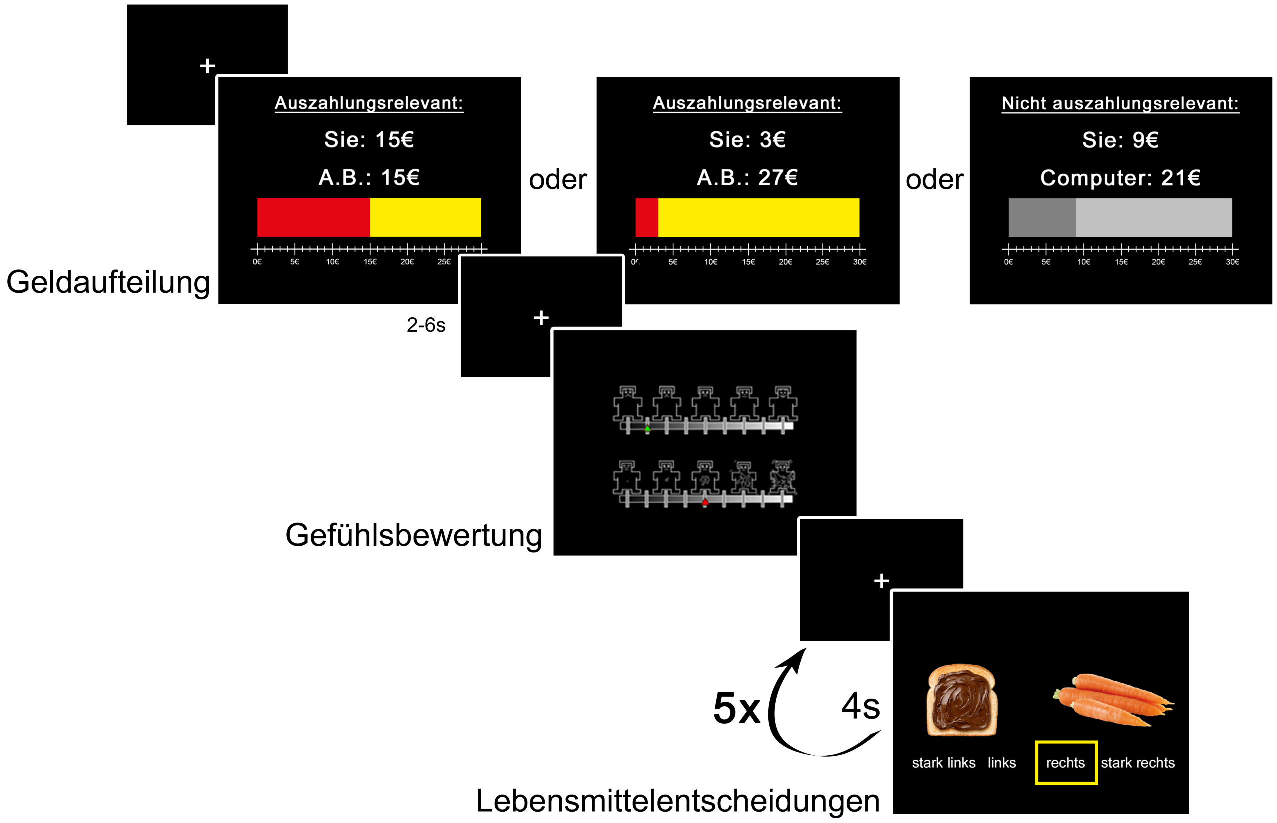

1. Geldaufteilung aus dem „Diktatorspiel“

Aufteilung durch einen Diktator

Zu Beginn jedes Durchganges werden Ihnen Geldaufteilungen aus einem Diktatorspiel angezeigt. Dabei hat eine andere Versuchsperson, der „Diktator“, **30 €** zwischen sich und Ihnen aufgeteilt. Dies bedeutet, dass der Diktator die Entscheidung „diktiert“, während der Empfänger keinen Einfluss auf diese Entscheidung nehmen kann. Dies geschah am 25.02.2016 in einem anderen Labor der Universität Bonn, dem BonnEconLab. Sie sind der „Empfänger“ der getroffenen Aufteilung und haben keinen Einfluss auf diese.

Die folgende Abbildung stellt ein Beispiel dar, wie die Geldaufteilungen Ihnen in diesem Experiment angezeigt werden.

Auszahlungsrelevante Aufteilung durch einen Diktator:
Abbildung A)

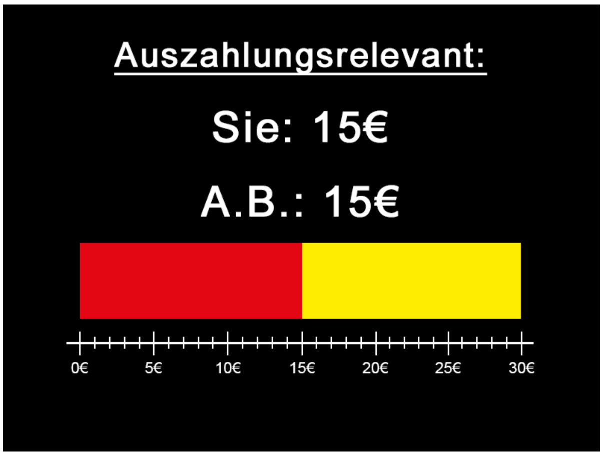

Die 40 Diktatoren wurden darüber informiert, dass Sie als Empfänger auch an diesem Diktatorspiel teilnehmen werden. Anschließend trugen die Diktatoren die Initialen ihres Vor- und Nachnamens ein. Diese Initialen werden Ihnen mit der entsprechenden Aufteilung des Diktators im Experiment angezeigt (s. Abb. A).

Im Experiment wird es mehrere Diktatoren geben, d.h. es erwarten Sie ähnliche Abbildungen wie in Abb. A). Alle Entscheidungen der Diktatoren sind potentiell relevant für Ihre Auszahlung, deswegen „Auszahlungsrelevant“ (d.h. es wird eine der Aufteilungen von einem Diktator ausgezahlt) (Abb. A). Aber (!) nur eine dieser Aufteilungen wird Ihnen ausgezahlt. Welche das sein wird, erfahren Sie erst nach dem Experiment. Bedenken Sie daher während des Experiments, dass jede der Ihnen angezeigten Aufteilungen durch einen Diktator potentiell die auszahlungsrelevante sein kann!

Jeder Diktator teilte das Geld während seines Experiments nur ein einziges Mal auf. Die Diktatoren wussten aber, dass die Auszahlung einen bestimmten Empfänger (Sie und Ihre Auszahlung also) betrifft. Die Diktatoren wurden darauf hingewiesen, dass Ihr Experiment etwa 2,5 Stunden dauert, während deren Untersuchung nur etwa 30 Minuten in Anspruch genommen hat.

Die Diktatoren 1-40 hatten dann die Möglichkeit, sich zwischen zwei alternativen Aufteilungen der 30€ (Alternativen A oder B) zu entscheiden:

Diktatoren	Alternative A		Alternative B
Diktatoren 1-10	Sie 15€ / Diktator 15€	oder	Sie 6 € / Diktator 24 €
Diktatoren 11-20	Sie 14€ / Diktator 16€	oder	Sie 5 € / Diktator 25 €
Diktatoren 21-30	Sie 13€ / Diktator 17€	oder	Sie 4 € / Diktator 26 €
Diktatoren 31-40	Sie 12€ / Diktator 18€	oder	Sie 3 € / Diktator 27 €

Das bedeutet, dass wenn Ihnen im Experiment angezeigt wird, dass ein Diktator Ihnen 13€ und sich selbst 17€ zugeteilt hat, dann wissen Sie, dass dieser die Wahl zwischen dieser Alternative A und der Alternative B in Höhe von Sie 4€ / Diktator 26€ hatte.

Danach war das Experiment der Diktatoren vom 25.02.2016 zu Ende.

Aufteilung durch einen Computer
Ein Teil der angezeigten Geldaufteilungen wird aber nicht von einem Diktator stammen. Dieser Teil wird durch einen Computer generiert und ist nicht relevant für die Auszahlung, also „Nicht auszahlungsrelevant" (d.h. werden nicht ausgezahlt) (s. Abb. B). Die Aufteilung eines Computers wird Ihnen in keinem Fall ausgezahlt. Hat der Computer die Aufteilung vorgenommen, wird auf dem Bildschirm im Experiment immer die Bezeichnung „Computer" (Abb. B) angezeigt und der Balken der Geldaufteilung grau dargestellt. Hat ein Diktator die Aufteilung übernommen, werden auf dem Bildschirm immer die Initialen des entsprechenden Diktators angezeigt (Abb. A).

Beispiel für eine nicht auszahlungsrelevante Aufteilung durch einen Computer:
Abbildung B)

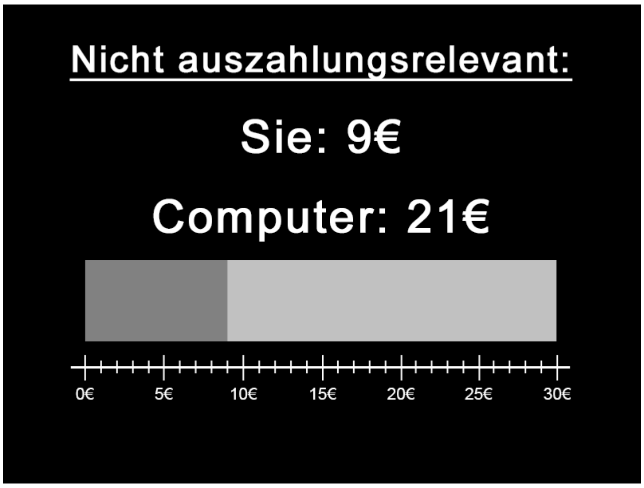

Während die Geldaufteilungen aus dem Diktatorspiel angezeigt werden, brauchen Sie nichts zu unternehmen oder zu drücken. Bitte schauen Sie die Entscheidung einfach an und achten Sie bitte auf die Höhe der Geldaufteilung und darauf, ob diese durch einen Diktator oder den Computer vorgenommen wurde.

2. Emotionsbewertung

Nach der Darstellung der Geldaufteilung findet eine Bewertung Ihrer Emotionen statt. Dabei bitten wir Sie, Ihre aktuelle Stimmungslage durch die Auswahl von Symbolen auszudrücken. Es gibt keine richtigen oder falschen Antworten. Bei dieser Aufgabe werden Ihnen zwei Skalen angezeigt, die unterschiedliche Stimmungen darstellen: Zufriedenheit und Aufregung.

Skala I: Zufriedenheit

Sie sehen hier, dass jede Figur entlang der Skala zwischen fröhlich und traurig variiert. Am einen Ende fühlen Sie sich vollkommen fröhlich, erfreut, zufrieden, glücklich, hoffnungsvoll. Am anderen Ende der Skala sind Sie vollkommen traurig, genervt, unzufrieden, melancholisch, verzweifelt, gelangweilt.

traurig
genervt
unzufrieden
melancholisch
verzweifelt
gelangweilt

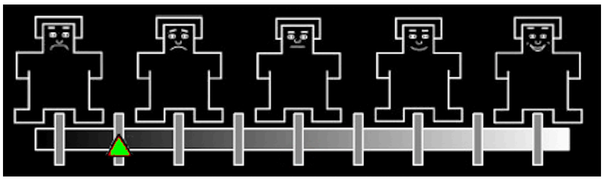

fröhlich
erfreut
zufrieden
glücklich
hoffnungsvoll

Skala II: Aufregung

Sie sehen hier, dass jede Figur entlang der Skala zwischen Aufregung und Ruhe variiert. Am einen Ende fühlen Sie sich vollkommen angeregt, aufgeregt, hektisch, nervös, hellwach oder aufgerüttelt. Am anderen Ende der Skala sind Sie vollkommen entspannt, ruhig, träge, benommen, müde, unaufgeregt.
Sie können auf der Schiebeskala auch Zwischenstufen wählen.

entspannt
ruhig
träge
benommen
müde
unaufgeregt

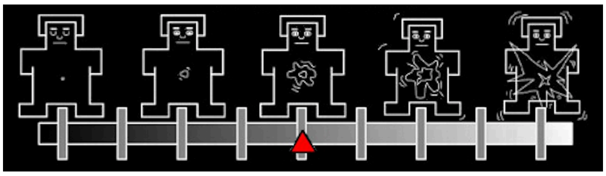

angeregt
aufgeregt
hektisch
nervös
hellwach
aufgerüttelt

Im fMRT-Experiment wird das rote Dreieck (der Cursor) zufällig an einer Stelle der ersten Skala auftauchen. Die Auswahl im Scanner treffen Sie, indem Sie die Tasten auf den MRT-Griffen betätigen. Dabei gilt folgende Tastenbelegung:

Betätigen Sie den linken und rechten Zeigefinger, um den Cursor entsprechend nach links und rechts zu verschieben. Sie bestätigen die Auswahl mit dem Knopf am rechten Daumen. Dabei färbt sich der Cursor grün als Zeichen dafür, dass Ihre Eingabe registriert wurde. Wiederholen Sie auch bei der Skala II den Vorgang auf die gleiche Weise. Da die Skalen lediglich 6 Sekunden lang angezeigt werden, ist es sehr wichtig, dass Sie ohne lang nachzudenken zügig und ehrlich Ihre Gefühlslage in dem entsprechenden Moment angeben!

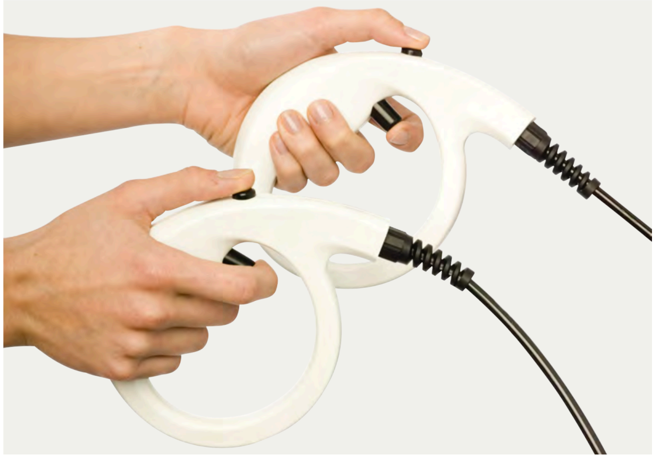

3. Lebensmittelentscheidungen

Anschließend haben Sie die Wahl zwischen zwei verschiedenen Lebensmitteln. Sie haben dabei die Möglichkeit anzugeben, ob Sie das entsprechende Produkt eher stark oder weniger stark präferieren (stark links/links/rechts/stark rechts). Eine der von Ihnen dabei getroffenen Entscheidungen wird Ihnen am Ende des Experiments ausgehändigt und soll tatsächlich vor Ort verzehrt werden. Achten Sie daher darauf, in jeder Runde nur Produkte auszuwählen, die Sie tatsächlich gern nach dem Experiment essen möchten. Bei den Entscheidungsaufgaben sollen Sie ein übergeordnetes Ziel verfolgen. Das Ziel in jeder Entscheidungsrunde wird es sein, sich **möglichst gesund** zu ernähren. Für Ihre Entscheidung haben Sie **lediglich bis zu 4 Sekunden** Zeit. Falls Sie mehr als 4 Sekunden benötigen, wird Ihre Antwort nicht gewertet. Nach jeder Wahl zwischen zwei Produkten, werden Sie ein weißes Kreuz in der Mitte des Bildschirmes sehen. Bitte fixieren Sie das Kreuz und bleiben ruhig liegen, bis Sie die nächste Wahl treffen können.

Hier ein Beispiel für zwei auf einander folgende Entscheidungsrunden:

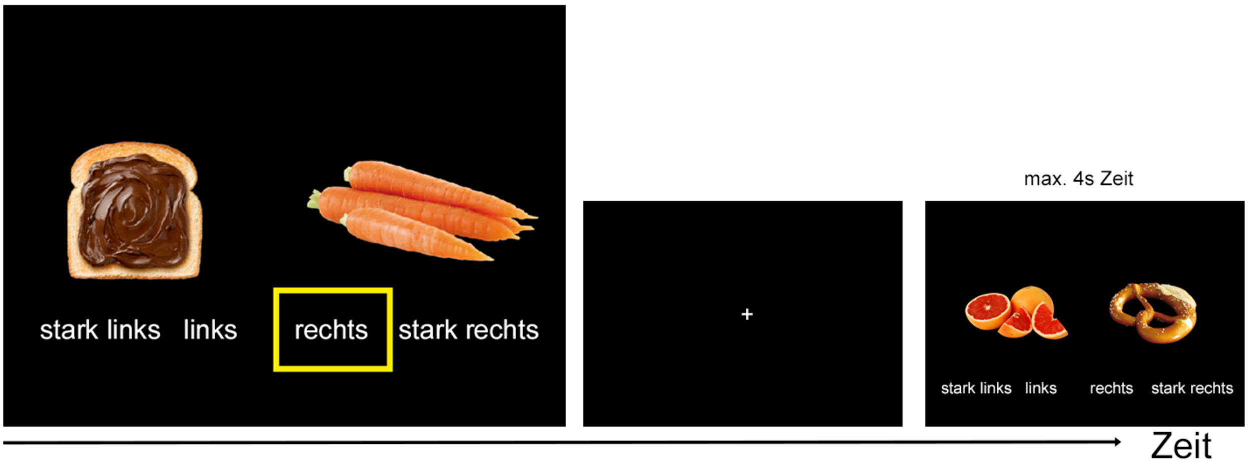

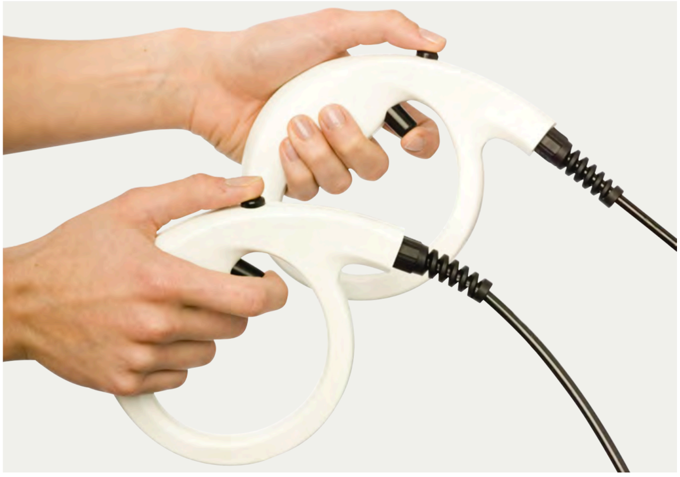

Die Auswahl im Scanner treffen Sie bei dieser Aufgabe, indem Sie die Tasten auf den MRT-Griffen betätigen. Dabei gilt folgende Tastenbelegung:
Für Ihre Entscheidung betätigen Sie bitte den linken Zeigefinger für das linke Produkt (links) bzw. den rechten Zeigefinger für das rechte Produkt (rechts). Wenn Sie eine starke Präferenz für das linke Produkt haben, betätigen Sie bitte den linken Daumen (stark links) bzw. den rechten Daumen bei einer starken Präferenz für das rechte Produkt (stark rechts). Sobald Sie einen Tastendruck tätigen, wird die Entscheidung registriert, was durch einen gelben Rahmen um das entsprechende Produkt gekennzeichnet wird (siehe Abbildung). Das Experiment geht dann automatisch mit dem Fixationskreuz weiter.

Es folgen mehrere Lebensmittelentscheidungen nacheinander, bevor der nächste Durchgang mit dem Diktatorspiel von vorne startet.

Nach dem 40-minütigen fMRT-Experiment wird eine strukturelle Aufnahme Ihres Gehirns erstellt, was ca. 10 Minuten in Anspruch nimmt. Während der strukturellen Messung bearbeiten Sie keine weiteren Aufgaben, Sie dürfen dabei die Augen schließen. **Bitte achten Sie darauf, Ihren Kopf während des fMRT-Experiments und der strukturellen Aufnahme nicht zu bewegen, um eine gute Bildqualität sicherzustellen! Das ist sehr wichtig für die weitere Auswertung der Daten.**

Vielen Dank für Ihre Teilnahme!
