# Supplementary material for: Do Disadvantageous Social Contexts Influence Food Choice? Evidence From Three Laboratory Experiments
Source: Front Psychol. 2020 Nov 6;11:575170. doi: 10.3389/fpsyg.2020.575170 (PMC7677191; doi:10.3389/fpsyg.2020.575170)
Supplement: Supplementary file 5 [file Data_Sheet_5.pdf]

Information (I.) and instructions (II.) provided to the participants in the Cyberball Game experiment

As the original instructions were in German, we provide here an English translation of the same. We show the original figures presented to the participants with English translation of the words appearing in those figures below the figures.

I. Information for participants

Dear participant,

in the following we would like to inform you about the course of the scientific study "The investigation of neuronal correlates of food decision making". Please read this information carefully and contact us if you have any further questions.

The aim of this study is to investigate the neurological processes underlying decisions between two food items. The experiment consists of different parts. After reading the instructions and answering the comprehension questions, you will evaluate different food items on the computer in terms of how tasty and how healthy you think they are. This is followed by an experiment in a functional magnetic resonance imaging (fMRI) scanner. In the end, you will be asked to fill in some questionnaires, after which you will be reimbursed for your participation.
In the following we would like to inform you about the procedure in more detail:

Instructions and comprehension questions: Please read the following instructions carefully. If you have any questions, please do not hesitate to contact the investigators. Afterwards, you can expect written comprehension questions regarding the tasks explained in the instructions. This part takes about 20 minutes.

Food rating task: In this part of the experiment you will be presented with images of individual food items on a computer screen. Your task is to evaluate the presented food items in terms of how tasty and how healthy you think these items are. This part takes about 30 minutes.

fMRI experiment: The fMRI experiment is composed of three recurring elements (a detailed description of these elements can be found in the “Instructions for the tasks”):

1. **Cyberball Game:** At the beginning of each round you will play an online ball-tossing game together with two other players. The ID's of the other players are shown on the screen and an arrow shows you your position (in the middle of the screen). You have to press a button to throw the ball either to the player on your right, or to the player on your left. You can throw the ball to any player, but your decision should be made as quickly as possible. If you do not decide quickly enough, the ball will be thrown randomly to one of the players. If you throw the ball every time the ball is thrown to you (active playing), you will receive an additional payoff at the end of the experiment.
2. **Emotion ratings:** In the following element an emotion rating takes place. For this purpose, you will be shown two scales representing different aspects of emotions: valence and arousal. Since the scales are not displayed for a long time, it is very important that you do not think about it for a long time, but rather quickly and honestly state your emotional state at the appropriate moment.
3. **Food choices:** In this task you will see two different food products next to each other on the screen. You have to decide which food you would rather eat at that moment. Several food choices follow one after the other.

For more detailed information about the tasks you are to perform in the scanner, please refer to the “Instructions for the tasks” (which you will receive at Life and Brain). The fMRI experiment takes about 40 minutes. Afterwards, a structural image of your brain is taken; this will take about 10 minutes.

Questionnaires: After the fMRI experiment, you will be asked to fill in questionnaires on the computer. This part will take about 20 minutes. These questions are also based on the principle that correct and incorrect answers do not exist and that your honest responses best serve the purpose of the investigation.

Reimbursement: At the end of the whole experiment, which will take up to 2.5 hours, you will be reimbursed for your participation. You will receive a participation fee of €20. Beyond this fee you will receive an additional payment of up to €5, depending on your

performance in the Cyberball Game and on your ability to hold still during scanning. The last part of the payment is the implementation of one of the food decisions you make in the fMRI experiment. This means that you will receive one of the food items you selected. If you wish, you can also get a CD with a copy of the structural MRI image of your brain.

We want you to feel good during and after the experiment. If you feel uncomfortable about something, if you do not understand something or want to know more about it, please inform us and ask immediately.

Your participation in the study is voluntary. You can withdraw your consent at any time or stop the experiment at any time without giving any reasons. You will not suffer any disadvantage as a result. The data obtained will be destroyed at your request after completion of the study.

II. Instructions for the tasks

Food rating task

In the food rating task, you will be shown images of food items on a computer screen. You should rate each food item using the mouse and the scale displayed under the food item (see the following figure). You will rate food items in two blocks, once in terms of taste and once in terms of health. Which of the evaluation blocks (taste / health) is queried first is random, but it will be indicated on the screen before the start of the block. If you do not know the displayed food item, please make an assessment as best as you can. Please look at each food item carefully but make your decisions quickly and do not think too long. When rating the food items, please make use of the **full scale** (from min. 1 to max. 7). This part of the experiment takes about 20–30 minutes.

Here is an example from each block for clarification:

Health:

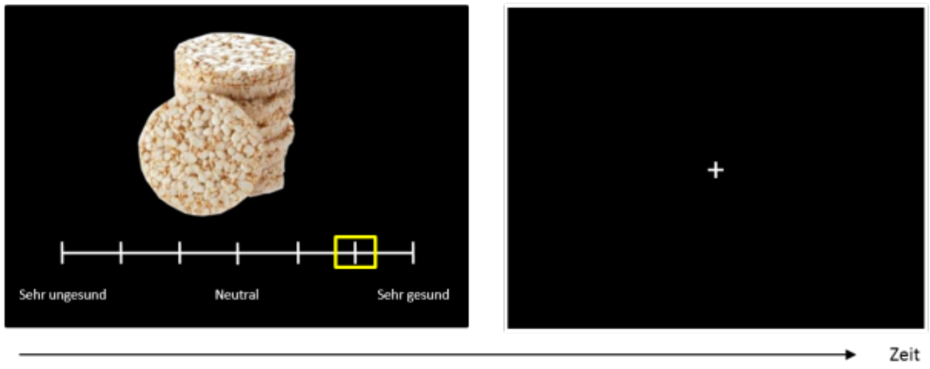

Sehr ungesund = very unhealthy; Sehr gesund = very healthy; Zeit = time.

Taste:

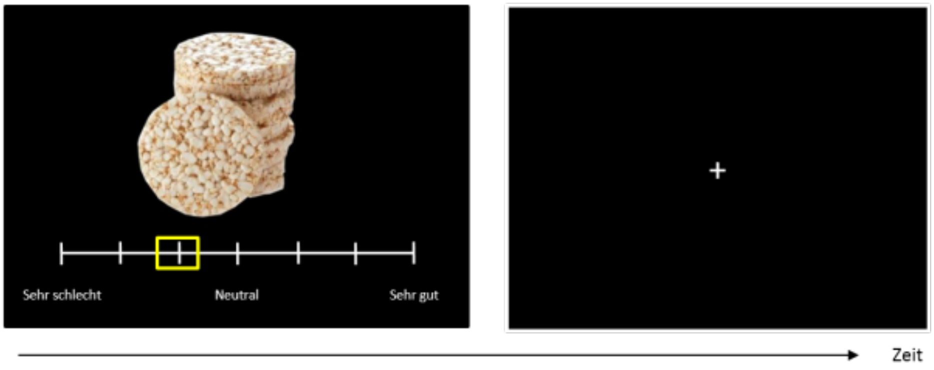

Sehr schlecht = very bad; Sehr gut = very good; Zeit = time.

<u>fMRI experiment</u>

The fMRI experiment lasts approximately 40–50 minutes and is explained below in more detail. As already described, the experiment consists of three recurring elements (see figure below):

1. **Cyberball Game**
2. **Emotions ratings**
3. **Food choices**

These three elements have already been mentioned in the “Information for participants” and will now be explained in more detail. The following figure displays the timeline of the experiment in the scanner. In-between the different elements of the experiment you will be shown “fixation crosses”, please just look at them while waiting.

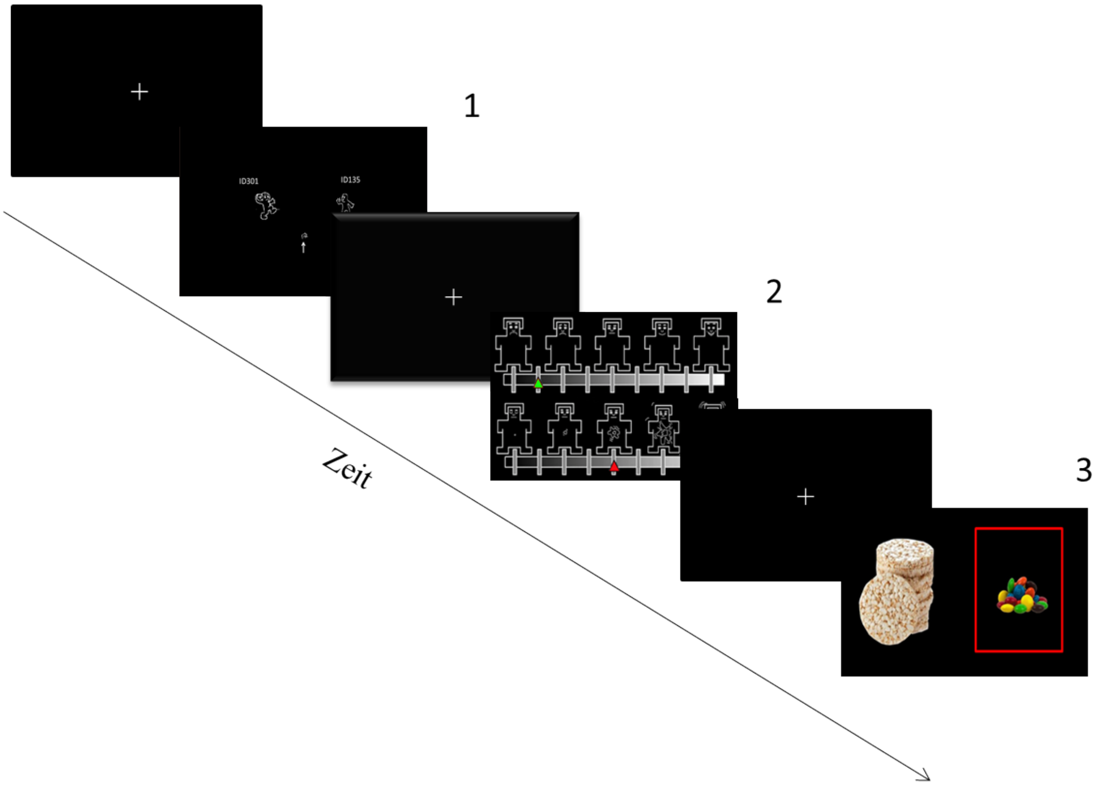

Zeit = time

1. Cyberball Game

At the beginning of each round you will play an online ball-tossing game with two other players. The other players' will be indicated with IDs, whereas your position will be indicated with an arrow (in the middle of the screen). You need to press a button to toss the ball to either the player on your left or the player on your right side. You can throw the ball to any player, but your decision should be made as soon as possible. If you do not decide quickly enough, the ball will be thrown randomly to one of the players. If you throw the ball every time the ball is thrown to you (active playing), you will receive an additional payout at the end of the experiment. Here you can see an exemplary representation of the game:

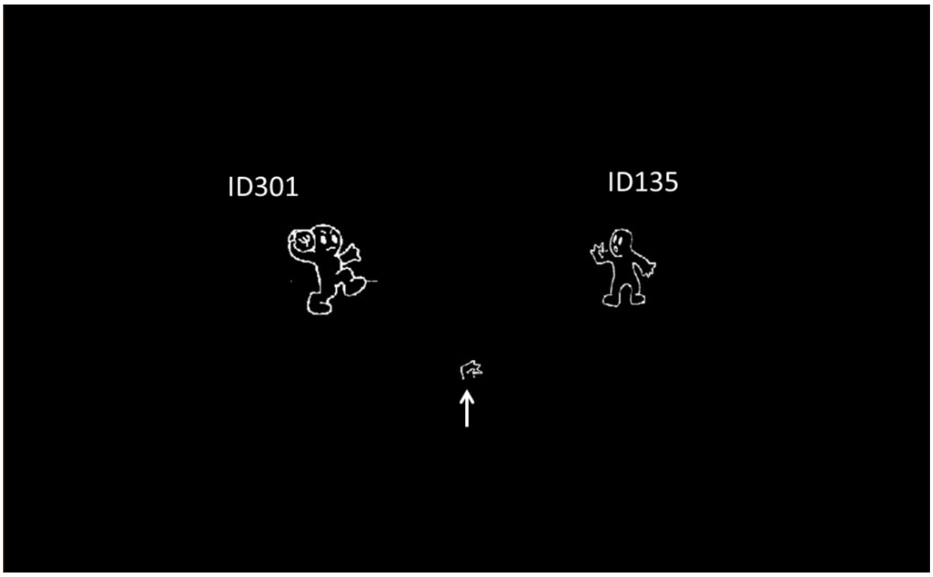

2. Emotion ratings

After the Cyberball Game, an emotion rating task follows. In this task, we ask you to express your current emotional state by selecting symbols. There are no right or wrong answers.

During this task you will be shown two scales representing different aspects of emotions: valence and arousal. Each of the scales has 9 different values. On the far right and the far left of the scale are the extremes. Try to memorize the meaning of the two scales and the respective extremes now, because during the fMRI experiment only the symbols will be shown on the screen.

Scale I: “unhappy – happy“

unzufrieden (*unsatisfied*)
unglücklich (*unhappy*)
genervt (*annoyed*)
verzweifelt (*desperate*)
schwermütig (*melancholic*)

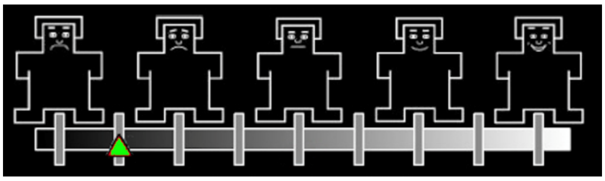

zufrieden (*satisfied*)
glücklich (*happy*)
erfreut (*delighted*)
hoffnungsvoll (*hopeful*)
ausgeglichen (*balanced*)

Scale II: “calm – aroused”

entspannt (*relaxed*)
unerregt (*unexcited*)
ruhig (*calm*)

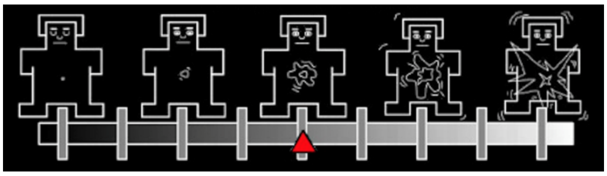

rasend (*furious*)
erregt (*agitated*)
aufgeregt (*aroused*)

In the fMRI experiment, the red triangle (the cursor) will appear randomly at a position on the first scale. You will rate your emotions by pressing the buttons on the MRI response grips. The following key assignment applies:

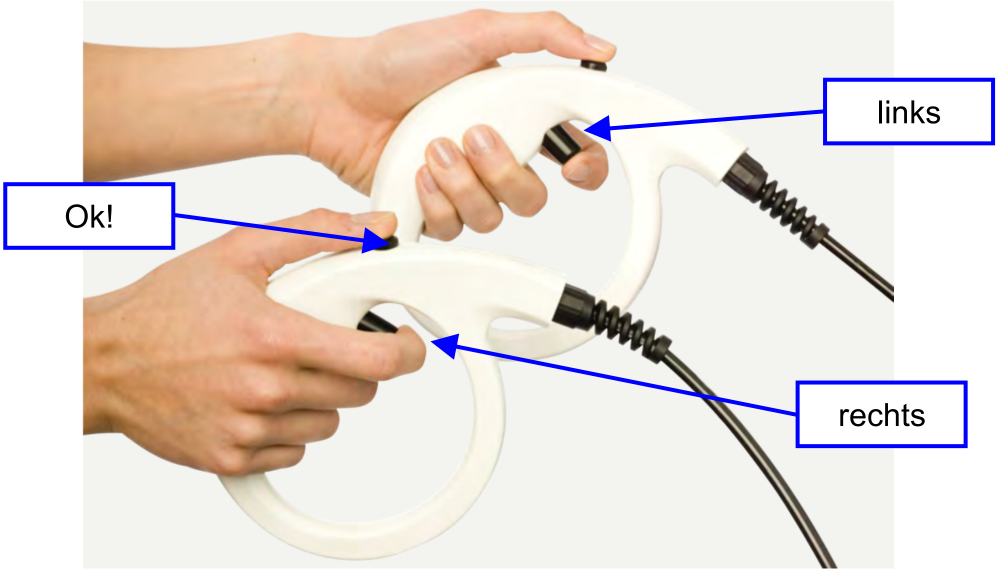

Links = left; rechts = right

Use the left and right index finger to move the cursor left and right accordingly. Confirm the selection with the button on your right thumb. The cursor will turn green to indicate that your entry has been registered. Repeat the process in the same way for scale II. Since the scales are only displayed for 6 seconds, it is very important that you state your emotional state at the appropriate moment quickly and honestly without thinking about it for too long!

3. Food choices

Following the emotion rating task, you will see two different food items next to each other on the screen. You should decide which food you would prefer to eat at that moment. Since, according to the self-disclosure, you are making an effort to eat a balanced and healthy diet, we ask you to make choices that correspond to a healthy and balanced diet as much as possible. You have up to 4 seconds to make your decision, so please react quickly. If you decide earlier, the experiment will continue immediately. If you need more than 4 seconds to respond, your answer will not be evaluated.

Here is an example of two successive rounds of decisions:

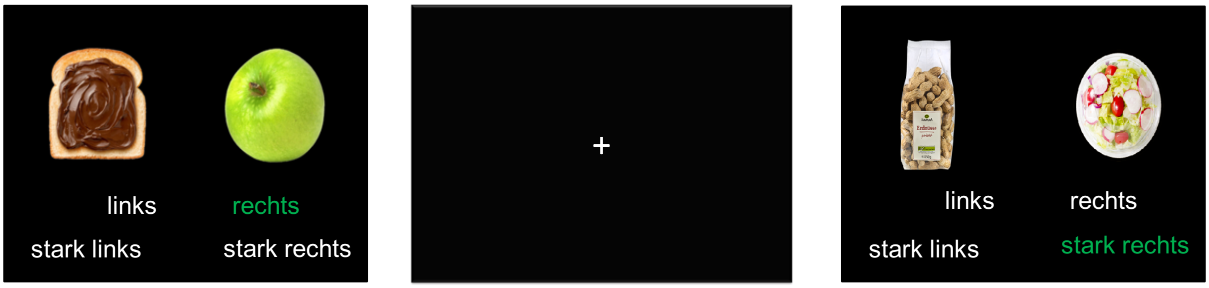

Links = left; stark links = strong left; rechts = right; stark rechts = strong right

In this task, just like in the emotion rating task, you should indicate your preference by pressing the buttons on the MRI response grips. The following key assignment applies:

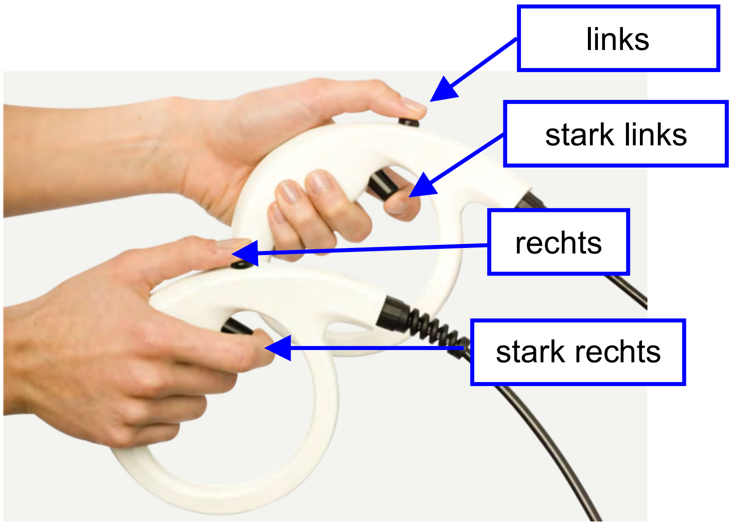

Links = left; stark links = strong left; rechts = right;
stark rechts = strong right

You can choose between two preference strengths for your chosen food item: strong preference (index finger) left/right and weaker preference (thumb) left/right. To make your choice, please use your **left index finger, or left thumb** for the left product, or **your right index finger or right thumb** for the right product. As soon as you press the left or right index finger or thumb, the decision is registered, and this is indicated by a change of color from white to green.

At the end of the whole experiment, one trial will be randomly selected and we will give you the food item that you selected in that trial. Therefore, be careful to select only food items that you really want to eat in **every trial.** Several food choices will follow one after the other before the next round with the Cyberball Game starts all over again.

After the 40-minute fMRI experiment, a structural image of your brain is taken; this takes about 10 minutes. During the structural measurement you will not perform any tasks and you may close your eyes.

Please take care not to move your head during the fMRI experiment and during the acquisition of the structural image to ensure good image quality! This is very important for the further evaluation of the data.

Thank you very much for your participation!
