# Supplementary material for: Do Disadvantageous Social Contexts Influence Food Choice? Evidence From Three Laboratory Experiments
Source: Front Psychol. 2020 Nov 6;11:575170. doi: 10.3389/fpsyg.2020.575170 (PMC7677191; doi:10.3389/fpsyg.2020.575170)
Supplement: Supplementary file 6 [file Data_Sheet_6.pdf]

Information zur Teilnahme an der wissenschaftlichen Studie

„Die Untersuchung neuronaler Korrelate während Lebensmittelentscheidungen“

Verantwortlicher Studienleiter: Prof. Dr. med. Bernd Weber

Department of NeuroCognition, Life & Brain Center und Klink für Epileptologie,
Universitätsklinikum Bonn
Acting Director
Center for Economics and Neuroscience
Nachtigallenweg 86
53127 Bonn
Tel.: ++49 228 73-8290d
Tel.: ++49 228 73-8299

Department of Epileptology

Head - NeuroCognition | Imaging
Life&Brain Center

Sigmund-Freud-Str. 25
53127 Bonn
Tel.: ++49 228 6885-262
Fax: ++49 228 6885-261
----
www.lifeandbrain.com
www.neuroeconomics-bonn.org

Liebe/r an der Studie Interessierte/r,

im Folgenden möchten wir Sie über den Ablauf der wissenschaftlichen Studie „Die Untersuchung neuronaler Korrelate während Lebensmittelentscheidungen“ informieren. Bitte lesen Sie sich diese Informationen aufmerksam durch. Wenden Sie sich bitte an uns, falls Sie noch Fragen haben.

Im Rahmen der Studie soll untersucht werden, welche neurologischen Prozesse bei Entscheidungen zwischen zwei Lebensmitteln zugrunde liegen. Das Experiment besteht aus verschiedenen Teilen. Nach dem Lesen der Instruktionen und der Beantwortung der Verständnisfragen werden Sie Produkte nach ihrem Geschmack und Gesundheitsprofil am Computer bewerten. Dann folgt ein Experiment im funktionellen Magnetresonanztomographen (fMRT). Anschließend steht Ihnen die Beantwortung von Fragebögen bevor, wonach die Auszahlung umgesetzt wird.

Im Folgenden möchten wir Sie gern über den Ablauf genauer informieren:

- **Instruktionen und Verständnisfragen:** Bitte lesen Sie die nachfolgenden Instruktionen genau durch. Bei Fragen stehen Ihnen die Versuchsleiter gern zur Verfügung. Anschließend erwarten Sie schriftliche Verständnisfragen zu den in den Instruktionen erklärten Aufgaben. Dieser Teil dauert etwa 5-10 Minuten.

- **Produktbewertung:** In diesem Teil der Untersuchung werden Ihnen am Computer Fotos von einzelnen Lebensmitteln präsentiert. Sie haben dabei die Aufgabe, das Produkt nach Geschmack zu bewerten oder einschätzen, wie

gesund das Produkt Ihrer Meinung nach ist. Dieser Teil dauert etwa 20-30 Minuten.

- **fMRT-Experiment:** Das fMRT-Experiment setzt sich aus drei wiederkehrenden Elementen zusammen (eine detaillierte Beschreibung dieser Elemente finden Sie in den „Instruktionen zu den Aufgaben“)

1. **Cyberball- Aufgabe:** Am Anfang jeder Runde spielen Sie zusammen mit zwei anderen Spielern ein Online Ballspiel. Die ID’s der anderen Spieler werden im Bildschirm angezeigt, ein Pfeil zeigt Ihnen Ihre Position an (in der Mitte des Bildschirms). Sie müssen einen Knopf drücken um den Ball entweder zu dem Spieler auf Ihrer rechten Seite, oder dem auf der linken Seite zu werfen. Sie können den Ball zu einem beliebigen Spieler werfen, Ihre Entscheidung sollte jedoch so schnell wie möglich erfolgen. Wenn Sie sich nicht schnell genug entscheiden, wird der Ball zufällig zu einem Ihrer Mitspieler geworfen. Wenn Sie den Ball in zu dem angewiesenem Mitspieler werfen, erhalten Sie am Ende des Experiments eine zusätzliche Auszahlung.
2. **Emotionsratings:** Bei dem anschließenden Element findet ein Emotionsrating statt. Zu diesem Zweck werden Ihnen bei dieser Aufgabe zwei Skalen angezeigt, die unterschiedliche Stimmungen darstellen: Zufriedenheit und Aufregung. Da die Skalen nicht lange angezeigt werden, ist es sehr wichtig, dass Sie nicht lange nachdenken sondern zügig und ehrlich Ihre Gefühlslage in dem entsprechenden Moment angeben.
3. **Lebensmittelentscheidungen:** Bei dieser Aufgabe werden Sie zwei verschiedene Lebensmittelprodukte nebeneinander auf dem Bildschirm sehen.

Nun sollen Sie sich entscheiden, welches Lebensmittel Sie lieber essen möchten. Es folgen mehrere Lebensmittelentscheidungen nacheinander.

Genauere Informationen zu den Aufgaben, welche Sie im Scanner bearbeiten sollen, finden Sie in den „Instruktionen zu den Aufgaben“ (Sie bekommen das in Life and Brain).
Das fMRT-Experiment dauert etwa 40-50 Minuten. Danach wird eine strukturelle Aufnahme Ihres Gehirns erstellt, was ca. 10 Minuten in Anspruch nimmt. Für die MRT Aufnahmen ist es wichtig, dass sie sich während dem Scannen nicht bewegen. Wenn es Ihnen gelingt sich nicht zu bewegen, bekommen Sie am Ende der Studie einen zusätzlichen Geldbetrag ausgezahlt.

- **Fragebögen**: Vor und nach dem fMRT-Experiment steht Ihnen das Ausfüllen von Fragebögen zu Ihrer Person am Computer bevor, was ungefähr 20 Minuten dauern wird. Auch bei diesen Fragen gilt, dass richtige und falsche Antworten nicht existieren und, dass Ihre ehrlichen Angaben den Zweck der Untersuchung am besten erfüllen.
- **Auszahlung:** Am Ende des gesamten Experiments, das bis zu 2 Stunden in Anspruch nehmen wird, findet die Auszahlung statt. Dabei erhalten Sie eine Teilnahmevergütung in Höhe von 20 €. Über diese Teilnahmevergütung hinaus erhalten Sie eine zusätzliche Auszahlung von bis zu 5€, die von Ihrer Leistung aus der Cyberball- Aufgabe abhängt und davon, ob es Ihnen gelungen ist sich nicht zu bewegen während dem Scan. Den letzten Teil der Auszahlung stellt die Umsetzung einer der von Ihnen im fMRT-Experiment getroffenen Lebensmittelentscheidungen dar. Das bedeutet, dass Sie eins der von Ihnen

ausgewählten Produkte erhalten. Es besteht die Möglichkeit, die strukturelle MRT-Aufnahme Ihres Gehirns auf einer CD kopiert zu bekommen, wenn Sie dies wünschen.

Wir wollen, dass es Ihnen während und nach der Untersuchung gut geht. Wenn Ihnen irgendetwas unangenehm ist, Sie etwas nicht verstehen oder genauer wissen wollen, informieren Sie uns bitte und fragen umgehend nach.

Ihre Teilnahme an der Studie ist freiwillig. Sie können jederzeit Ihr Einverständnis zurücknehmen oder den Versuch jederzeit ohne Angabe von Gründen abbrechen. Dadurch wird Ihnen kein Nachteil entstehen. Die gewonnenen Daten werden auf Ihren Wunsch nach Abschluss der Studie vernichtet.

____________________________ ____________________________

Unterschrift Proband/-in Ort, Datum

____________________________ ____________________________

Unterschrift des Untersuchenden Ort, Datum

Instruktionen zu den Aufgaben

<u>Produktbewertung</u>

Im Rahmen der Aufgabe der Produktbewertung am Computer werden Ihnen Bilder von Lebensmitteln gezeigt. Auf einer Skala, die unter dem Lebensmittel zu sehen ist (siehe folgende Abbildung), können Sie die Lebensmittel mit der Maus bewerten. Die Lebensmittel werden von Ihnen sowohl im Hinblick auf den Geschmack als auch im Hinblick auf die Gesundheit in Blöcken nacheinander bewertet. Welcher der Bewertungsblöcke (Geschmack / Gesundheit) als erstes abgefragt wird, ist zufällig, wird aber auf dem Bildschirm einleitend angezeigt.
Falls Sie das angezeigte Produkt nicht kennen, so nehmen Sie dennoch so gut Sie können eine Einschätzung vor. Bitte schauen Sie sich jedes Produkt genau an, aber treffen Sie Ihre Entscheidungen zügig und denken Sie nicht zu lange nach. Bitte verwenden Sie bei der Beantwortung der Fragen **die <u>gesamte Breite</u>** der jeweiligen Skala (von min. 1 bis max. 7). Dieser Teil dauert etwa 20-30 Minuten.

Hier je ein Beispiel zur Verdeutlichung:

Gesundheit:

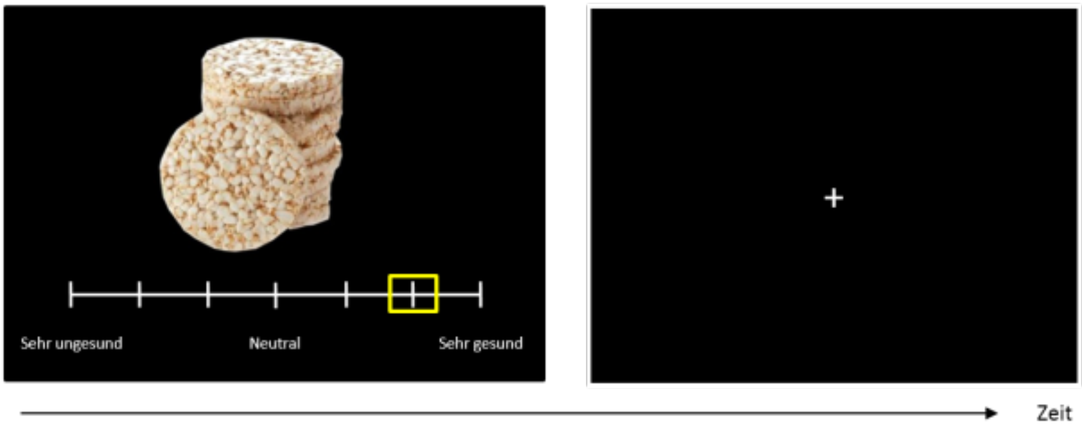

Geschmack:

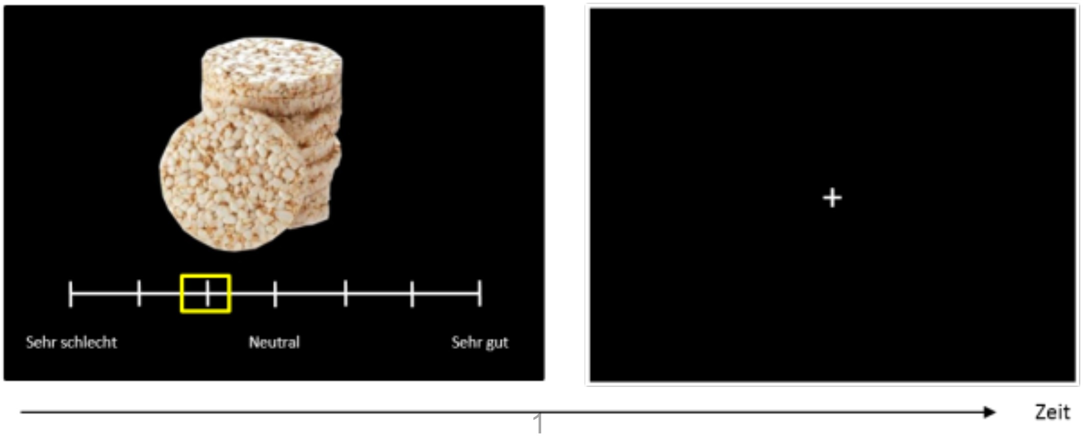

FMRT-Experiment

Das fMRT-Experiment dauert etwa 40–50 Minuten und wird auf den folgenden Seiten genauer beschrieben. Wie bereits geschildert, setzt es sich aus drei wiederkehrenden Elementen zusammen (vgl. Abbildung):

1. **Cyberball- Aufgabe**
2. **Emotionsratings**
3. **Lebensmittelentscheidungen**

Diese drei Elemente wurden bereits in der „Information zur Teilnahme“ thematisiert und werden nachfolgend detailliert beschrieben. Die folgende Abbildung zeigt den Ablauf im Scanner. Zwischen den Elementen werden „Fixationskreuze“ angezeigt – bitte schauen Sie diese beim Warten einfach an. In der folgenden Darstellung sehen Sie den zeitlichen Ablauf der angezeigten Schaubilder.

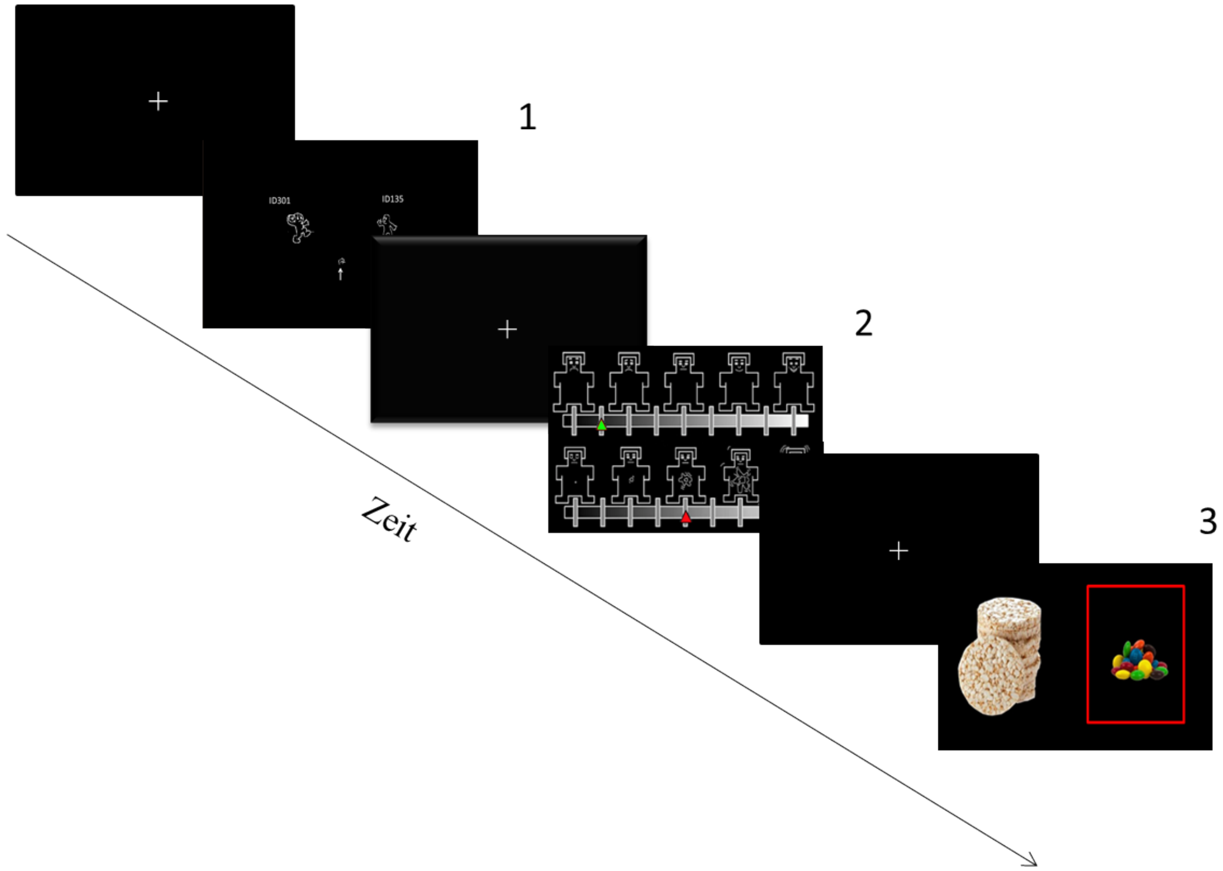

1. Cyberball-Aufgabe

Am Anfang jeder Runde spielen Sie zusammen mit zwei anderen Spielern ein Online Ballspiel. Die IDs der anderen Spieler werden im Bildschirm angezeigt, ein Pfeil zeigt Ihnen Ihre Position an (in der Mitte des Bildschirms). In jeder Runde werden Sie mit anderen Mitspielern spielen. Sie müssen einen Knopf drücken um den Ball entweder zu dem Spieler auf Ihrer rechten Seite, oder dem auf der linken Seite zu werfen. Sie können den Ball zu einem beliebigen Spieler werfen, Ihre Entscheidung sollte jedoch so schnell wie möglich erfolgen. Wenn Sie sich nicht schnell genug entscheiden, wird der Ball zufällig zu einem Ihrer Mitspieler geworfen. Wenn Sie jedoch während des gesamten Experiments aktiv mit Ihren Mitspielern spielen und immer einem von beiden den Ball zuwerfen, erhalten Sie am Ende des Experiments eine zusätzliche Auszahlung.

Hier sehen Sie eine beispielhafte Darstellung des Spiels:

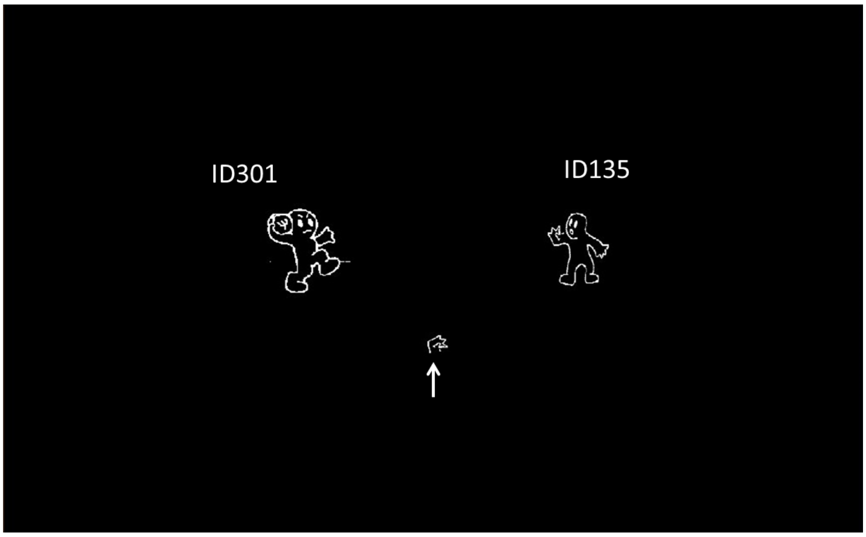

Dabei gilt folgende Tastenbelegung:

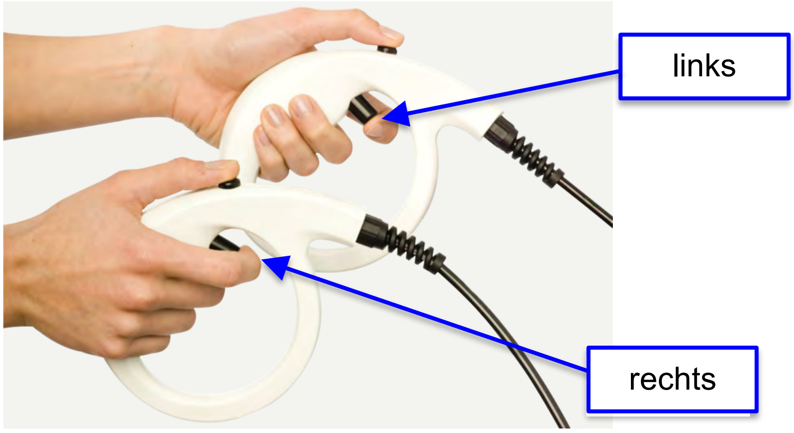

2. Emotionsratings

Anschließend findet ein Emotionsrating statt. Dabei bitten wir Sie, Ihre aktuelle Stimmungslage durch die Auswahl von Symbolen auszudrücken. Es gibt keine richtigen oder falschen Antworten.

Bei dieser Aufgabe werden Ihnen zwei Skalen angezeigt, die unterschiedliche Stimmungen darstellen: Zufriedenheit und Aufregung. Jede der Skalen zeigt 9 unterschiedliche Ausprägungen auf. Rechts und links auf der Skala befinden sich jeweils die Extreme. Versuchen Sie, sich die Bedeutung der beiden Skalen und der jeweiligen Extreme jetzt genau einzuprägen, da später im Scanner nur noch die Symbole zu sehen sind.

Skala I: „unglücklich – glücklich“

unzufrieden
unglücklich
genervt
verzweifelt
schwermütig

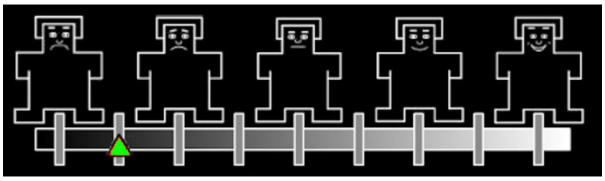

zufrieden
glücklich
erfreut
hoffnungsvoll
ausgeglichen

Skala II: „ruhig – aufgeregt“

entspannt
unerregt
ruhig

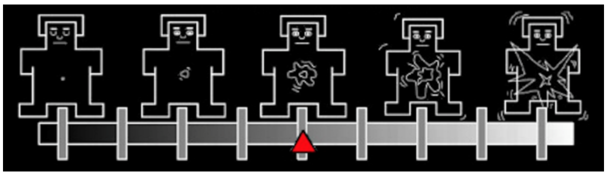

rasend
erregt
aufgeregt

Im fMRT-Experiment wird das rote Dreieck (der Cursor) zufällig an einer Stelle der ersten Skala auftauchen. Die Auswahl im Scanner treffen Sie, indem Sie die Tasten auf den MRT-Griffen betätigen. Dabei gilt folgende Tastenbelegung:

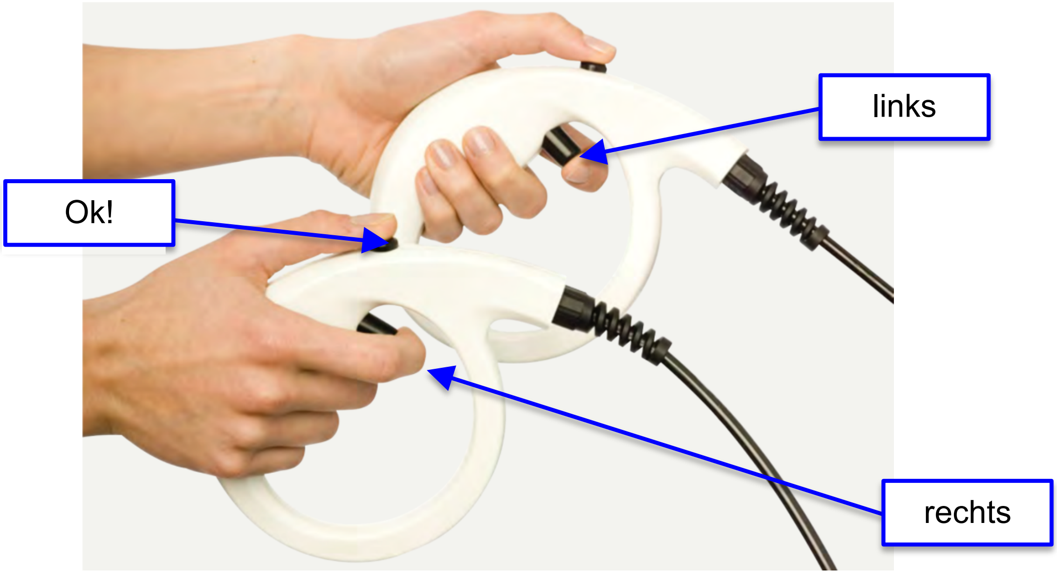

Betätigen Sie den linken und rechten Zeigefinger, um den Cursor entsprechend nach links und rechts zu verschieben. Sie bestätigen die Auswahl mit dem Knopf am rechten Daumen. Dabei färbt sich der Cursor grün als Zeichen dafür, dass Ihre Eingabe registriert wurde. Wiederholen Sie auch bei der Skala II den Vorgang auf die gleiche Weise. Da die Skalen lediglich 6 Sekunden lang angezeigt werden, ist es sehr wichtig, dass Sie ohne lang nachzudenken zügig und ehrlich Ihre Gefühlslage in dem entsprechenden Moment angeben!

3. Lebensmittelentscheidungen

Im Anschluss an jede Emotionsbewertung werden Sie zwei verschiedene Lebensmittelprodukte nebeneinander auf dem Bildschirm sehen. Nun sollen Sie sich entscheiden, welches Lebensmittel Sie in diesem Moment lieber essen möchten. Für Ihre Entscheidung haben Sie bis zu 4 Sekunden Zeit, reagieren Sie also schnell. Falls Sie mehr als 4 Sekunden benötigen, wird Ihre Antwort nicht gewertet.

Hier ein Beispiel für zwei auf einander folgende Entscheidungsrunden:

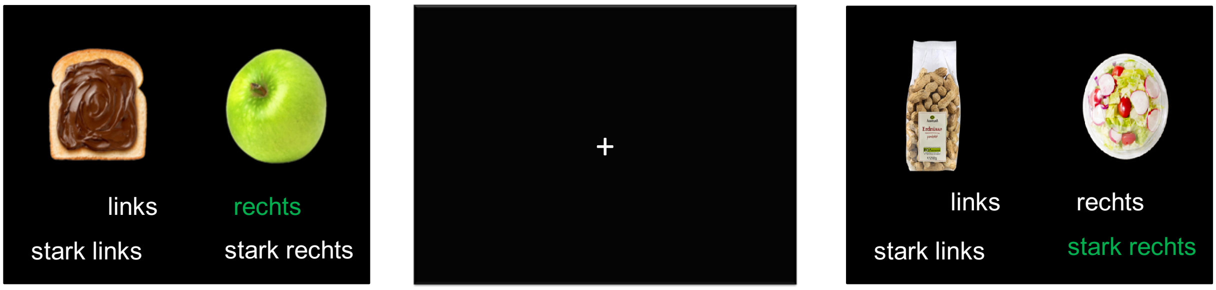

Die Auswahl im Scanner treffen Sie auch bei dieser Aufgabe, indem Sie die Tasten auf den MRT-Griffen betätigen. Dabei gilt folgende Tastenbelegung:

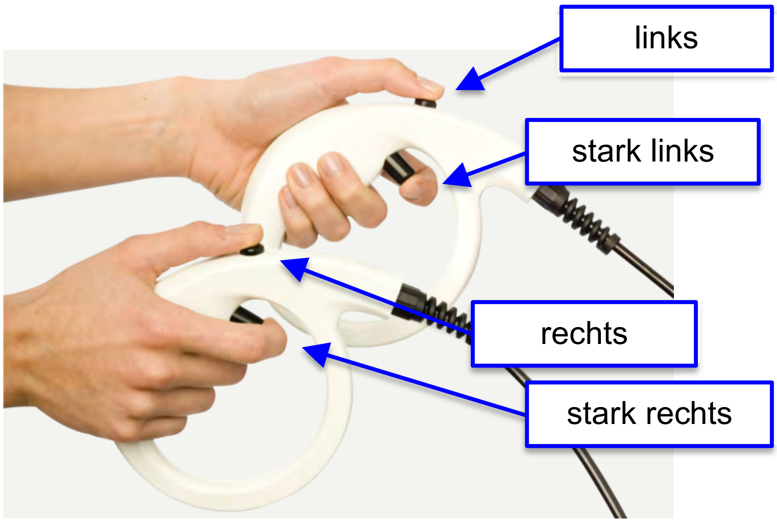

Bei Ihrer Wahl der Lebensmittel können Sie zwischen zwei Präferenzstärken wählen: starke Präferenz (Zeigefinger) links /rechts und schwächere Präferenz (Daumen) links/rechts. Für Ihre Entscheidung betätigen Sie bitte den **linken Zeigefinger, oder linken Daumen** für das linke Produkt bzw. den **rechten Zeigefinger oder rechten Daumen** für das rechte Produkt. Sobald sie den linken oder den rechten Zeigefinger oder Daumen gedrückt haben, wird die Entscheidung registriert und durch einen Farbwechsel von weiß auf grün kenntlich gemacht.

Am Ende des gesamten Experiments wird ein Durchgang zufällig gewählt und wir werden Ihnen das von Ihnen in diesem Durchgang gewählte Produkt aushändigen. Achten Sie daher darauf, in **jeder** Runde nur Produkte auszuwählen, die Sie tatsächlich gern essen möchten. Es folgen mehrere Lebensmittelentscheidungen nacheinander, bevor die nächste Runde mit der Cyberball- Aufgabe von vorne startet.

Nach dem 40–50-minütigen fMRT-Experiment wird eine strukturelle Aufnahme Ihres Gehirns erstellt, was ca. 10 Minuten in Anspruch nimmt. Während der strukturellen Messung bearbeiten Sie keine weiteren Aufgaben, Sie dürfen dabei die Augen schließen. **Bitte achten Sie darauf, Ihren Kopf während des fMRT-Experiments und der strukturellen Aufnahme nicht zu bewegen, um eine gute Bildqualität sicherzustellen! Das ist sehr wichtig für die weitere Auswertung der Daten.**

Vielen Dank für Ihre Teilnahme!
