# Supplementary material for: Do Disadvantageous Social Contexts Influence Food Choice? Evidence From Three Laboratory Experiments
Source: Front Psychol. 2020 Nov 6;11:575170. doi: 10.3389/fpsyg.2020.575170 (PMC7677191; doi:10.3389/fpsyg.2020.575170)
Supplement: Supplementary file 7 [file Data_Sheet_7.pdf]

Instructions provided to the participants in the Reaction Time Ranking experiment

As the original instructions were given in German and the emotion ratings were elicited in German, we here provide an English translation of the same.

Information about today's study

In the following we would like to inform you about the course of today's study. Please read these instructions carefully. Please contact us in case you have any questions.

In the next step, you will be given written comprehension questions about the tasks explained in the instructions. This part will last approximately 5 minutes.

Today's study consists of two parts. In the following pages you will receive some fundamental information regarding both parts of the study. Later on, you will receive more specific information about Part I and Part 2.

Information about Part 1

Food rating task

In this part of the study you will see pictures of individual food items on a computer screen. Your task is to rate the food items in terms of taste or to assess how healthy they are. This part will last around 20 to 30 minutes.

Information about Part 2

The second part consists of two recurring elements:

1. a reaction time task as well as

2. a series of food choices.

You will be given a detailed explanation of these elements at the beginning of Part 2.

Information regarding your reimbursement

At the end of the entire experiment, which will last around 1.5 hours, you will be reimbursed for your participation.

You will receive a participation fee of €15.

The second part of the reimbursement consists of the implementation of one of the food decisions that you make during the experiment. This means that you will receive one of the food items you choose in the food choice task. Since you do not know which of your decisions will be implemented, you should make each decision as if it were your only one.

We want you to feel well during and after the experiment. If you feel uncomfortable about anything, if you don’t understand something or want to know more about it, please let us know.

Please do not ask questions loudly under any circumstances! If you have questions, let us know by raising your hand. One of the investigators will attend to you as soon as possible.

Your participation in the study is voluntary. You can withdraw your consent at any time or stop the experiment at any time without giving any reasons. This will not put you at any disadvantage (except for not receiving any reimbursement).

Part 1

Now we start with Part 1.

Part 1: Food rating task

In the food rating task, you will be shown images of food items on the computer screen. You should rate each food item using the mouse and the scale displayed under the food item (see the following figure). You will rate food items in two blocks, once in terms of taste and once in terms of health. Which of the evaluation blocks (taste or health) will be queried first is determined randomly, but it will be indicated on the screen before the start of the respective block. If you are unfamiliar with the displayed food item, please make an assessment as good as you can. Please look at each food item carefully but make your decisions quickly and do not deliberate for too long. This part of the experiment takes about 20–30 minutes.

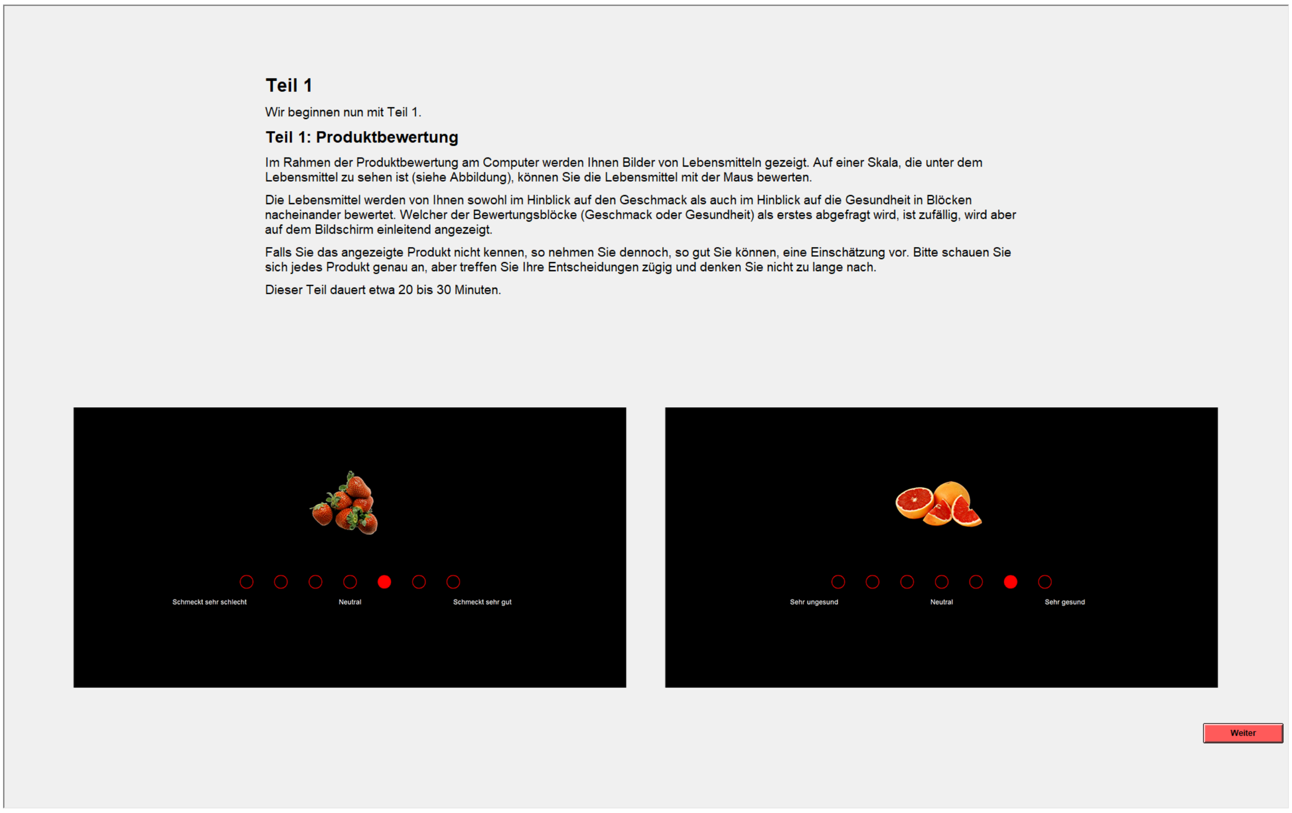

Part 1: Food rating task

The order of the two rating blocks was randomized across participants.

Block 1 [or 2]: Health

In this block, we ask you to rate the food items shown to you on the screen in terms of how healthy you think they are.

The scale for these ratings consists of 7 levels and ranges from “very unhealthy” to “very healthy”.

Block 2 [or 1]: Taste

In this block, we ask you to rate the food items shown to you on the screen in terms of how tasty you think they are.

The scale for these ratings consists of 7 levels and it ranges from “tastes very bad” to “tastes very good”.

Part 2

Information about Part 2

This part takes about 40 minutes and consists of 111 trials. The tasks comprising every trial will be described in detail in the following pages. As mentioned before, each of the 111 trials includes two recurring elements:

1. Reaction time task: At the beginning of each round, you will perform a reaction time task. A colored circle will appear at the center of the screen on a black background. Your task is to press the **left mouse button** as soon as the circle appears. (The position of the mouse pointer on the screen is irrelevant.) Your performance is determined by the speed of your reaction. After each trial, a screen will appear on which you receive feedback in the form of a ranking how fast your reaction was compared to four other participants. The ranking will include the participants’ IDs. This implies that other participants can see your reaction time as well. The four other participants are, in each round, drawn randomly from all participants who are present today.

2. Food choices: In this task, you will be displayed two different food items next to each other on the screen. You will be asked to decide which of these food items you would prefer to eat at that very moment.

The two recurring elements of Part 2 have already been mentioned in the instructions before the beginning of Part 1 and will be described in detail in the following.

The images included in the following figure illustrate the timeline of the two elements over the course of a trial.

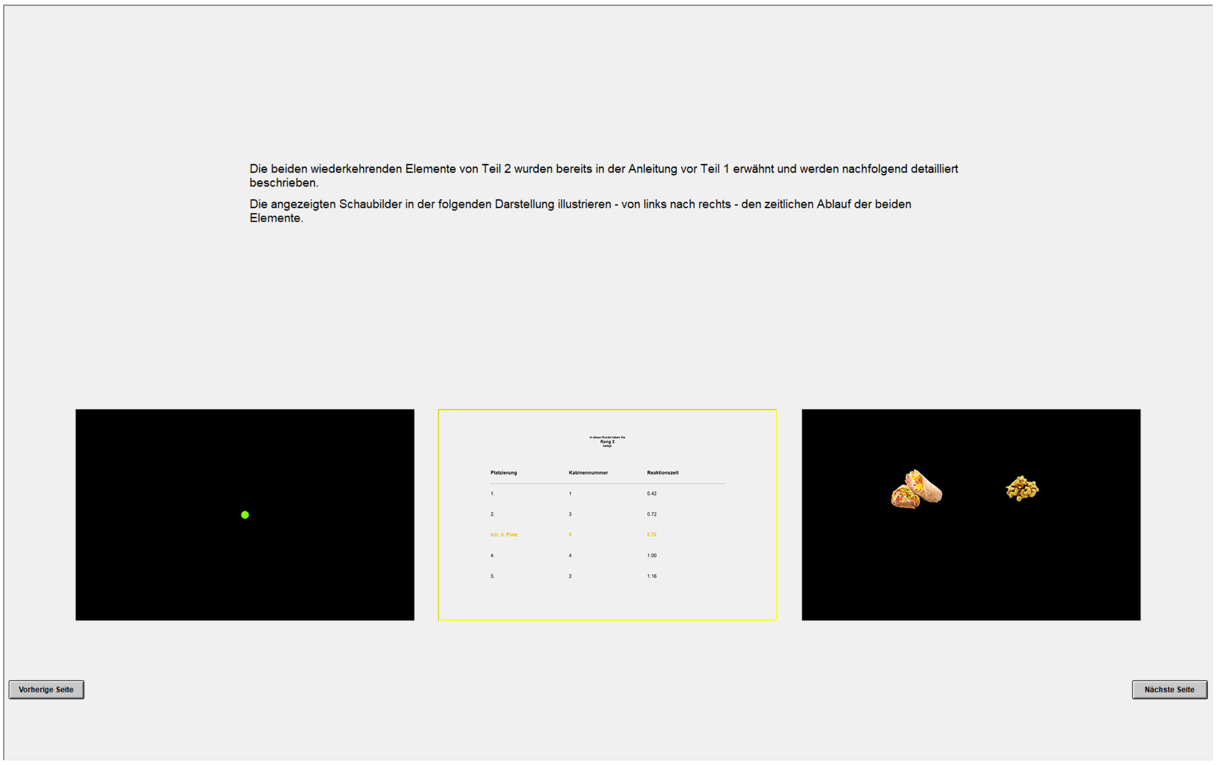

1. Reaction time task

At the beginning of each trial, you will perform a task to determine your reaction time. At the beginning of each trial the background of the screen will turn black. Shortly thereafter, a colored circle will appear on this black background. The exact time point at which the circle appears will vary randomly from trial to trial.

Your task is to click **the left mouse button** as soon as the circle appears. As soon as you have pressed the left mouse button, the colored circle disappears again—which indicates that you mouse click has been registered.

Your performance is determined by the speed of your reaction. After each trial, a screen will appear on which you receive feedback in the form of a ranking how fast your reaction was compared to four other participants. This implies that other participants can see your reaction time as well. As already mentioned, the four other participants are, in each round, drawn randomly from all participants who are present today.

If you click too early (i.e., before the circle is displayed), you will be disqualified for the corresponding round and ranked last. The maximum reaction time allowed is 1.5 seconds, and it is irrelevant where the mouse pointer is located on the screen when you press the button on the mouse.

2. Food choices

After each reactions time task, you will be displayed two different food items next to each other on the screen. You then have to decide which of these food items you would prefer to eat at that very moment.

You are given 4 seconds to make a decision; hence, please respond quickly. In case you need more than 4 seconds, your answer will not be recorded.

To indicate your decision, please do a mouse click on the image of the food item that you prefer! (The selected item will be highlighted for a short time by a red line.)

Thanks a lot for your participation!

Emotion rating questions

Participants were asked postexperimentally to indicate whether they felt satisfied (German: *zufrieden*), frustrated (German: *frustriert*), disappointed (German: *enttäuscht*), upset (German: *verärgert*), and proud (German: *stolz*) after attaining the first, third, and fifth (last) rank.

Please answer the following questions by using the scales.

1) **When you attained the first rank:**
    a. Did you feel satisfied?

Not at all								Very much
(1)	(2)	(3)	(4)	(5)	(6)	(7)	(8)	(9)
○	○	○	○	○	○	○	○	○

    b. Did you feel frustrated?

Not at all								Very much
(1)	(2)	(3)	(4)	(5)	(6)	(7)	(8)	(9)
○	○	○	○	○	○	○	○	○

    c. Did you feel disappointed?

Not at all								Very much
(1)	(2)	(3)	(4)	(5)	(6)	(7)	(8)	(9)
○	○	○	○	○	○	○	○	○

    d. Did you feel upset?

Not at all								Very much
(1)	(2)	(3)	(4)	(5)	(6)	(7)	(8)	(9)
○	○	○	○	○	○	○	○	○

    e. Did you feel proud?

Not at all								Very much
(1)	(2)	(3)	(4)	(5)	(6)	(7)	(8)	(9)
○	○	○	○	○	○	○	○	○

2) **When you attained the third rank:**
    a. Did you feel satisfied?

Not at all								Very much
(1)	(2)	(3)	(4)	(5)	(6)	(7)	(8)	(9)
○	○	○	○	○	○	○	○	○

b. Did you feel frustrated?

Not at all								Very much
(1)	(2)	(3)	(4)	(5)	(6)	(7)	(8)	(9)
○	○	○	○	○	○	○	○	○

c. Did you feel disappointed?

Not at all								Very much
(1)	(2)	(3)	(4)	(5)	(6)	(7)	(8)	(9)
○	○	○	○	○	○	○	○	○

d. Did you feel upset?

Not at all								Very much
(1)	(2)	(3)	(4)	(5)	(6)	(7)	(8)	(9)
○	○	○	○	○	○	○	○	○

e. Did you feel proud?

Not at all								Very much
(1)	(2)	(3)	(4)	(5)	(6)	(7)	(8)	(9)
○	○	○	○	○	○	○	○	○

3) When you attained the fifth rank:

a. Did you feel satisfied?

Not at all								Very much
(1)	(2)	(3)	(4)	(5)	(6)	(7)	(8)	(9)
○	○	○	○	○	○	○	○	○

b. Did you feel frustrated?

Not at all								Very much
(1)	(2)	(3)	(4)	(5)	(6)	(7)	(8)	(9)
○	○	○	○	○	○	○	○	○

c. Did you feel disappointed?

Not at all								Very much
(1)	(2)	(3)	(4)	(5)	(6)	(7)	(8)	(9)
○	○	○	○	○	○	○	○	○

d. Did you feel upset?

Not at all								Very much
(1)	(2)	(3)	(4)	(5)	(6)	(7)	(8)	(9)
○	○	○	○	○	○	○	○	○

e. Did you feel proud?

Not at all								Very much
(1)	(2)	(3)	(4)	(5)	(6)	(7)	(8)	(9)
○	○	○	○	○	○	○	○	○
