# Supplementary material for: Do Disadvantageous Social Contexts Influence Food Choice? Evidence From Three Laboratory Experiments
Source: Front Psychol. 2020 Nov 6;11:575170. doi: 10.3389/fpsyg.2020.575170 (PMC7677191; doi:10.3389/fpsyg.2020.575170)
Supplement: Supplementary file 8 [file Data_Sheet_8.pdf]

## Informationen zur heutigen Studie

**Im Folgenden möchten wir Sie über den Ablauf der heutigen Studie informieren. Bitte lesen Sie sich diese Informationen aufmerksam durch. Wenden Sie sich bitte an uns, falls Sie noch Fragen haben.**

Anschließend erwarten Sie schriftliche Verständnisfragen zu den in den Instruktionen erklärten Aufgaben. Dieser Teil dauert etwa 5 Minuten.

Die heutige Studie besteht aus zwei Teilen. Auf den folgenden Seiten erhalten Sie vorab einige grundsätzliche Informationen zu den beiden Teilen. Später erhalten Sie dann separat spezifischere Informationen für Teil 1 und Teil 2.

## Informationen zu Teil 1

### Produktbewertung

In diesem Teil der Untersuchung werden Ihnen am Computer Fotos von einzelnen Lebensmitteln präsentiert. Sie haben dabei die Aufgabe, das Produkt nach Geschmack zu bewerten oder einzuschätzen, wie gesund das Produkt Ihrer Meinung nach ist. Dieser Teil dauert etwa 20 bis 30 Minuten.

## Informationen zu Teil 2

Der zweite Teil setzt sich aus zwei wiederkehrenden Elementen zusammen:

**1. einer Aufgabe zur Bestimmung Ihrer Reaktionszeit sowie**

**2. einer Reihe von Lebensmittelentscheidungen.**

Eine detaillierte Beschreibung dieser Elemente erhalten Sie zu Beginn von Teil 2.

## Informationen zu Ihrer Auszahlung

**Am Ende des gesamten Experiments, das bis zu 1,5 Stunden in Anspruch nehmen wird, findet die Auszahlung statt.**

**Dabei erhalten Sie eine Teilnahmevergütung in Höhe von 15 €.**

Den zweiten Bestandteil der Auszahlung stellt die Umsetzung einer der von Ihnen in Teil 2 dieses Experiments getroffenen Lebensmittelentscheidungen dar. Das bedeutet, dass Sie eines der von Ihnen ausgewählten Produkte erhalten. Da Sie nicht wissen, welche Ihrer Entscheidungen umgesetzt wird, sollten Sie jede Entscheidung so treffen, als wäre sie Ihre einzige.

**Wir wollen, dass es Ihnen während und nach der Untersuchung gut geht. Wenn Ihnen irgendetwas unangenehm ist, Sie etwas nicht verstehen oder genauer wissen wollen, informieren Sie uns bitte und fragen Sie umgehend nach.**

Bitte stellen Sie Fragen auf keinen Fall laut! Melden Sie sich ggf. per Handzeichen. Einer der Versuchsleiter kommt dann an Ihren Platz.

Ihre Teilnahme an der Studie ist freiwillig. Sie können jederzeit Ihr Einverständnis zurücknehmen oder den Versuch jederzeit ohne Angabe von Gründen abbrechen. Dadurch wird Ihnen kein Nachteil (abgesehen davon, dass Sie die Teilnahmevergütung nicht in voller Höhe erhalten) entstehen.

# Teil 1

Wir beginnen nun mit Teil 1.

## Teil 1: Produktbewertung

Im Rahmen der Produktbewertung am Computer werden Ihnen Bilder von Lebensmitteln gezeigt. Auf einer Skala, die unter dem Lebensmittel zu sehen ist (siehe Abbildung), können Sie die Lebensmittel mit der Maus bewerten.

Die Lebensmittel werden von Ihnen sowohl im Hinblick auf den Geschmack als auch im Hinblick auf die Gesundheit in Blöcken nacheinander bewertet. Welcher der Bewertungsblöcke (Geschmack oder Gesundheit) als erstes abgefragt wird, ist zufällig, wird aber auf dem Bildschirm einleitend angezeigt.

Falls Sie das angezeigte Produkt nicht kennen, so nehmen Sie dennoch, so gut Sie können, eine Einschätzung vor. Bitte schauen Sie sich jedes Produkt genau an, aber treffen Sie Ihre Entscheidungen zügig und denken Sie nicht zu lange nach.

Dieser Teil dauert etwa 20 bis 30 Minuten.

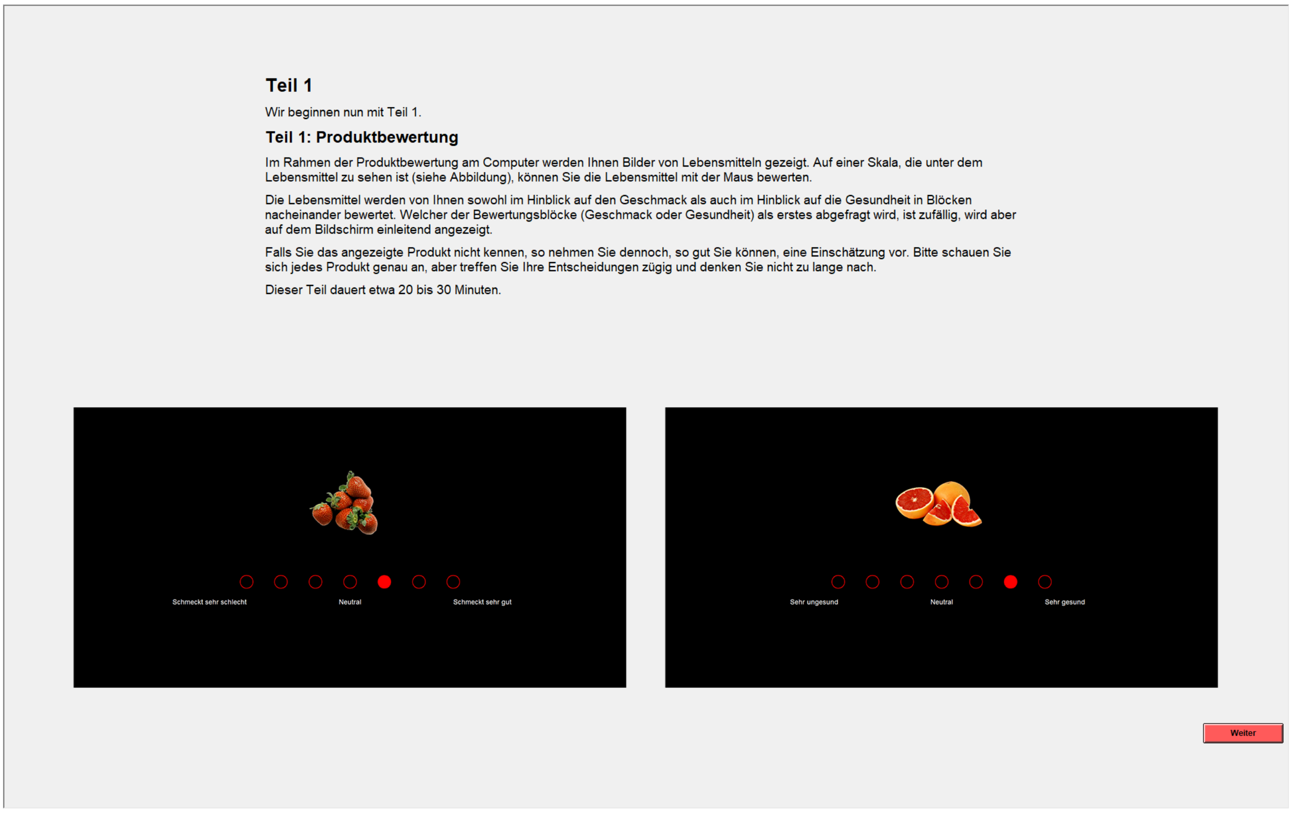

## Teil 1: Produktbewertung

*Die Reihenfolge der beiden Typen von Produktbewertungen wurde zwischen den Versuchspersonen randomisiert.*

**Block 1 [oder 2]: Gesundheit**

In diesem Block bitten wir Sie nun, anzugeben, wie gesund das auf dem Bildschirm gezeigte Produkt nach Ihrer Einschätzung ist.

Die Skala hierfür umfasst 7 Stufen und reicht von »Sehr ungesund« bis »Sehr gesund«.

**Block 2 [oder 1]: Gesundheit**

In diesem Block bitten wir Sie nun, anzugeben, wie gut Ihnen das auf dem Bildschirm gezeigte Produkt nach Ihrer Einschätzung schmeckt.

Die Skala hierfür umfasst 7 Stufen und reicht von »Schmeckt sehr schlecht« bis »Schmeckt sehr gut«.

## Teil 2

**Informationen zu Teil 2**

Dieser Teil dauert etwa 40 Minuten und besteht aus 111 Durchgängen. Die Aufgaben je Durchgang werden auf den folgenden Seiten genauer beschrieben. Wie bereits geschildert, setzt sich jeder der 111 Durchgänge von Teil 2 aus zwei wiederkehrenden Elementen zusammen:

**1. Aufgabe zur Bestimmung der Reaktionszeit:** Am Anfang jedes Durchgangs werden Sie eine Aufgabe zur Bestimmung Ihrer persönlichen Reaktionszeit ausführen. Ein farbiger Kreis wird in der Mitte des schwarzen Hintergrundes erscheinen. Ihre Aufgabe ist es, die **linke Maustaste** zu drücken, sobald der Kreis erscheint. (Es ist dabei völlig unerheblich, wo sich der Mauszeiger auf dem Bildschirm befindet.) Ihre Leistung ergibt sich aus der Schnelligkeit Ihrer Antwort. Im Anschluss an die Aufgabe erhalten Sie ein Feedback, in dem wir Ihre Leistung mit der von vier weiteren Teilnehmern/-innen vergleichen. Hierzu zeigen wir Ihnen eine Rangliste mit der ID der Teilnehmer/-innen und der jeweiligen Platzierung. Dies bedeutet gleichzeitig, dass andere Teilnehmer/-innen Ihre Reaktionszeit sehen können. Die vier anderen Teilnehmer/-innen werden in jedem Durchgang zufällig unter den Anwesenden ausgewählt.

**2. Lebensmittelentscheidungen:** Bei dieser Aufgabe werden Sie zwei verschiedene Lebensmittelprodukte nebeneinander auf dem Bildschirm sehen. Sie sollen sich entscheiden, welches Lebensmittel Sie in diesem Moment lieber essen möchten.

Die beiden wiederkehrenden Elemente von Teil 2 wurden bereits in der Anleitung vor Teil 1 erwähnt und werden nachfolgend detailliert beschrieben.

Die angezeigten Schaubilder in der folgenden Darstellung illustrieren – von links nach rechts – den zeitlichen Ablauf der beiden Elemente.

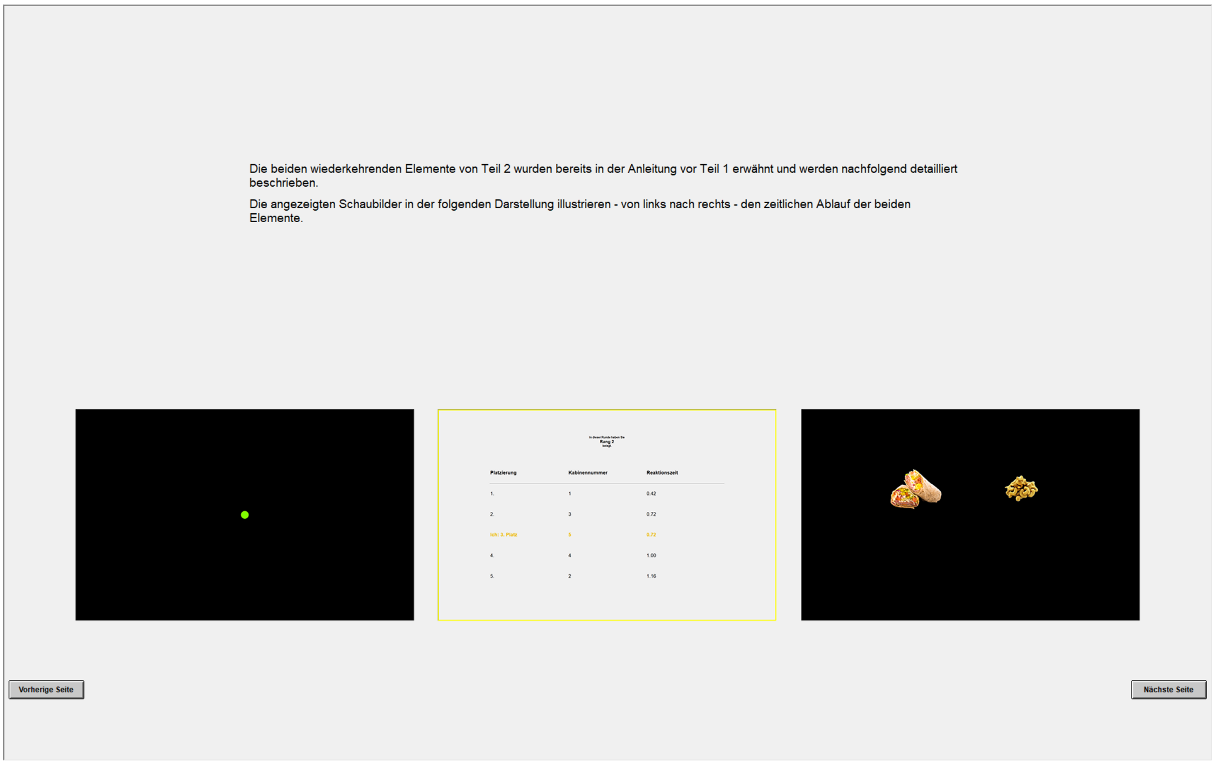


## 1. Aufgabe zur Bestimmung der Reaktionszeit

Am Anfang jedes Durchgangs werden Sie eine Aufgabe zur Bestimmung der Reaktionszeit ausführen. Zu Beginn jedes Durchgangs wird der Bildschirmhintergrund schwarz. Kurz darauf wird ein farbiger Kreis auf diesem schwarzen Hintergrund erscheinen. Der genaue Zeitpunkt des Erscheinens variiert dabei zufällig von Durchgang zu Durchgang.

Ihre Aufgabe ist stets, den **linken Mausbutton** zu drücken, sobald der Kreis erscheint. Sobald Sie die linke Maustaste gedrückt haben, verschwindet der farbige Kreis wieder – woran Sie erkennen können, dass Ihr Mausklick registriert wurde.

Ihre Leistung ergibt sich dabei aus der Schnelligkeit Ihrer Reaktion. Nach jeder Antwort erscheint ein Bildschirm, auf dem Sie ein Feedback in Form einer Rangliste darüber erhalten, wie schnell Ihre Reaktion im Vergleich zu vier anderen Teilnehmern/-innen ausgefallen ist. Andere Teilnehmer/-innen sehen somit auch Ihre Reaktionszeit. Wie bereits gesagt, werden Ihnen diese vier anderen Teilnehmer/-innen in jedem Durchgang zufällig neu aus den Anwesenden zugewiesen.

Sollten Sie zu früh klicken (also bevor der Kreis angezeigt wird), werden Sie für die entsprechende Runde disqualifiziert und auf dem letzten Rang eingeordnet. Die maximale erlaubte Reaktionszeit beträgt 1,5 Sekunden, und es ist egal, wo sich der Mauszeiger befindet, wenn Sie die Maustaste drücken.

## 2. Lebensmittelentscheidungen

Im Anschluss an jede Reaktionszeitmessung werden Sie zwei verschiedene Lebensmittelprodukte nebeneinander auf dem Bildschirm sehen. Nun sollen Sie sich entscheiden, welches Lebensmittel Sie in diesem Moment lieber essen möchten.

Für Ihre Entscheidung haben Sie bis zu 4 Sekunden Zeit, reagieren Sie also bitte zügig. Falls Sie mehr als 4 Sekunden benötigen, wird Ihre Antwort nicht gewertet.

Klicken Sie für Ihre Entscheidung mit der Maus **auf das Bild** des von Ihnen bevorzugten Lebensmittels! (Das gewählte Produkt wird nach erfolgreichem Klick kurzzeitig rot unterstrichen.)

**Vielen Dank für Ihre Teilnahme!**
